# Author Correction: First-line modified FOLFOX plus/minus nivolumab and Ipilimumab or FLOT plus nivolumab in advanced gastroesophageal adenocarcinoma: a phase II multi-cohort IKF-AIO-MOONLIGHT trial

**DOI:** 10.1038/s41467-026-72255-5

**Published:** 2026-04-17

**Authors:** Sylvie Lorenzen, Thorsten Oliver Goetze, Peter C. Thuss-Patience, Jorge Riera-Knorrenschild, Eray Goekkurt, Tobias Nicolaas Dechow, Thomas Jens Ettrich, Ralf Dieter Hofheinz, Kim Barbara Luley, Daniel Pink, Udo Lindig, Gunnar Folprecht, Gunter Schuch, Michael Bitzer, Volker Heinemann, Stefan Angermeier, Claus Bolling, Maria Loose, Sabine Junge, Claudia Pauligk, Salah-Eddin Al-Batran

**Affiliations:** 1 TUM University Hospital, Technical University of Munich, Department of Medicine III, Munich, Germany; 2 The Frankfurt Institute of Clinical Cancer Research and Krankenhaus Nordwest, University Cancer Center Frankfurt, Frankfurt, Germany; 3 Charité–Universitätsmedizin Berlin, Medizinische Klinik mit Schwerpunkt Hämatologie, Onkologie und Tumorimmunologie, Berlin, Germany; 4 Universitätsklinikum Marburg, Klinik für Innere Medizin, Marburg, Germany; 5 Hämatologisch-Onkologische Praxis Eppendorf (HOPE) and Universitäres Cancer Center Hamburg (UCCH), Hamburg, Germany; 6 Onkologie Ravensburg, Ravensburg, Germany; 7 Ulm University Hospital, Department of Internal Medicine I, Ulm, Germany; 8 Universitätsmedizin Mannheim, Tagestherapiezentrum am ITM, Mannheim, Germany; 9 University Hospital Schleswig-Holstein, Campus Luebeck, Lübeck, Germany; 10 Klinik und Poliklinik für Innere Medizin C, Hämatologie und Onkologie, Transplantationszentrum, Palliativmedizin, Universität Greifswald and Klinik für Hämatologie, Onkologie und Palliativmedizin-Sarkomzentrum, HELIOS Klinikum Bad Saarow, Bad, Saarow Germany; 11 Universitätsklinikum Jena, Klinik für Innere Medizin II, Jena, Germany; 12 Universitätsklinikum Carl Gustav Carus, Medizinische Klinik I, Dresden, Germany; 13 Hämatologisch-Onkologische Praxis Altona (HOPA), Hamburg, Germany; 14 Universitätsklinikum Tübingen, Medizinische Klinik I, Tuebingen, Germany; 15 Klinikum der Universität München-Großhadern, Medizinische Klinik III, München, Germany; 16 Klinikum Ludwigsburg, Klinik für Innere Medizin, Gastroenterologie, Hämato-Onkologie, Pneumologie, Diabetologie und Infektiologie, Ludwigsburg, Germany; 17 AMEOS Rehaklinikum Ratzeburg, Germany and Agaplesion Markus Krankenhaus, Hämatologie/Onkologie, Frankfurt, Germany; 18 The Frankfurt Institute of Clinical Cancer Research, Frankfurt, Germany

**Keywords:** Gastric cancer, Immunology, Cancer therapy

Correction to: *Nature Communications* 10.1038/s41467-026-69622-7, published online 27 February 2026

In the version of this article initially published, funding information did not acknowledge the sponsor of the trial is the Frankfurt Institute of Clinical Cancer Research IKF GmbH and that financial support and drug supply for the conduct of the trial was granted by Bristol-Myers Squibb GmbH & Co. KGaA, while the title was incomplete and did not reference the Institut für Klinische Krebsforschung and Arbeitsgemeinschaft Internistische Onkologie study groups. The title and text are amended in the HTML and PDF versions of the article.

