# Supplementary material for: First-line modified FOLFOX plus/minus nivolumab and Ipilimumab or FLOT plus nivolumab in advanced gastroesophageal adenocarcinoma: a phase II multi-cohort IKF-AIO-MOONLIGHT trial
Source: Nat Commun. 2026 Feb 27;17:2072. doi: 10.1038/s41467-026-69622-7 (PMC12948978; doi:10.1038/s41467-026-69622-7)
Supplement: Supplementary file 1 — Supplementary information [file 41467_2026_69622_MOESM1_ESM.pdf]

Supplementary information

Supplementary Note 1

Supplementary figures

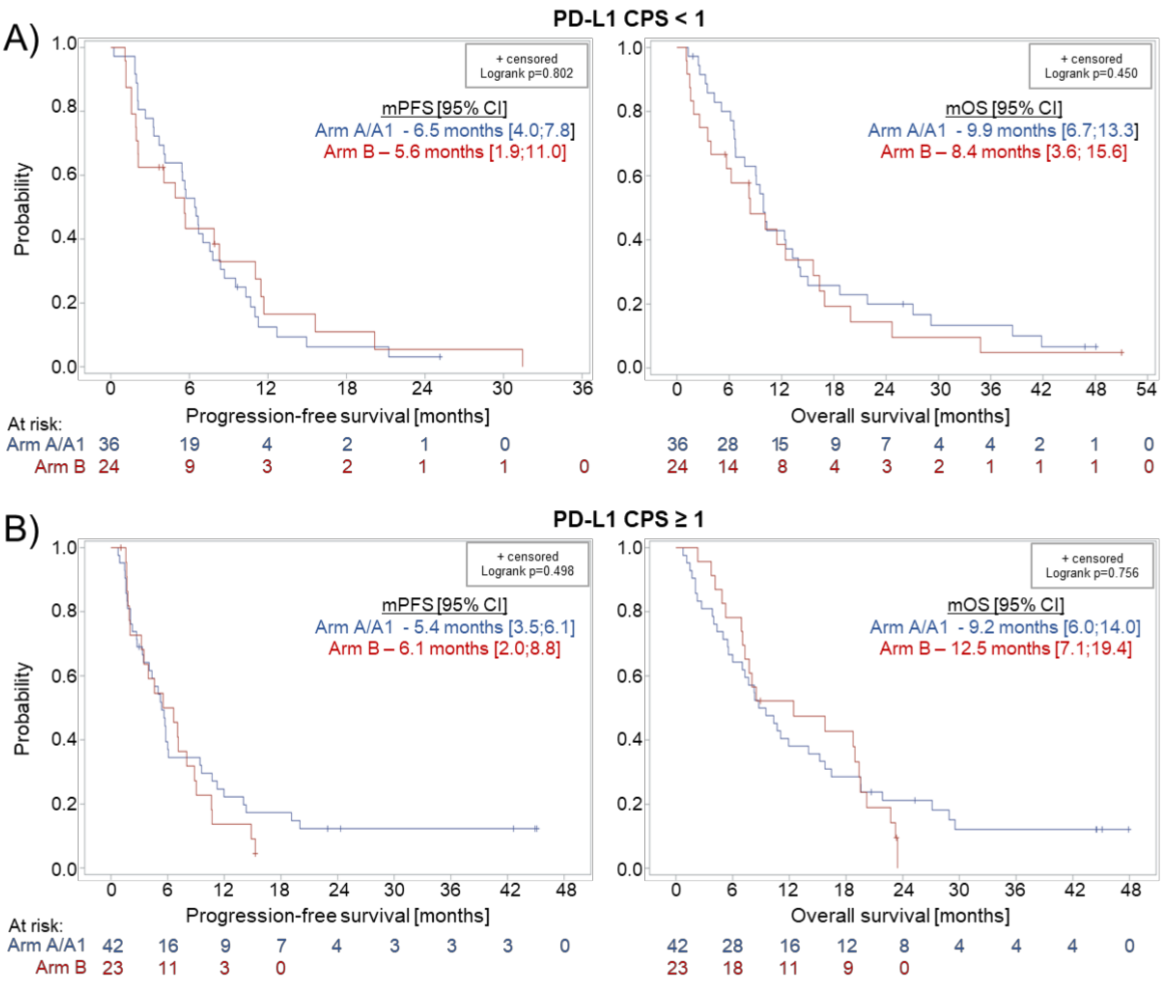

Figure S1 Progression-free and overall survival in PD-L1 subgroups Arm A/A1 vs. Arm B

Kaplan-Meier estimates of progression free and overall survival in patients treated with mFOLFOX plus nivolumab/ipilimumab in parallel (Arm A/A1) versus patients treated with mFOLFOX alone (Arm B) in patient subgroups with PD-L1 CPS < 1 (A) and PD-L1 CPS ≥ 1 (B). Source data are provided as a Source Data file.

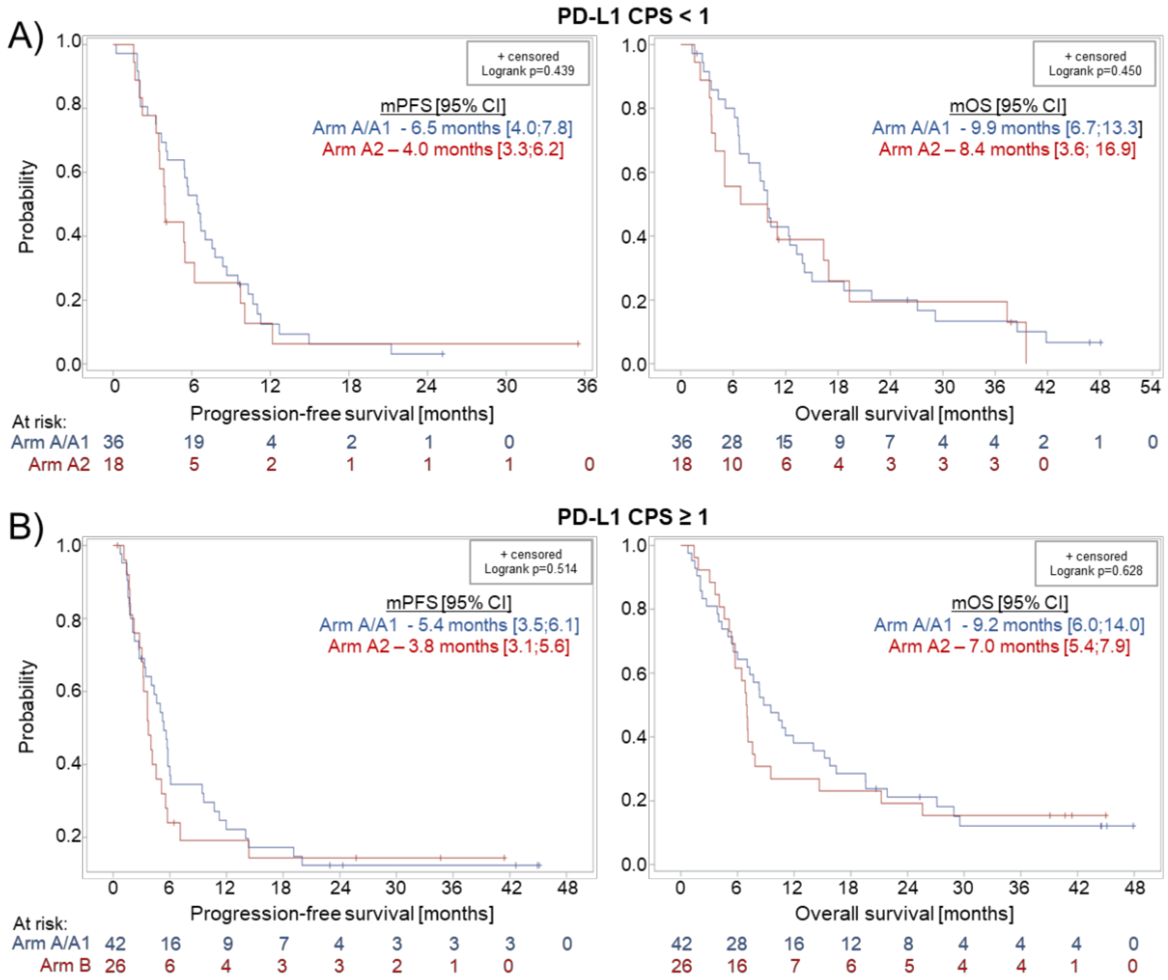

Figure S2 Progression-free and overall survival in PD-L1 subgroups Arm A/A1 vs. Arm A2

Kaplan-Meier estimates of progression free and overall survival in patients treated with mFOLFOX plus nivolumab/ipilimumab in parallel (Arm A/A1) versus patients treated sequential with mFOLFOX plus nivolumab/ipilimumab (Arm A2) in patient subgroups with PD-L1 CPS < 1 (A) and PD-L1 CPS ≥ 1 (B). Source data are provided as a Source Data file.

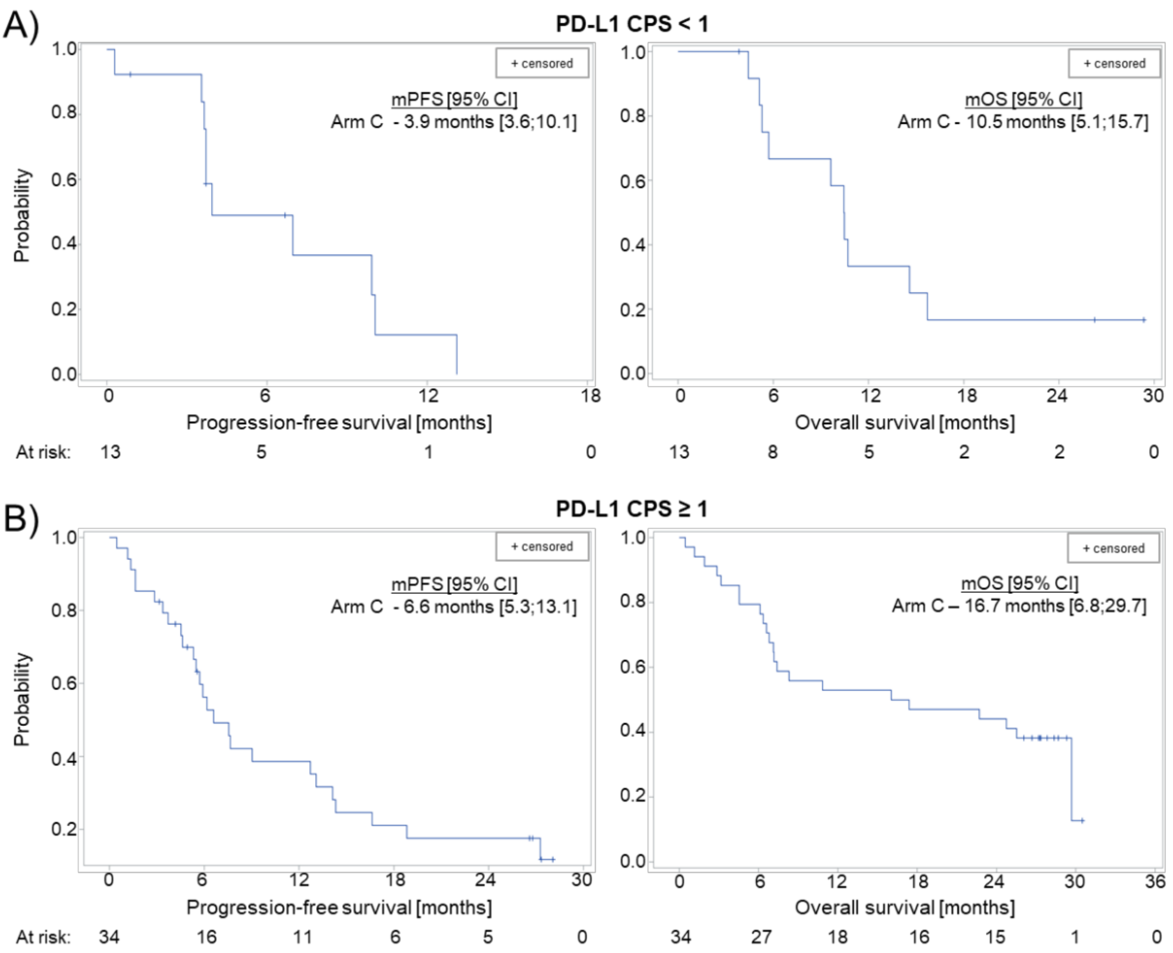

Figure S3 Progression-free and overall survival in PD-L1 subgroups Arm C

Kaplan-Meier estimates of progression free and overall survival in patients treated with FLOT plus nivolumab (Arm C) in patient subgroups with PD-L1 CPS < 1 (A) and PD-L1 CPS ≥ 1 (B). Source data are provided as a Source Data file.

Supplementary Tables

Table S1 Treatment exposure to trial drugs per treatment arm

	**Arm A/A1** n=90	**Arm A2** n=60	**Arm B** n=60	**Arm C** n=52
Median No. [range] of total treatment cycles	10 [0-48]	8 [1-52]	9 [1-48]	9.5 [1-52]
Median No. of Nivolumab / Ipilimumab cycles	10 / 3	4 / 2	NA	8.5 / NA
Duration of study treatment in months				
median	5.1	3.3	4.4	4.4
range	0 – 24.6	0.1 – 24.3	0 – 23.0	0 – 27.4
Q1-Q3	1.9 – 9.0	2.1 – 6.0	1.8 – 7.5	2.8 – 8.1
No. of cycles of nivolumab				
median	10	4	NA	8.5
range	1 - 48	1 – 49	NA	1 - 52
Q1-Q3	4 - 16	3.5 - 8	NA	5.5 - 15
No. of cycles of ipilimumab				
median	3	2	NA	NA
range	1 - 16	1 – 17	NA	NA
Q1-Q3	2 - 5	1.5 - 4	NA	NA
No. of cycles of oxaliplatin				
median	7	3	7.5	6
range	1 - 15	1 - 12	1 - 30	1 - 12
Q1-Q3	4 - 10	3 – 5.5	4 - 11	4 - 8
No. of cycles of 5-flourouracil				
median	9	3	8.5	8
range	1 - 47	1 - 21	1 - 48	1 - 46
Q1-Q3	4 - 14	3 - 5	4 - 14	4.5 – 11.5
No. of cycles of leucovorin				
median	10	3	9	8
range	1 - 47	1 - 21	1 - 48	1 - 46
Q1-Q3	4 - 15	3 – 5.5	4 – 14.5	5 – 11.5
No. of cycles of docetaxel				
median	NA	NA	NA	7
range	NA	NA	NA	1 - 16
Q1-Q3	NA	NA	NA	4 - 8

*calculated on the number of cycles actually started by the patient

Legend: N, number; Q, quartile; NA, not applicable.

Supplementary Note 2

Study protocol

German Gastric Group at AIO

Modified FOLFOX plus/minus Nivolumab and Ipilimumab vs. FLOT plus Nivolumab in patients with previously untreated advanced or metastatic adenocarcinoma of the stomach or gastroesophageal junction – A randomized phase 2 trial.

**EudraCT:**	**2017-002080-18**
**ClinicalTrials.gov ID:**	**NCT03647969**
**Sponsor's Protocol Code:**	**AIO-STO-0417**
	**CA209-9KG**
**AIO Study Number:**	**AIO-STO-0417**

Lead Coordinating Investigator

Prof. Dr. med. Thorsten O.Götze

Krankenhaus Nordwest
Steinbacher Hohl 2-26
60488 Frankfurt am Main

Tel.: +49 69 7601-4420; Fax -3655
Email: goetze.thorsten@khnw.de

Deputy Coordinating Investigator

Prof. Dr. med. Sylvie Lorenzen

III. Medizinische Klinik
des Klinikums rechts der Isar
Ismaninger Str 22
81675 München

Tel.: +49 89 / 4140-9696; Fax -4879
Email: Sylvie.Lorenzen@mri.tum.de

Sponsor Representative / Sponsor Scientific Lead

Prof. Dr. med. Salah-Eddin Al-Batran

Institut für Klinische Krebsforschung IKF GmbH
at Krankenhaus Nordwest
Steinbacher Hohl 2-26
60488 Frankfurt am Main, Germany

Tel.: +49 69 7601-4420; Fax -3655
Email: albatran@ikf-khnw.de

Steering committee

Eray Gökkurt, Hamburg
Ralf-Dieter Hofheinz, Mannheim
Nils Homann, Wolfsburg
Sylvie Lorenzen, München
Harald Schmalenberg, Dresden
Alexander Stein, Hamburg

Clinical Trial Management

Dr. rer. physiol. Claudia Pauligk

Institut für Klinische Krebsforschung IKF GmbH
at Krankenhaus Nordwest
Steinbacher Hohl 2-26
60488 Frankfurt am Main
Tel.: +49 69 7601-3906
Fax: +49 69 7601-3655
Email: pauligk.claudia@ikf-khnw.de

Legal sponsor

Institut für Klinische Krebsforschung IKF GmbH
at Krankenhaus Nordwest
Steinbacher Hohl 2-26
60488 Frankfurt/Main
www.ikf-nordwest.de

Biostatistics

Axel Hinke
CCRC Cancer Clinical Research Consulting
Elly-Heuss-Knapp-Str. 36
40595 Düsseldorf
Tel: +49 211 7000675
Fax: +49 211 7004196
Marina Schaaf
Institut für Klinische Krebsforschung IKF GmbH
at Krankenhaus Nordwest
Steinbacher Hohl 2-26
60488 Frankfurt am Main
Tel: +49 69 7601-3289

Data management

Trium Analysis Online GmbH
Hohenlindenerstr. 1
81677 München
Tel: +49 89 2060 269 0
Email: info@trium.de

Institut für Klinische Krebsforschung IKF GmbH
at Krankenhaus Nordwest
Steinbacher Hohl 2-26
60488 Frankfurt am Main
Tel: +49 69 7601-4420

Monitoring

Karin Scheffler
MCA Berlin
Grunewaldstr. 14
10823 Berlin
Tel: +49 30 21 96 27 19
Fax: +49 30 21 96 27 16
Email: k.scheffler@mca24.de

Institut für Klinische Krebsforschung IKF GmbH
at Krankenhaus Nordwest
Steinbacher Hohl 2-26
60488 Frankfurt am Main

Central Pharmacy

Marc Schrott
Medicoline Pharma Solutions KG
Bahnstr. 51
61449 Steinbach/Taunus
Tel.: +49 6171-9161-201
Fax: +49 6171-9161-218
Email: schrott@ihreapotheker.de

Independent Data Monitoring Committee

Prof. Dr. med. G.-Andre Banat, Bad Nauheim
Prof. Dr. Iris Burkholder, Zweibrücken
Prof. Dr. med. Dr. phil. Fuat Oduncu, München

Institutional Review Board (Ethics Committee)

Ethik-Kommission bei der Landesärztekammer Hessen
Hanauer Landstraße 152
60314 Frankfurt am Main
Email: ethikkommission@laekh.de

Approval of the Protocol

Coordinating Investigator (LKP according to AMG)

Prof. Dr. Thorsten O. Götze

FFM 13.7.22

Place and Date

Signature

Coordinating Investigator

Prof. Dr. Sylvie Lorenzen

[illegible], 13.07.22

Place and Date

Signature

Sponsor Representative

Prof. Dr. Salah-Eddin Al-Batran

Frankfurt 13.07.2022

Place and Date

Signature

Site / Principal Investigator / Deputy

Site: ________________________________ Site-No.:__________

Principal Investigator (Print name): _______________________________

I have read and checked the protocol diligently; I comply with the stated demands and requirements and agree to conduct the study according to the guidelines of Good Clinical Practice (GCP) and the requirements of the responsible authorities in regard to reconciliation of original data and to audits/inspections.
I consent to report every serious clinical adverse event to the sponsor within one working day, whether it is related to study medication or not.
I agree to train the members of the study group at this study site on the contents of the new protocol version and amendment.

________________________________ _______________________________________

Place and Date Signature Principal Investigator

Deputy (Print name): _______________________________________

________________________________ _______________________________________

Place and Date Signature Deputy

Study Glossary

Abbreviation/ Acronym	Definition
ADL	Activities of daily living
ADR	Adverse Drug Reaction
AE	Adverse event
AIDS	Acquired immunodeficiency syndrome
ANC	Absolute neutrophil count
ASCO	American Society of Clinical Oncology
ALT (SGPT)	Alanine aminotransferase (serum glutamic-pyruvic transaminase)
AST (SGOT)	Aspartate aminotransferase (serum glutamic-oxaloacetic transaminase)
BSA	Body surface area
BSC	Best supportive care
CA 19-9	Carbohydrate antigen 19-9
CD	Cluster of differentiation
CEA	Carcinoembryonic antigen
CNS	Central nervous system
CT	Computerized tomography
CTCAE	Common Terminology Criteria for Adverse Events
CTLA4	Cytotoxic T-lymphocyte-associated Protein 4
CTx	Chemotherapy
DPD	Dihydropyrimidine dehydrogenase
EBV	Epstein Barr Virus
ECG	Electrocardiogram
ECOG	Eastern Cooperative Oncology Group
eCRF	Electronic Case Report Form
EDTA	Ethylenediamine tetraacetic acid
EGA	Esophagogastric adenocarcinoma
EGFR	Epidermal Growth Factor Receptor
EOT	End of Treatment
FDA	Food and Drug Administration (U.S. government agency)
FFPE	Formalin-fixed, paraffin-embedded
FLOT	Fluorouracil, Leucovorin, Oxaliplatin, Docetaxel
FSH	Follicle stimulating hormone
GC	Gastric cancer
GCP	Good Clinical Practice
GCP-V	Verordnung über die Anwendung der Guten Klinischen Praxis (GCP) bei der Durchführung von klinischen Prüfungen mit Arzneimitteln zur Anwendung am Menschen

GEJ	Gastroesophageal junction
HER	Human epidermal growth factor receptor
HIV	Human immunodeficiency virus
HR	Hazard ratio
HRT	Hormone replacement therapy
IB	Investigator Brochure
ICF	Informed consent form
IDMC	Independent Data Monitoring Committee
IEC	Independent ethics committee
IGKC	Immunoglobulin Kappa Constant
IHC	Immunohistochemistry
IMP	Investigational medicinal product
INR	International normalized ratio
ITT	Intention-to-treat
IV	intravenous
LDH	Lactate dehydrogenase
LFT	Liver function test
MMP	Matrix metalloproteinase
MRI	Magnetic resonance imaging
mRNA	Messenger RNA
MSI	Microsatellite Instability
NCCN	National Comprehensive Cancer Network
NCI	National Cancer Institute
NGS	Next generation sequencing
NYHA	New York Heart Association
ORR	Overall Response Rate
OS	Overall survival
PD	Progressive Disease
PD-L1	Programmed Death Receptor Ligand 1
PET	Positron emission tomography
PFS	Progression Free Survival
PR	Partial Response
PTT	Partial thromboplastin time
QoL	Quality of life
RBC	Red blood cell count
RDE	Remote data entry
RECIST	Response Evaluation Criteria in Solid Tumors
RTK	Receptor tyrosine kinase
RT-PCR	Reverse transcription polymerase chain reaction
SADR	Serious adverse drug reaction

SAE	Serious adverse event
SAR	Seriour adverse reaction
SAS	Statistic software
SD	Stable Disease
SDV	Source Data Verification
SLD	Sum of the longest diameters
SmPC	Summary of Product Characteristics
SUSAR	Suspected Unexpected Serious Adverse Reaction
TIL	Tumor-infiltrating lymphocytes
TNM	Classification of malignant tumors
TSH	Thyroid-Stimulating Hormone
TTSD	Time to symptom deterioration
ULN	Upper limit of normal
WBC	White blood cell count
WOCBP	Women of childbearing potential

Table of Contents

APPROVAL OF THE PROTOCOL 3

STUDY GLOSSARY 5

TABLE OF CONTENTS 8

SYNOPSIS 11

1. INTRODUCTION AND BACKGROUND 28

1.1 EPIDEMIOLOGY AND DISEASE BACKGROUND 28
1.2 RATIONALE FOR CHECKPOINT INHIBITION IN ESOPHAGOGASTRIC ADENOCARCINOMA 29
1.2.1 Single Agent PD-1 Inhibition 29
1.2.2 Combination of PD-1 and CTLA-4 Receptor Blockade 30
1.3 RATIONALE FOR INCLUDING BOTH SUBJECTS WITH PD-L1 AND NON-PD-L1 EXPRESSING TUMORS IN 1L GC/GEJ 31
1.4 RATIONALE FOR CHOICE OF COMPARATOR 32
1.5 RATIONALE FOR THE COMBINATION OF CHECKPOINT INHIBITION AND CHEMOTHERAPY 32
1.6 RATIONALE FOR ALTERNATING CHECKPOINT INHIBITION WITH CHEMOTHERAPY IN ESOPHAGOGASTRIC ADENOCARCINOMA 33
1.7 RATIONALE FOR THE COMBINATION OF CHECKPOINT INHIBITION AND TRIPLET CHEMOTHERAPY (ARM C-FLOT COHORT) 33
1.8 OVERALL RISK/BENEFIT ASSESSMENT 34

2. STUDY OBJECTIVE 35

3. STUDY DESIGN 35

3.1 PRIMARY ENDPOINT 35
3.2 SECONDARY ENDPOINTS 36

4. STUDY POPULATION 36

4.1 NUMBER OF PATIENTS 36
4.2 SELECTION CRITERIA 36
4.2.1 Inclusion criteria 36
4.2.2 Exclusion criteria 39
4.2.3 Women of Childbearing Potential 40

5. STUDY PROCEDURES AND METHODOLOGY 41

5.1 OVERALL STUDY SCHEDULE OVERVIEW 41
5.2 TREATMENT 42
5.2.1 Randomization/enrolment 42
5.2.2 Investigational Product 42
5.2.3 Dosing and schedule 43
5.2.4 Treatment duration 47
5.2.5 Study medication 47
5.2.5.1 Handling and Dispensing 48
5.2.5.2 Treamtent administration and study site qualification 48
5.2.5.3 Destruction 49
5.2.6 Concomitant medication 49
5.2.6.1 Permitted therapy 49
5.2.6.2 Prohibited Therapy 49
5.3 ASSESSMENTS AND GUIDELINES FOR VISITS 50
5.3.1 Baseline assessments (within 4 weeks before treatment start) 50
5.3.2 Assessments during treatment 51
5.3.2.1 Assessment at start of treatment and every 2 weeks (+3/-2 days) thereafter 51
5.3.2.2 Tumor response assessment (every 8 weeks ±7 days) 52
5.3.2.3 Final staging (end of treatment) 52
5.3.3 Extended safety follow-up: 30 days and 100 day safety follow-up (±7 days) 52
5.3.4 Individual Follow-up 53
5.4 STUDY DURATION 53
5.5 END OF TRIAL 53
5.6 STUDY TERMINATION 54

5.6.1 *Interim Safety Analysis* ... 54
5.6.2 *Patient Withdrawal* ... 54
5.6.3 *Study Completion* ... 55

6. DOSE MODIFICATIONS ... 55

6.1 GENERAL REMARKS ... 55
6.1.1 *Dose Delay Criteria* ... 56
6.1.2 *Criteria to Resume Treatment* ... 57
6.1.3 *Management Algorithms* ... 58
6.1.4 *Discontinuation Criteria* ... 58
6.1.5 *Dose Modification Criteria for Docetaxel, Oxaliplatin-Plus-Fluoropyrimidine Treatment* ... 59
... 61
6.1.6 *Discontinuation Criteria for FOLFOX* ... 62
6.1.7 *Discontinuation Criteria for FLOT* ... 62
6.1.8 *Treatment Infusion Reactions related to Nivolumab or Ipilimumab* ... 63

7. CRITERIA OF EVALUATION ... 64

7.1 PROGRESSION FREE SURVIVAL (PFS) ... 64
7.2 OVERALL RESPONSE RATE (ORR) ... 64
7.3 OVERALL SURVIVAL (OS) ... 64
7.4 SAFETY ENDPOINTS ... 64
7.5 QUALITY OF LIFE ASSESSMENT (EORTC QLQ-C30) ... 65

8. TRANSLATIONAL RESEARCH ... 65

8.1 TRANSLATIONAL RESEARCH PROJECTS ... 65
8.2 SAMPLING TIME POINTS AND MATERIALS ... 65
8.3 SAMPLE STORAGE AND ARCHIVING ... 66

9. ASSESSMENT OF ADVERSE EVENTS & SAFETY REPORTING ... 66

9.1 REFERENCE SAFETY DOCUMENTS ... 66
9.2 ADVERSE EVENTS DEFINITIONS ... 67
9.2.1 *Adverse Event* ... 67
9.2.2 *Serious Adverse Event* ... 67
9.2.3 *Other reportable events* ... 68
9.2.4 *Unexpected Adverse Event* ... 68
9.3 ASSESSMENT OF RELATIONSHIP - ADVERSE DRUG REACTION ... 69
9.4 ASSESSMENT OF SEVERITY ... 69
9.5 SAFETY RECORDING AND REPORTING REQUIREMENTS ... 70
9.5.1 *Recording periods* ... 70
9.5.2 *Recording and Reporting requirements* ... 70
9.5.3 *Sponsor obligations* ... 72
9.6 HANDLING OF SAFETY PARAMETERS ... 73
9.6.1 *Adverse events* ... 73
9.6.2 *Treatment and Follow-up of Adverse Events* ... 73
9.6.3 *Follow-up of Abnormal Laboratory Test Values* ... 73
9.6.4 *Overdose* ... 74
9.6.5 *Drug induced liver injury* ... 74
9.6.6 *Pregnancies and contraception* ... 74
9.6.7 *Adverse Drug reactions with Concomitant Medication* ... 76
9.7 INDEPENDENT DATA MONITORING COMMITTEE (IDMC) ... 76

10. DATA ANALYSIS AND STATISTICAL CONSIDERATIONS ... 76

10.1 SAMPLE SIZE CALCULATION ... 76
10.2 POPULATIONS FOR ANALYSIS ... 78
10.3 PATIENT DEMOGRAPHICS/OTHER BASELINE CHARACTERISTICS ... 79
10.4 TREATMENTS (STUDY TREATMENTS) ... 79
10.5 EFFICACY ANALYSIS ... 79
10.5.1 *Primary Efficacy Endpoint* ... 79
10.5.2 *Secondary Efficacy Endpoints* ... 80

10.5.3 Safety analyses (toxicity) ... 80

11. DATA MANAGEMENT ... 80

11.1 RANDOMIZATION/ENROLMENT PROCEDURE ... 80
11.2 PATIENT IDENTIFICATION LIST ... 81
11.3 DATA CAPTURE ... 81

12. QUALITY ASSURANCE ... 81

12.1 STANDARDIZATION ... 81
12.2 DATA ACCESS ... 81
12.3 MONITORING/ SOURCE DATA VERIFICATION (SDV) ... 81
12.4 AUDITS AND INSPECTIONS ... 82

13. REGULATORY AND LEGAL OBLIGATIONS ... 82

13.1 GENERAL PROVISIONS/DECLARATION OF HELSINKI ... 82
13.2 PATIENT PROTECTION ... 83
13.3 COMPETENT AUTHORITY ... 83
13.4 INDEPENDENT ETHICS COMMITTEE ... 83
13.5 AMENDMENTS ... 84
13.6 STUDY REPORTS ... 84
13.7 INFORMED CONSENT ... 84
13.8 SUBJECT CONFIDENTIALITY ... 85
13.9 STUDY DOCUMENTATION AND ARCHIVE ... 86
13.10 COMPENSATION ... 86

14. TRIAL SPONSORSHIP AND FINANCING ... 86

15. TRIAL INSURANCE ... 87

16. TRIAL REGISTRATION ... 87

17. PUBLICATION POLICY ... 87

Table of Appendices

Appendix A: Bibliography
Appendix B: ECOG Performance Status
Appendix C: CTCAE v 4.03
Appendix D: RECIST v1.1
Appendix E: Translational research working instructions

Synopsis

**Title**	Modified FOLFOX plus/minus Nivolumab and Ipilimumab vs. FLOT plus Nivolumab in patients with previously untreated advanced or metastatic adenocarcinoma of the stomach or gastroesophageal junction - A randomized phase 2 trial.
**Design**	Randomized, open labelled, multicenter phase II trial followed by a non-randomized arm
**Indication**	Patients with Her2 negative, previously untreated metastatic esophagogastric adenocarcinoma
**Sample Size**	257 patients to be included
**Study Duration**	The total duration of recruitment is estimated to be 45 months. Follow-up time is 12 months, starting from last patient in. Six additional months are needed for analysis.
**Endpoints**	Primary endpoint:<br>Progression Free Survival acc. to RECIST v1.1 based on the ITT population for patients treated with mFOLFOX plus Nivolumab plus Ipilimumab (Arm A) vs. patients treated with mFOLFOX alone (Arm B) and progression-free survival rate (PFS@6) for Arms A2 and C.<br><br>Secondary endpoints:<br>• Progression Free Survival acc. to RECIST v1.1 for Arms A1, A2 and C<br>• Progression Free Survival rate at 6 months (PFS@6) for Arms A and B<br>• Response Rate (ORR) according to RECIST v1.1<br>• Duration of response and disease stabilization<br>• Overall survival (OS)<br>• Subgroup analysis including PFS and OS by PD-L1 expression status<br>• Safety (according to NCI-CTCAE V 4.03) and tolerability<br>• Quality of life (EORTC QLQ-C30). The QoL analyses will include QoL mean values, QoL response and time to symptom deterioration (TTSD)<br>• Translational research: correlation of biomarkers potentially associated with clinical efficacy (OS, PFS and ORR) from

	nivolumab plus/minus ipilimumab by molecular quantitation of target gene expression and immune cell composition (e.g. CTLA4, PD1, PDL1, PDL2, CD80, CD4, CD8, CD68, IGKC, CXCL9, CXCL10, CXCL13) within the tumor microenvironment and exploratory analyses based on known histological subtypes (e.g. diffuse vs. intestinal or G1/2 vs. G3) and immune related features (EBV status, MSI status, mutational load).

Trial Overview Arm A vs. B

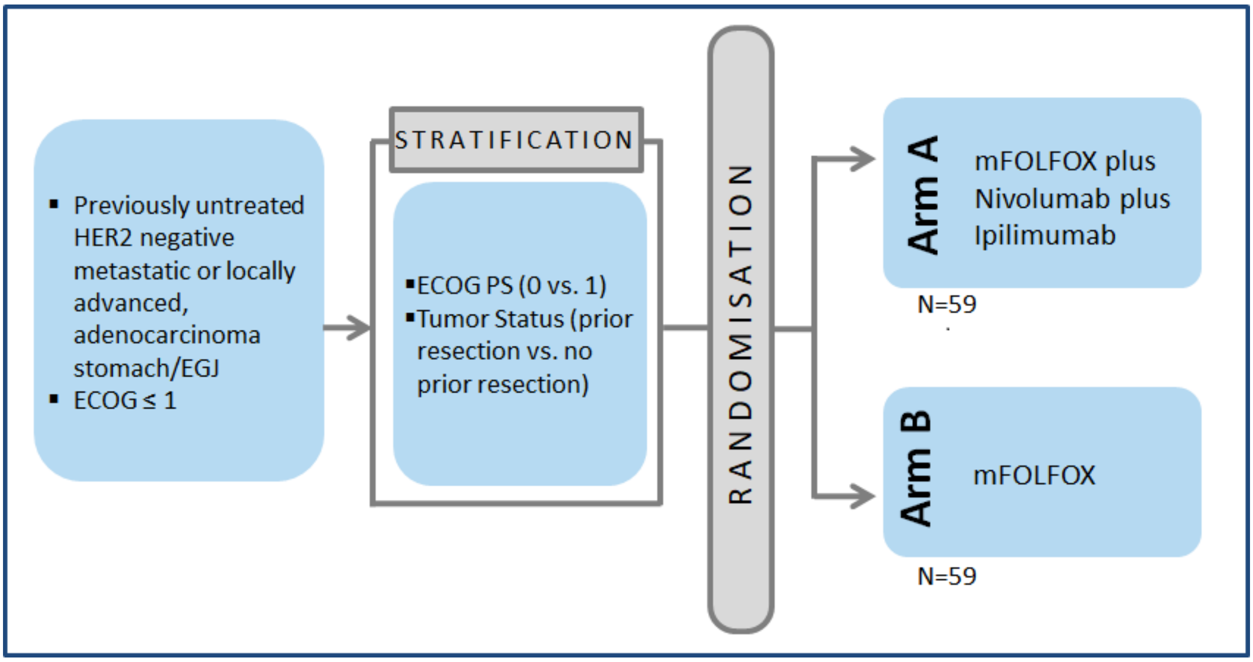

Trial Overview Arm A1 vs. A2 (Sequential Cohort)*

*Note: recruitment of Arm A and B as shown in overview above already completed at start of recruitment into Arm A1 and A2.

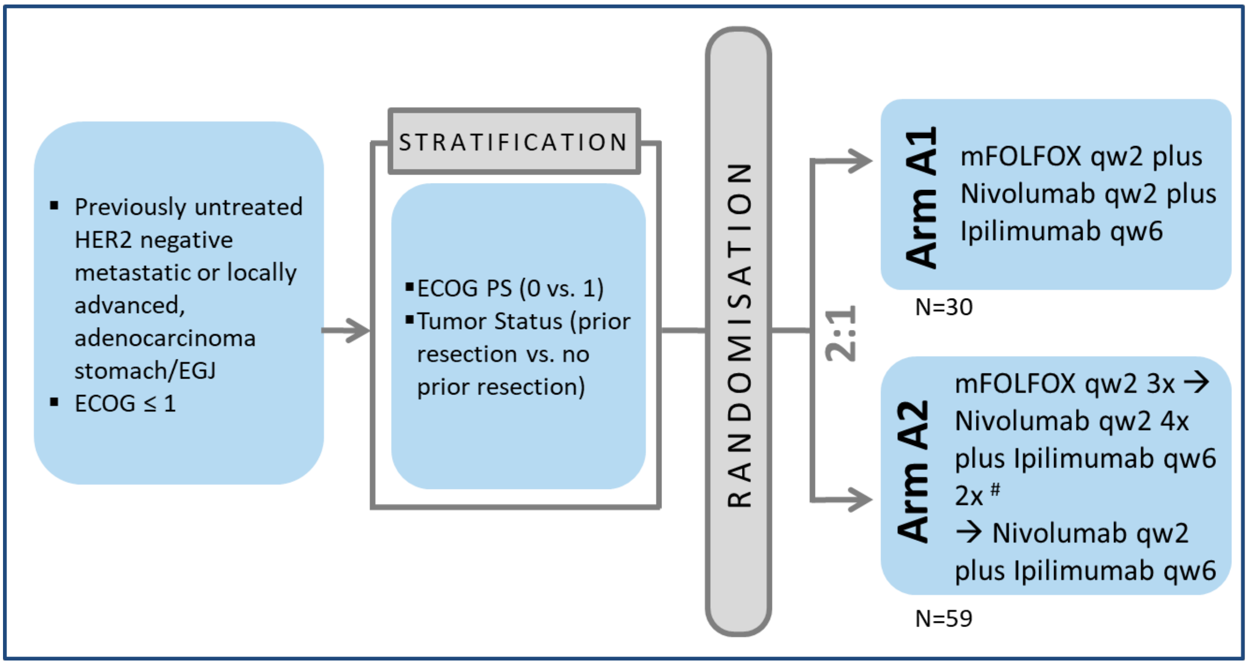

in Arm A2, upon investigator´s decision the sequence consisting of mFOLFOX 3x followed by Nivolumab 4x plus Ipilimumab 2x may be repeated as long as medically reasonable. After discontinuation of chemotherapy, immunotherapy will be continued with Nivolumab every 2 weeks and Ipilimumab every 6 weeks.

Trial Overview Arm C

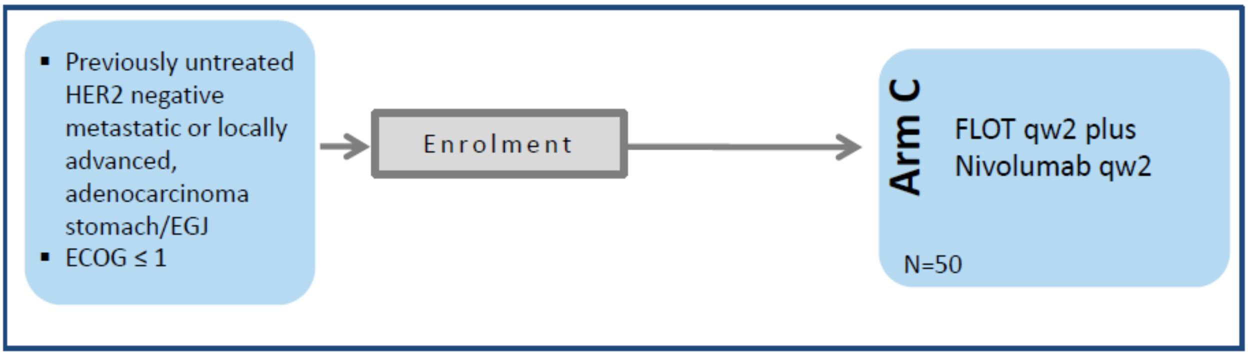

**Inclusion Criteria**	1. All subjects must have inoperable, advanced or metastatic GC or GEJ adenocarcinoma.<br>2. Subjects must have HER2-negative disease defined as either IHC 0 or I+ or IHC 2+, the latter in combination with ISH-, as assessed locally on a primary or metastatic tumour.<br>3. Subject must be previously untreated with systemic treatment given as primary therapy for advanced or metastatic disease.

	4. Prior adjuvant or neoadjuvant chemotherapy, radiotherapy and/or chemoradiotherapy are permitted as long as the last administration of the last regimen (whichever was given last) occurred at least 6 months prior to randomization/enrolment.<br>5. Palliative radiotherapy is allowed and must be completed 2 weeks prior to randomization/enrolment.<br>6. Subjects must have measurable or evaluable non-measurable disease as assessed by the investigator, according to RECIST v1.1 (Appendix D).<br>7. ECOG performance status score of 0 or 1 (Appendix B).<br>8. Life expectancy > 12 weeks<br>9. Screening laboratory values must meet the following criteria (using NCI CTCAE v.4.03):<br>a. WBC ≥ 2000/uL<br>b. Neutrophils ≥ 1500/µL<br>c. Platelets ≥ $100x10^3$/µL<br>d. Hemoglobin ≥ 9.0 g/dL<br>e. Serum creatinine ≤ 1.5 x ULN<br>f. AST ≤ 3.0 x ULN (or ≤ 5.0X ULN if liver metastates are present)<br>g. ALT ≤ 3.0 x ULN (or ≤ 5.0X ULN if liver metastates are present)<br>h. Total Bilirubin ≤ 1.5 x ULN (except subjects with Gilbert Syndrome who must have a total bilirubin level of < 3.0 x ULN)<br>10. Males and Females* ≥ 18 years of age<br>* There are no data that indicate special gender distribution. Therefore patients will be enrolled in the study gender-independently.<br>11. Subjects must have signed and dated an IRB/IEC approved written informed consent form in accordance with regulatory and institutional guidelines. This must be obtained before the performance of any protocol-related procedures that are not part of normal subject care.<br>12. Subjects must be willing and able to comply with scheduled visits, treatment schedule, laboratory tests and other requirements of the study.<br>13. Women of childbearing potential (WOCBP) must have a negative serum or urine pregnancy test (minimum sensitivity 25 IU/L or

	equivalent units of HCG) within 24 hours prior to the start of study drug. Women must not be breastfeeding.<br>14. WOCBP must agree to follow instructions for method(s) of contraception for a period of 30 days (duration of ovulatory cycle) plus the time required for the investigational drug to undergo 5 half-lives. The terminal half-lives of nivolumab and ipilimumab are approximately 25 days and 15 days, respectively. WOCBP should use an adequate method to avoid pregnancy for approximately 5 months (30 days plus the time required for nivolumab to undergo 5 half-lives) after the last dose of investigational drug.<br>15. Males who are sexually active with WOCBP must agree to follow instructions for method(s) of contraception for a period of 90 days (duration of sperm turnover) plus the time required for the investigational drug to undergo 5 half-lives. The terminal half-lives of nivolumab and ipilimumab are approximately 25 days and 15 days, respectively. Males who are sexually active with WOCBP must continue contraception for approximately 7 months (90 days plus the time required for nivolumab to undergo 5 half-lives) after the last dose of investigational drug. In addition, male subjects must be willing to refrain from sperm donation during this time.
**Exclusion Criteria**	1. Malignancies other than disease under study within 5 years prior to inclusion, with the exception of those with a negligible risk of metastasis or death (e.g., expected 5-year OS > 90%) treated with expected curative outcome (such as adequately treated carcinoma in situ of the cervix, basal or squamous cell skin cancer, localized prostate cancer treated surgically with curative intent, ductal carcinoma in situ treated surgically with curative intent)<br>2. Subjects with untreated symptomatic CNS metastases. Subjects are eligible if CNS metastases are asymptomatic (this includes patients with unknown CNS metastatic status who have no clinical signs of CNS metastases) or those with asymptomatic or symptomatic CNS who are adequately treated and are neurologically returned to baseline (except for residual signs or symptoms related to the CNS treatment) for at least 2 weeks prior to randomization/enrolment. In addition, subjects must be either off corticosteroids, or on a stable or decreasing dose of < 10 mg daily prednisone (or equivalent) for at least 2 weeks prior to randomization/enrolment. Patients with unknown CNS metastatic status and any clinical signs indicative of CNS metastases are eligible if CNS metastases are excluded using CT and/or MRI

	scans, or CNS metastases are confirmed but adequately treated as described above.<br>3. Subjects with active, known, or suspected autoimmune disease. Subjects with Type I diabetes mellitus, residual hypothyroidism due to autoimmune thyroiditis only requiring hormone replacement, or skin disorders (such as vitiligo, psoriasis, or alopecia) not requiring systemic treatment are permitted to enroll. For any cases of uncertainty, it is recommended that the medical monitor be consulted prior to signing informed consent.<br>4. Subjects with a condition requiring systemic treatment with either corticosteroids (> 10 mg daily prednisone equivalents) or other immunosuppressive medications within 14 days of study drug administration. Inhaled or topical steroids, and adrenal replacement doses > 10 mg daily prednisone equivalents are permitted in the absence of active autoimmune disease.<br>5. Prior treatment with an anti-PD-1, anti-PD-L1, anti-PD-L2, anti-CD137, or anti-CTLA-4 antibody, or any other antibody or drug specifically targeting T-cell co-stimulation or checkpoint pathways.<br>6. All toxicities attributed to prior anti-cancer therapy other than hearing loss, alopecia and fatigue must have resolved to Grade 1 (NCI CTCAE version 4.03) or baseline before administration of study drug.<br>7. > Grade 1 peripheral neuropathy according to CTCAE version4.03<br>8. Known Dihydropyrimidine dehydrogenase (DPD) deficiency<br>9. Any serious or uncontrolled medical disorder or active infection that, in the opinion of the investigator, may increase the risk associated with study participation, study drug administration, or would impair the ability of the subject to receive study drug.<br>10. Ascites which cannot be controlled with appropriate interventions.<br>11. Unstable cardiac disease despite treatment, myocardial infarction within 6 months prior to study entry; congestive heart failure NYHA grade 3 and 4<br>12. Significant acute or chronic infections including, among others:<br>a. Positive test for human immunodeficiency virus (HIV) or known acquired immunodeficiency syndrome (AIDS).<br>b. Any positive test result for hepatitis B virus or hepatitis C virus indicating acute or chronic infection.

	13. History of allergy or hypersensitivity to study drugs or any constituent of the products<br>14. Patient who has been incarcerated or involuntarily institutionalized by court order or by the authorities § 40 Abs. 1 S. 3 Nr. 4 AMG.<br>15. Patients who are unable to consent because they do not understand the nature, significance and implications of the clinical trial and therefore cannot form a rational intention in the light of the facts [§ 40 Abs. 1 S. 3 Nr. 3a AMG].
**Treatment, Dosage and Administration**	All eligible patients will be randomized in a 1:1 ratio into Arm A and B for the first 118 patients in a 1:2 ratio into Arm A1 and A2 in the subsequent 89 patients, stratified for ECOG PS (0 vs. 1) and tumor status (prior resection vs. no prior resection). Arm C (FLOT cohort) will be single arm including subsequent 50 patients.<br>**Arm A/A1***<br><br>**Nivolumab** 240mg “Flatdose” i.v. d1 every 2 weeks until disease progression or inacceptable toxicity<br><br>followed by<br><br>**Ipilimumab** 1mg/kg i.v. d1 every 6 weeks until disease progression or inacceptable toxicity<br><br>followed by<br><br>**FOLFOX**: Oxaliplatin 85 mg/m², leucovorin 400 mg/m^2 and fluorouracil 400 mg/m² administered IV on Day 1 followed by fluorouracil 2400 mg/m² IV continuous infusion over 44 hours of each treatment cycle. Cycles are repeated every 2 weeks until disease progression or inacceptable toxicity or end of study treatment. Chemotherapy can also be administered per local standard.<br><br>Therapy can also be splitted, administering nivolumab/ipilimumab on day one and FOLFOX starting at day two of the cycle.<br><br>**Arm A2**<br><br>**Three cycles of induction chemotherapy with FOLFOX**:<br>Oxaliplatin 85 mg/m², leucovorin 400 mg/m^2 and fluorouracil 400 mg/m² administered IV on Day 1 followed by fluorouracil 2400 mg/m² IV continuous infusion over 44 hours of each treatment cycle. Cycles are

	repeated every 2 weeks. Chemotherapy can also be administered per local standard.<br><br>**Followed by immunotherapy** consisting of 4 administrations of Nivolumab at 240mg “Flatdose” i.v. d1 every 2 weeks<br>and 2 administrations of Ipilimumab at 1mg/kg i.v. d1 every 6 weeks<br><br>**Repetition of chemotherapy and immunotherapy:**<br>The above described therapy sequence consting of 3 cycles of FOLFOX followed by immunotherapy may be repeated starting two weeks after last administration of immunotherapy once, or, if medically reasonable, for an unlimited number of repetitions upon investigator decision. However, repetition of chemotherapy after the first 3 cycles is optional and may be skipped.<br>After completion or discontinuation of chemotherapy, immunotherapy will be continued consisting of:<br>Nivolumab at 240mg “Flatdose” i.v. d1 every 2 weeks<br>and Ipilimumab at 1mg/kg i.v. d1 every 6 weeks<br><br>Study therapy as described above will be administered until disease progression, inacceptable toxicity, patient’s request, or end of study treatment phase (24 months).<br><br>**<u>Arm B</u>**<br><br>**FOLFOX**: Oxaliplatin 85 mg/m², leucovorin 400 mg/m² and fluorouracil 400 mg/m² administered IV on Day 1 followed by fluorouracil 2400 mg/m² IV continuous infusion over 44 hours of each treatment cycle. Cycles are repeated every 2 weeks until disease progression or inacceptable toxicity or end of study treatment. Chemotherapy can also be administered per local standard.<br><br>Note: there will be an interim safety analysis comprising the first 15 patients enrolled into the experimental study arm and treated for at least 6 weeks. Safety data of the first 15 patients in Arm A and the corresponding patients in Arm B will be analysed based on a comprehensive analysis plan and reviewed by the lead investigators and by the IDMC. Recruitment will not be stopped during this analysis.

	**Arm C**** <br>**Nivolumab** 240mg "Flatdose" i.v. d1 every 2 weeks until disease progression or inacceptable toxicity <br>Followed by <br>**FLOT:** Docetaxel 50mg/^{2}, Oxaliplatin 85 mg/m², leucovorin 200 mg/m^2 on day 1 and fluorouracil 2600 mg/m² IV continuous infusion over 24 hours of each treatment cycle. <br>Cycles are repeated every 2 weeks until disease progression or inacceptable toxicity or end of study treatment. After completion or discontinuation of chemotherapy, immunotherapy may be continued consisting of: <br>Nivolumab at 240mg "Flatdose" i.v. d1 every 2 weeks <br>Chemotherapy can also be administered per local standard. <br>Therapy can also be splitted, administering nivolumab on day one and FLOT starting at day two of the cycle. <br>**Duration of treatment** <br>Treatment with each of the components FOLFOX, FLOT, nivolumab and/or ipilimumab will be administered until progression (according to RECIST v1.1), intolerable toxicity, patient's request, or end of study treatment phase (24 months). The study treatment will be limited to a maximum of 24 months. If one component of the treatment is stopped for any cause, the other components can be continued. <br>*Arm A1 is identical to Arm A. Patients are randomized concurrently with Arm A2 after recruitment of the first 118 patients into Arm A and B is completed. A1 will comprise 30 patients. <br>** Arm C will recruit 50 patients
**Assessments**	**Baseline (within 4 weeks before treatment start)** <br>• Review of inclusion and exclusion criteria • Medical and medication history, physical examination including height, weight, ECOG-performance status and concomitant medication • Laboratory Tests: Hematology panel: hemoglobin, platelets, WBC with neutrophils, lymphocytes, monocytes, eosinophils, and basophils)

	Chemistry panel: sodium, potassium, calcium, magnesium, serum creatinine, urea, alkaline phosphatase, AST, ALT, total and direct bilirubin, glucose, lipase, amylase, LDH<br>Free T3/T4 and TSH<br>Coagulation: INR, aPTT<br>CEA, CA 19-9<br>Hepatitis B/C screening test (HBsAg, anti-HBc, anti-HBs, anti-HCV)<br>HIV testing within 7 days prior to start of the treatment<br>• Pregnancy test for women of childbearing potential within 24 hours prior to start of the treatment<br>• Blood draw for translational research<br>• Obtain paraffin-embedded tumor-tissue for PD-L1 assessment and translational research<br>• ECG<br>• Quality of life assessment (EORTC QLQ-C30)<br>• Disease assessment by radiological imaging of the chest, abdomen, pelvis and all other sites of disease (CT/MRI-scan). Already available investigations can be used for the study if within the time frame.<br><br>**During treatment** (safety-relevant assessments, including pregnancy test have to be completed before dosing)<br>• Physical examination, performance status (ECOG), assessment of toxicity<br>• Laboratory tests (hematology and chemistry panel), including<br>• Free T3/T4 and TSH (after every 4th 2-week cycle= approximately every 8 weeks)<br>• Pregnancy test for women of childbearing potential (after every 4th 2-week cycle= approximately every 8 weeks)<br>• Quality of life assessment (EORTC QLQ-C30) after every 4th cycle (= approximately every 8 weeks (prior to imaging)<br>• CEA, CA 19-9 (only every 8 weeks, together with imaging)<br>• Disease assessment (tumor response assessment) every 8 weeks

Final staging

When any subject discontinues dosing of all study treatment, the following assessments should be made:

- Physical examination, performance status (ECOG), assessment of toxicity
- Laboratory tests (baseline panel), including free T3/T4 and TSH and pregnancy test for women of childbearing potential
- ECG
- Disease assessment

Extended safety follow-up: **30 and 100 days safety follow-up (±7 days)**

- Physical examination, performance status (ECOG), assessment of toxicity
- Laboratory tests (hematology and chemistry panel), including free T3/T4 and TSH and pregnancy test for women of childbearing potential

Given the potential risk for delayed immune-related or other toxicities, safety follow-up must be performed at 30 and 100 days after the last dose of study therapy (all arms).

The extended safety follow-up beyond 30 days after last study drug administration may be performed either via a site visit or via a telephone call with subsequent site visit requested in case any concerns noted during the telephone call.

Individual Follow-up

All subjects will be followed every 3 months ± 28 days for up to 4 years after start of recruitment (this is the individual follow up, which differs from the overall minimum follow up determined by the statistical calculation and which is 3 years consisting of 2 years recruitment and 1 year follow up after last patient-in).

In case of progressive disease after study treatment only:

- Survival, disease status, protracted toxicity, further treatment

In any other case additionally:

- Disease assessment, physical examination including weight, ECOG-performance status

<table>
<tr><td></td><td>Tumor Response Assessment
During treatment, tumor response will be assessed by the investigator according to RECIST v1.1 (Radiological imaging by CT and/or MRI of the chest, abdomen, pelvis and all other sites of disease) after every 4th cycle, which represents the standard of care in Germany. After treatment discontinuation for reasons other than progressive disease imaging will be performed every 3 months (±7 days) until progression or death, which represents the standard of care in Germany.

Safety
Safety assessments will include physical examinations, performance status (ECOG), clinical laboratory profile and adverse events.
All observed toxicities and side effects will be graded according to NCI CTCAE v4.03 for all patients and the degree of association of each with the procedure assessed and summarized.
Treatment related serious adverse events rate (SAE) will be determined.

Quality of Life
Quality of life will be assessed using the EORTC QLQ-C30 together with tumor response assessment</td></tr>
<tr><td>Translational Research</td><td>The following translational research is currently planned, but may be adapted taking into account new research data:
FFPE tissue will be centrally tested for target gene expression and immune cell composition by molecular quantitation of PD-L1, PD-L2, PD1, CTLA4, CD80, CD3, CD4, CD8, FOXP3, CD68, IGKC, CXCL9, CXCL10, CXCL13 and correlated with clinical efficacy.
In addition FFPE tissue will be centrally tested for MSI, EBV, ERBB2, MMP7, MMP9, RTK signalling pathway members and mutation status (kRAS, TP53).
Thus, the tumor block or slices will be obtained at baseline. EDTA blood will be collected once.</td></tr>
<tr><td>Statistical Considerations</td><td>Arm A vs. B:
PFS analysed according to the ITT principle for patients treated with mFOLFOX plus Nivolumab plus Ipilimumab (Arm A) vs. patients treated with mFOLFOX alone (Arm B) is the primary efficacy endpoint.
The expected median PFS in the standard arm is 5.5 months; the expected median PFS in the experimental arm is 8.5 months. We hypothesize that</td></tr>
</table>

the experimental therapy is associated with clinically relevant improvement according to a HR of 0.68. In the frame of a phase II testing, the use of a one-sided significance level of 10% is justified. Based on this, 118 randomized subjects (59 in the control and 59 in the experimental treatment group) will be enrolled to provide 80% power for detecting an average HR of 0.68 using the log rank test at a one-sided type I error of 10% and assuming a 5% drop out rate. The sample size calculation is based on 2 years recruitment time and 1 year follow up time after last patient-in. So the minimum follow-up time is 3 years.

1:1 Randomization will be performed according to the following stratification criteria:

- ECOG PS (0 vs 1)
- Tumor status (prior resection vs. no prior resection)

Arm A1 vs. A2:

To evaluate if a sequential treatment of mFOLFOX plus Nivolumab plus Ipilimumab (Arm A2) is less toxic but equally effective as parallel treatment of mFOLFOX plus Nivolumab plus Ipilimumab (Arm A and A1) 57 patients are needed using a one-stage Fleming design (Fleming 1982) with following assumptions:

- The sequential therapy would be rated as unacceptable, if the actual PFS rate at 6 months (PFS@6) was only 47% or lower (corresponding to the median PFS of Arm B of 5.5 months)
- The sequential therapy would be considered to be a promising candidate for further development, if the true PFS@6 amounted to 61% or higher (corresponding to the expected median PFS of Arm A of 8.5 months)
- Probability to accept the sequential therapy as effective, in spite of a true PFS@6 of <47%: 10% (type I error)
- Probability to reject the sequential therapy as ineffective (<47%), although the true PFS@6 is promising (>61%): 20% (type II error, corresponding to a power of 80%)

Allowing for two non-informative drop-outs, 59 patients have to be recruited into Arm A2. 30 patients are to be allocated to the reference arm A1, according to the 1:2 randomization. The same stratification factors (ECOG PS and tumor status) as in the randomization of arms A and B are applied.
The final conclusion for the sequential treatment will depend on the definite PFS rate and its confidence interval, the respective findings in the reference arm, as well as the information on type, frequency and severity of toxicities.

	**Arm C:**<br>Based on emerging data from recent phase III trials, immune checkpoint inhibitors such as Nivolumab and Pembrolizumab added to the doublet chemotherapy prolong OS and PFS, but the extent of improvement regarding PFS is smaller than expected and if administered as monotherapy, there is a an increased early mortality (crossing survival curves) with the checkpoint inhibitors as compared with chemotherapy (Tabernero et al. 2019; Janjigian et al. 2021; Kato et al. 2020). This is most likely explained by the fact that patients need time to establish antitumoral immunity, while some patients with aggressive disease experience early disease progression and death. This provides a rationale to test whether the intensification of chemotherapy using a triplet (mFOLFOX plus Docetaxel = FLOT) instead of the doublet, reducing immunotherapy to Nivolumab instead of Nivolumab and Ipilimumab would be more beneficial and safer. Therefore, Arm C is designed to evaluate the efficacy of the combination of FLOT plus Nivolumab in the same patient group and descriptively compare this treatment with mFOLFOX plus Nivolumab plus Ipilimumab (Arm A) and with mFOLFOX alone (Arm B). The sample size is chosen according to clinical reasoning. No statistical hypothesis testing is planned. A sample size of 50 patients to be treated with FLOT plus Nivolumab is regarded sufficient to get a first sight into the efficacy and to obtain data on the feasibility, safety and toxicity of the the study treatment.<br>As our study generally aims at gaining insights into the potentially most optimal chemo-immunotherapy regimen (therapy optimization) for a future trial, the implementation of Arm C fits well into the concept of the trial. With Arm C our trial evaluates three variations for the concept of immunochemotherapy: chemotherapy doublet plus immunotherapy doublet administered in parallel, chemotherapy doublet plus immunotherapy doublet administered sequentially, and chemotherapy triplet plus immune monotherapy administered in parallel.<br>Because Arms A and B are fully recruited, it is not possible to perform a randomized comparison for Arm C. However, using the patients from Arms A and B as a comparator is still better than historical controls as these patients are treated by the same centres, under the same therapeutic and diagnostic guidelines in a close time period.<br>The KEYNOTE-062 study (Tabernero et al. 2019) reported a median PFS of 6.9 months for the combination of Pembrolizumab plus chemotherapy while the Checkmate 649 (Janjigian et al. 2021) reported a median PFS of 7.7 months for the combination of Nivolumab plus mFOLFOX. Therefore, we expect the true median PFS of FLOT plus Nivolumab to be between 8 and 9 months corresponding to PFS rates at 6 months of about 59% and 63%.<br>The final conclusion for the combination of FLOT plus Nivolumab will depend on the definite PFS rate at 6 months (and its confidence interval),

<table>
<tr><td></td><td>the respective findings in the arms A and B, as well as the information on type, frequency and severity of toxicities. The precision of the estimation of the PFS@6 is provided by confidence intervals (CIs) in the following table, for different actual PFS@6 findings:

<table><tr><th>PFS@6</th><th>Exact 95% CI</th></tr><tr><td>29/50 (58%)</td><td>43.2% … 71.8%</td></tr><tr><td>30/50 (60%)</td><td>45.2% … 73.6%</td></tr><tr><td>31/50 (62%)</td><td>47.2% … 75.4%</td></tr><tr><td>32/50 (64%)</td><td>49.2% … 77.1%</td></tr></table></td></tr>
<tr><td>Interim Safety analysis</td><td>In this trial, there will be an interim safety analysis conducted when the first 15 patients have been enrolled into the experimental study arm and treated for at least 6 weeks. Safety data of the first 15 patients in Arm A and the corresponding patients in Arm B will be analysed based on a comprehensive analysis plan and reviewed by the lead investigators (Prof. Götze and Prof. Lorenzen) and by the IDMC. Recruitment will not be stopped during this analysis.In case the recruitment is stopped prematurely for toxicity after the safety analysis, there will be a meeting of the steering committee to decide on further steps. The steering committee can decide to permanently discontinue the trial or to amend the protocol.</td></tr>
</table>

Flow chart

Study Schedule Visit	Inclusion	During treatment	End of Treatment[6]	Extended Safety follow up	Individual Follow up
**Study week (wks)**	Baseline (within 4 wks prior to cycle 1)	Cycle 1 day1, afterwards every 2 wks (+3/-2 days)		After 30 and 100 days [7] (±7 days)	every 3 months (±28 days)
**Informed consent**	X				
**Medical and medication history, Her2 status, demographics**	X				
**Obtain tumor tissue for translat. research**	X[2]				
**Blood draw (2 EDTA tubes a 2 mL) for translat. research**	X[2]				
**HIV testing[8]**	X				
**Physical examination[1]**	X	X	X	X	X[6]
**Performance status (ECOG)**	X	X	X	X	X
**Cardiac evaluation: ECG**	X		X		
**Laboratory [3]**	X	X	X	X	
**Tumor markers (CEA, CA 19-9)**	X	X[4]	X		X[5]
**Quality of life assessment (EORTC QLQ-C30)**	X	X[4]	X		X[5]
**Tumor assessment (CT/MRI)**	X	X[4]	X		X[5]
**Further treatment**					X
**AE monitoring**		X			
**Survival**		X			

1: including height (only baseline), weight
2: blood draw (2 EDTA tubes a 2 mL) will be sent to Dr. Ralph Wirtz (STRATIFYER) and tumor tissue (centers may send either paraffin block or slices; 1 x HE stained slice, 4 x 5 µm unstained tissue slices on glass slides) will be sent to IKF
3: hematology panel (hemoglobin, platelets, WBC with neutrophils, lymphocytes, monocytes, eosinophils, and basophils), chemistry panel (sodium, potassium, calcium, magnesium, serum creatinine, urea, alkaline phosphatase, AST, ALT, total and direct bilirubin, glucose, lipase, amylase and LDH screening at baseline and on day 1 prior every treatment cycle, coagulation (INR, aPTT), and hepatitis b/c screening only baseline, serum pregnancy test in women of childbearing potential at baseline (24 hours prior to start of the treatment) and after every 4th 2-week cycle = approximately every 8 weeks, free T3/T4 and TSH at baseline and after every 4th 2-week cycle = approximately every 8 weeks.
4: approx. every 8 weeks (±7 days) until EOT, afterwards every 3 months
5: only in case of no progressive disease during or after 1st line treatment
6: If results of examinations are available which are not older than 14 days (tumor assessment and QoL: not older than 6 weeks) no further examination necessary.
7: 30d safety follow up should be on site; the extended safety follow-up beyond 30 days after last study drug administration may be performed either via a site visit or via a telephone call with subsequent site visit requested in case any concerns noted during the telephone call.
8: Mandatory within 7 days prior to the start of therapy.

Study Schema Arm A vs. B

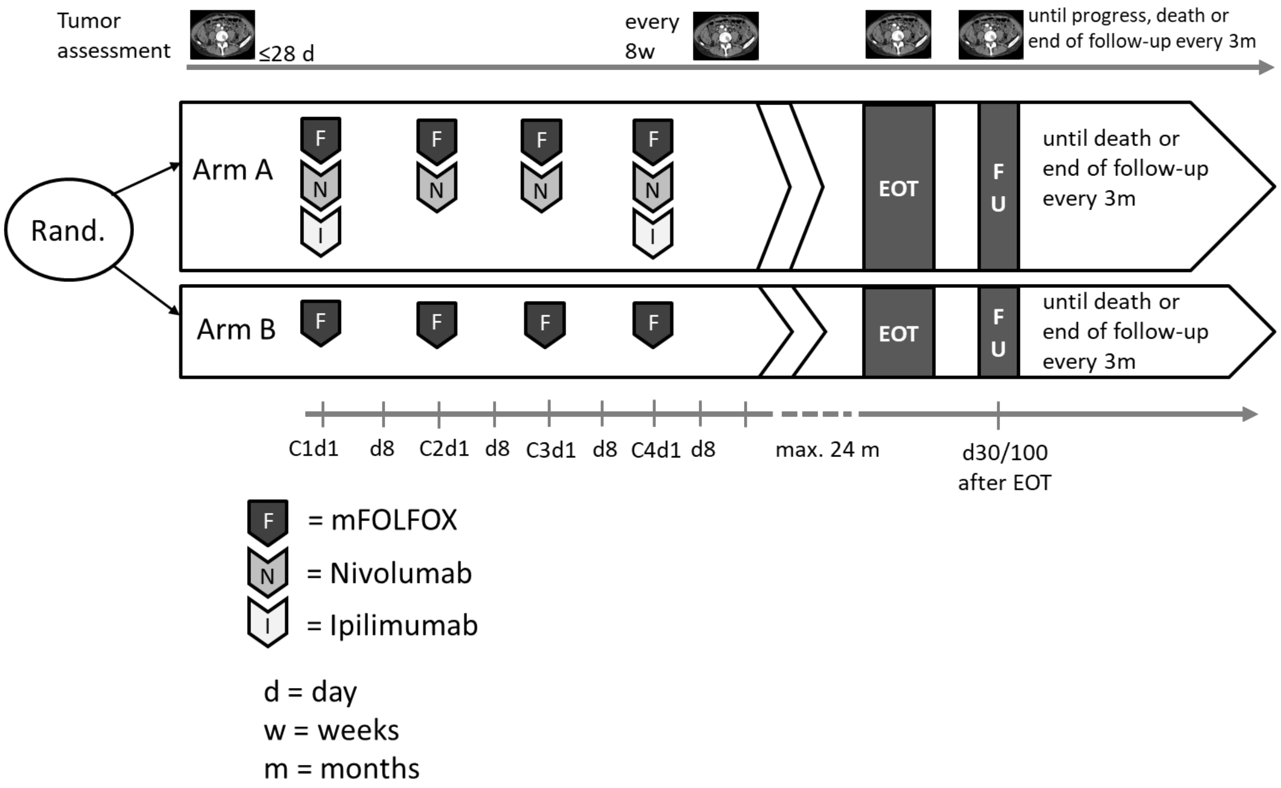

Study Schema Arm A1 vs. A2

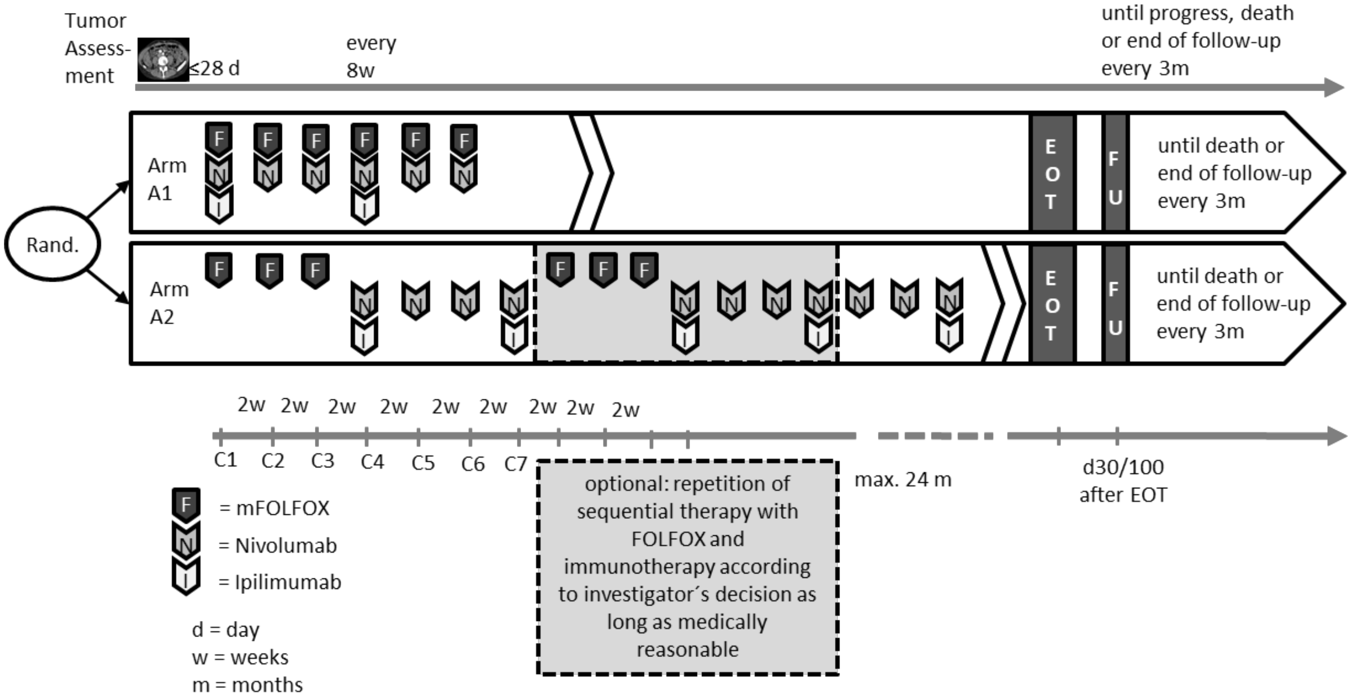

Study Schema Arm C

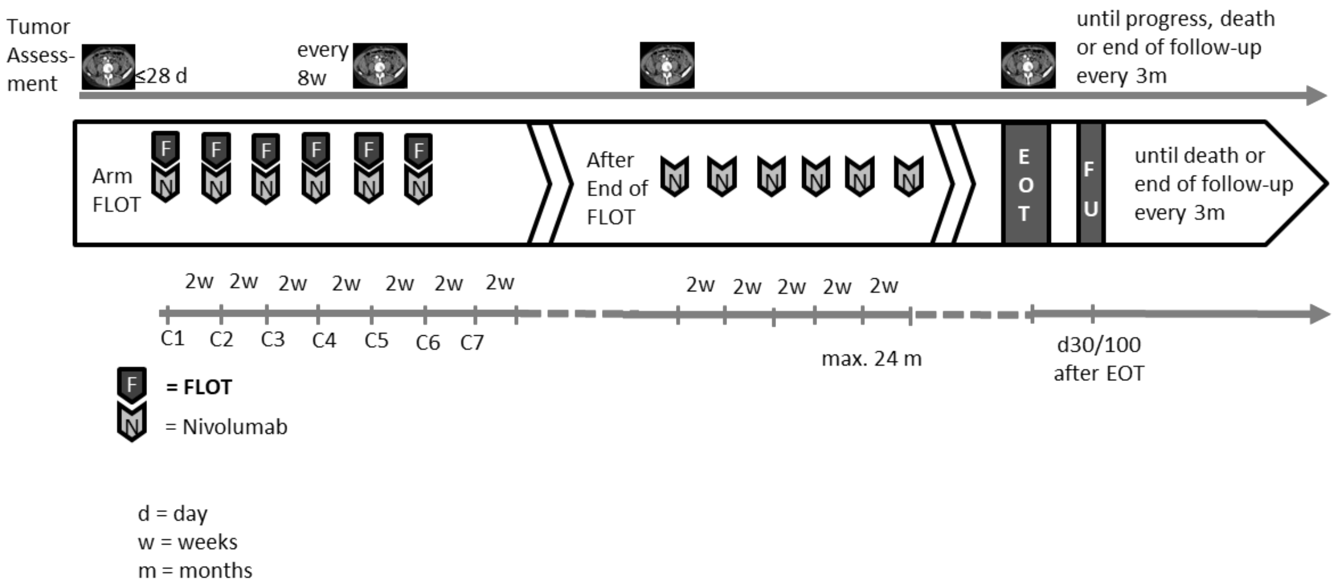

1. Introduction and Background

1.1 Epidemiology and Disease Background

Gastric cancer is the fifth most common cancer in the world, with an estimated 951,000 new cases diagnosed in 2012 (6.8% of total cancer cases), and the third leading cause of cancer death in both sexes worldwide, with 723,000 deaths (8.8% of total cancer deaths) estimated in that year (Ferlay, Soerjomataram et al. 2015). In Europe, 139,600 new cases were diagnosed and 107,300 patients died of gastric cancer in 2012 (Ferlay, Steliarova-Foucher et al. 2013). Gastric cancer is more frequent among males and ist incidence increases with age, peaking between 65 and 74 years of age (Ferlay, Soerjomataram et al. 2015). The incidence of tumors located in the gastric cardia and gastroesophageal junction has increased in past decades and is linked to risk factors such as obesity and gastroesophageal reflux disease (Buas and Vaughan 2013).

Surgical resection is currently the only curative treatment option for gastric cancer; however, ~50% of patients have metastatic disease at the time of diagnosis and chemotherapy is the mainstay of palliation in this setting (Smyth, Verheij et al. 2016). Best supportive care (BSC) plus chemotherapy has been shown to be more effective than BSC alone in patients with advanced gastric cancer, with combination chemotherapy more effective than single-agent treatment (Wagner, Unverzagt et al. 2010).

Patients with unresectable or metastatic esophagogastric adenocarcinoma are candidates for chemotherapy-based palliative treatment; with a doublet of a platinum compound and a fluoropyrimidine currently regarded as an acceptable standard first-line option (Smyth, Verheij et al. 2016). Platinum compounds (oxaliplatin and cisplatin) and fluoropyrimidines (5-fluorouracil, capecitabine, and tegafur/gimeracil/oteracil potassium [S1]) are generally considered as first-line

(1L), standard-of-care treatment options in metastatic GC and GEJ cancer across geographic regions (NCCN guidelines 2016; Waddell and Verheij 2013; Japanese Gastric Cancer treatment guidelines 2010). Since the REAL-2 study demonstrated an oxaliplatin-based regimen to be non-inferior to cisplatin with a favorable safety profile (Cunningham and Starling 2008), oxaliplatin combinations with fluoropyrimidines have been studied in multiple Phase 2 and 3 trials, and showed similar efficacy trends across regions (Yamada and Higuchi 2015; Al-Batran and Hartmann 2008).
A Phase 3 trial in esophageal/gastric/GEJ cancers comparing the FLO (mFOLFOX) regimen (5-fluorouracil plus leucovorin and oxaliplatin) vs FLP (5-fluorouracil plus leucovorin and cisplatin) showed no statistically significant differences between the 2 treatments, but favored the FOLFOX arm vs the FLP arm in terms of median PFS (the primary endpoint, 5.7 months vs. 3.9 months), response rate (35% versus 25%), and median survival (10.7 months vs 8.8 months) (Al-Batran and Hartmann 2008). As a result, oxaliplatin has become one of the major backbone platinum compounds in the 1L setting and is a generally accepted an active comparator in Phase 2 or Phase 3 randomized studies by health authorities worldwide. Based on these observations, the oxaliplatin-based regimens FOLFOX is considered to be reasonable comparators in this Phase 2 study.

In the past decade, multiple new investigational drugs with mainly molecular targets have been investigated in the 1L setting as add-ons to backbone platinum and fluoroprimidine treatment. However, except trastuzumab, all targeted agents approved for other cancer indications including cetuximab and bevacizumab have failed to demonstrate efficacy as single agents and in combination with chemotherapeutics.

1.2 Rationale for Checkpoint Inhibition in Esophagogastric Adenocarcinoma

Nivolumab (BMS-936558), a programmed cell death protein-1 (PD-1) antibody, with or without ipilimumab (BMS-734016), a cytotoxic T-cell lymphoma-4 (CTLA-4) antibody have demonstrated clinical efficacy in several advanced cancer types, including melanoma, non-small cell lung cancer, and renal cell carcinoma (Brahmer, Reckamp et al. 2015, Larkin, Chiarion-Sileni et al. 2015, Motzer, Escudier et al. 2015).

1.2.1 Single Agent PD-1 Inhibition

Anti PD-1 and PD-L1 inhibitors (eg, nivolumab and pembrolizumab) have been investigated in GC treatment and have demonstrated anti-tumor activity (Janjigian, Bendell et al. 2016, Muro, Chung et al. 2016).

Treatment with pembrolizumab achieved a 33% ORR by investigator assessment and 22% by central data review in GC subjects with PD-L1 expressing tumors. The 6-month progression-free survival (PFS) rate was 26% and median PFS was 1.9 months (95% CI: 1.8, 3.5). The 6-month OS rate was 66% and mOS was 11.4 months (95% CI: 5.7, NR). PD-L1 expressing tumors (cutoff 1%) were reported in 40% of GC patients in this study, which is consistent with previous reports (Muro, Chung et al. 2016).

Single agent nivolumab achieved a 15% ORR in GC subjects independent of PD-L1 expression and

up to 27/33% in patients with PD-L1 positivity in >1%/>5%, respectively. The 6-month progression-free survival (PFS) rate was 18% and median PFS was 1.9 months (95% CI: 1.8, 3.5). The 6-month OS rate was 49% and mOS was 5 months (Janjigian, Bendell et al. 2016).

1.2.2 Combination of PD-1 and CTLA-4 Receptor Blockade

Preclinical data indicate that the combination of PD-1 and CTLA-4 receptor blockade may improve antitumor activity (Curran, Montalvo et al. 2010). In vitro combinations of nivolumab plus ipilimumab have increased INF-γ production 2- to 7-fold over either agent alone in a mixed lymphocyte reaction. In a murine melanoma vaccine model, blockade with either CTLA-4 or PD-1 antibodies increased the proportion of CTLA-4- and PD-1-expressing CD4/CD8 tumor-infiltrating T-effector cells, and dual blockade increased tumor infiltration of T-effector cells and decreased intratumoral T regulatory cells, as compared to either agent alone.

In the Phase 1 dose escalation study CA209004, the combination of nivolumab and ipilimumab has been studied in subjects with unresectable or metastatic melanoma (Postow, Chesney et al. 2015). In this study, a safe dose level for the combination of ipilimumab and nivolumab was established for the treatment of advanced melanoma. At this dose level, 3 mg/kg ipilimumab plus 1 mg/kg nivolumab, an objective response rate of 53% was observed. This dose level has been approved in subjects with advanced melanoma in the US based on the Phase 3 study CA209067. In this study, the combination of nivolumab and ipilimumab has demonstrated increased benefit compared to either ipilimumab or nivolumab monotherapies in subjects with advanced melanoma (Larkin, Chiarion-Sileni et al. 2015).

In EGA, the open-label, multi-center Phase 1/2 study CA209032 investigated the safety and efficacy of nivolumab monotherapy or nivolumab plus ipilimumab combination therapy in multiple tumor types including GC/GEJ (Janjigian, Bendell et al. 2016). The following dose cohorts enrolled subjects with GC/GEJ cancer who had previously received at least one prior therapy, and more than 80% of subjects received more than 2 prior therapies:

- N3 monotherapy cohort: nivolumab 3 mg/kg IV every 2 weeks (n = 59)
- N1+I3 cohort: nivolumab 1 mg/kg + ipilimumab 3 mg/kg, IV every 3 weeks for 4 doses, followed by nivolumab 3 mg/kg IV every 2 weeks (n = 49);
- N3+I1 cohort: nivolumab 3 mg/kg + ipilimumab 1 mg/kg, IV every 3 weeks for 4 doses, followed by nivolumab 3 mg/kg IV every 2 weeks (n = 52).

The results are displayed in table 1. Based on the non-randomized design no comparative efficacy can be assessed in this trial.

Table 1: Results of Checkmate 032 gastric cancer cohort (N3: nivolumab; 3 mg/kg; I1: ipilimumab, 1mg/kg; N1: nivolumab, 1 mg/kg: I3: ipilimumab, 3 mg/kg; ORR: objective response rate; PFS: progression-free survival; mOS:median overall survival; CI: confidence interval; 1L: first line)

**Clinical Activity of Nivolumab Monotherapy, and Nivolumab-plus-Ipilimumab Combination Therapy in Subjects with GC/GEJ Cancer (CA209032)**			
	**N3 monotherapy N=59**	**N3+I1 N=52**	**N1+I3 N=49**
**Population**	**> 1L Prior therapy**		
Confirmed ORR, % (95% CI)	13.6 (6.0 - 25.0)	10.2 (3.4 - 22.2)	26.1 (14.3 - 41.1)
Duration of Response, mos. (95% CI)	7.1 (2.3 - 13.2)	NA (2.5 - NA)	5.6 (2.8 - NA)
PFS rate at 24 wks, % (95% CI)	17.7 (9.0 - 28.7)	8.8 (2.8 - 19.1)	23.9 (12.4 - 37.5)
Median PFS, mos (95% CI)	1.36 (1.25 - 1.51)	1.58 (1.38 - 2.6)	1.45 (1.25 - 3.94)
1-year OS rate, % (95% CI)	36 (21 - 51)	NA	34 (19 - 50)
Median OS, mos (95% CI)	5.03 (3.35 - 12.42)	4.83 (3.02 - 9.07)	6.87 (3.61 - NA)
Median length of follow-up, mos (range)	16.78 (1.86 - 24.79)	6.26 (1.07 - 13.28)	8.54 (0.93 - 12.40)

The N1+I3 cohort, despite being numerically more active, resulted in a high rate of grade 3/4 toxicity of 45% (in 22 of 45 patients). The N3+I1 cohort was better tolerated with a grade 3/4 toxicity of 27% (14 of 52 patients).

Thus, for further particularly combination strategies the N3+I1 was chosen. Scheduling was amended to a flat dose nivolumab (240mg) (FDA approved in Sep 2016). Administration of ipilimumab was expanded to 6weekly intervals to further improve tolerability.

1.3 Rationale for Including Both Subjects with PD-L1 and Non-PD-L1 Expressing Tumors in 1L GC/GEJ

Single-agent nivolumab in previously treated tumors, including squamous and non-squamous NSCLC and RCC, has demonstrated improved survival in subjects without preselection by PD-L1 tumor expression (Brahmer and Reckamp 2015; Larkin and Chiarion-Sileni 2015; Motzer and Escudier 2015; Borghaei and Paz-Ares and 2015). In GC/GEJ cancer, PD-L1 has been suggested as a prognostic marker (Wu and Zhu 2006; Ohigashi and Sho 2005). Clinical data for the N1+I3 combination suggests that PD-L1 may also be predictive (eg, numerically higher ORR) as a therapy targeting PD-1 alone or in combination with CTLA-4, but also having activity in subjects whose

tumors did not express PD-L1. In addition, in the CA209067 study, nivolumab combination with ipilimumab demonstrated clinical activity in subjects with advanced melanoma tumors which did not express PD-L1. For these reasons, this trial will enroll subjects regardless of PD-L1 tumor expression.

1.4 Rationale for Choice of Comparator

Platinum compounds (oxaliplatin and cisplatin) and fluoropyrimidines (fluorouracil, capecitabine, and S1) are generally considered as the standard-of-care, 1L treatment options of choice across geographic regions (NCCN guidelines 2016; Waddell and Verheij 2013; Japanese Gastric Cancer treatment guidelines 2010; Kang and Kauh 2011). The NCCN guideline suggests the 2-drug chemotherapy as the preferred regimen in the 1L setting; the 3-drug regimen, due to its toxicity profile, should be considered only for use in patients with good performance statuses (NCCN guidelines 2016). Thus the comparator selected in this study is only the platinum doublet regimen.Cisplatin has been the most frequently administered platinum in GC treatment. Since the REAL-2 study demonstrated an oxaliplatin-based regimen to be non-inferior to cisplatin with a favorable safety profile (Cunningham D, Starling 2008), oxaliplatin combinations with fluoropyrimidines have been studied in multiple Phase 2 and 3 trials, and showed similar efficacy trends across regions (Yamada and Higuchi 2015; Al-Batran and Hartmann 2008,van Meerten and Eskens 2007, Jatoi and Murphy 2006). A meta-analysis comparing oxaliplatin-based and cisplatin-based regimens showed that oxaliplatin was associated with significant improvements in PFS (HR 0.88, 95% CI: 0.80 - 0.98) and OS (HR 0.88, 95% CI: 0.78 - 0.99), with fewer AEs of neutropenia, anemia, alopecia and thromboembolic events, but more neurotoxicity and diarrhea (Montagnani and Turrisi 2011). As a result, oxaliplatin has become one of the major backbone platinum compounds in the 1L setting. A Phase 3 trial in esophageal/gastric/GEJ cancers comparing the FOLFOX regimen (5-fluorouracil plus leucovorin and oxaliplatin) vs FLP (5-fluorouracil plus leucovorin and cisplatin) showed no statistically significant differences between the 2 treatments, but favored the FOLFOX arm vs the FLP arm in terms of median PFS (the primary endpoint, 5.7 months vs 3.9 months), response rate (35% versus 25%), and median survival (10.7 months vs 8.8 months). FOLFOX was associated with significantly less nausea and vomiting, fatigue, renal toxicity and alopecia, but more Grade 3/4 sensory neuropathy than FLP (Al-Batran and Hartmann 2008). Based on these observations, the oxaliplatin-based regimen FOLFOX is considered to be reasonable comparators in this Phase 2 study.

1.5 Rationale for the Combination of Checkpoint Inhibition and Chemotherapy

Experiments have confirmed the in vivo efficacy of PD-L1 blockade as a monotherapy as well as in combination with chemotherapy in syngeneic mouse tumor models. Moreover, immunotherapy has been shown to improve the efficacy of chemotherapy (Kershaw and Devaud 2013). In chemotherapy naïve non-small cell lung cancer, the phase II KEYNOTE 012 trial demonstrated an doubled response rate (55% vs 29%; p=0.0016) when pembrolizumab was added to a cisplatinum-doublet chemotherapy with a manageable safety profile (Langer and Gadgeel 2016). It is anticipated that the

combination of standard chemotherapy with combined Nivolumab/Ipilimumab immunotherapy will increase clinical activity.

1.6 Rationale for alternating Checkpoint Inhibition with chemotherapy in Esophagogastric Adenocarcinoma

Although single-agent anti-PD-1 is clearly a therapeutic breakthrough for defined subgroups (MSI-H, probably EBV positive), the majority of patients have only limited benefit from immunmonotherapy with rather moderate tumor response rates between 10 and 15%. Currently, only few data are available for the combination of checkpoint inhibitors with chemotherapy. In first-line setting, the small cohort 2 of the KEYNOTE-059 trial in PD-L1 positive and HER-2 negative patients (n=25), tested the combination of pembrolizumab with fluoropyrimidine and platinum. The ORR with pembrolizumab was 60% in the overall population (Fuchs et al. 2018) with 73,3% in PD-L1-positiv tumors compared to 37,5% in PD-L1-negativ tumors. The median survival was 13.8 months with an estimated 1- year survival rate of 55% for the overall population. However, in the combination arm of the KEYNOTE- 062 trial, pembrolizumab plus chemotherapy was not found to be superior for OS (CPS ≥1; (12.5 vs 11.1 months, HR 0.85) or CPS ≥ 10 (12.3 vs. 10.8 months; HR 0.85) or PFS compared with chemotherapy alone (Tabernero et al. 2019). Of note, objective response rates tended to be higher in the combination arm for both CPS ≥ 1 and CPS ≥ 10 (49% vs. 37% and 53% vs. 38%, respectively). The corresponding nivolumab trial is conducting in an PD-L1 all-comer first-line population, although again OS in PD-L1 positive patients serves as primary endpoint (Checkmate-649) (Moehler et al. 2017, Janjigian et al. 2021). In addition, preliminary results of the phase II/III ATTRACTION-04 trial provided interim efficacy data for nivolumab in combination with oxaliplatin and S-1 or capecitabine, with an ORR of 67% and 71% respectively (Kang et al. 2017).

Of note, the favorable toxicity profile makes immunotherapies an interesting partner in combination with chemotherapy. On the other hand, continuous oxaliplatin administration leads to significant toxicity and patient inconvenience. In particular, oxaliplatin-induced cumulative dose is clinically relevant. The alternating approach of chemo- and immunotherapy will reduce cumulative chemotherapy toxicities, especially cumulative dose-dependent neurotoxicity caused by continuous oxaliplatin administration.

We suggest that the implementation of the new sequential cohort Arm A2 has a cumulative therapeutic advantage in terms of increased effectiveness and lowering of toxicity, both from chemotherapy and immunotherapy.

1.7 Rationale for the Combination of Checkpoint Inhibition and Triplet Chemotherapy (Arm C-FLOT Cohort)

Emerging data from recent phase III trials indicate that immune checkpoint inhibitors such as Nivolumab and Pembrolizumab prolong OS and PFS when added to the doublet chemotherapy. However, the extent of improvement regarding PFS is smaller than expected and if administered as monotherapy, there is a an increased early mortality with the checkpoint inhibitors as compared with

chemotherapy (crossing survival curves) (Tabernero et al. 2019; Janjigian et al. 2021, Kato et al. 2020). This is most likely explained by the fact that patients need time to establish antitumoral immunity, while some patients with aggressive disease experience early disease progression and death. This provides a rationale to test whether the intensification of chemotherapy using a triplet (mFOLFOX plus Docetaxel = FLOT) instead of the doublet, while reducing immunotherapy to Nivolumab instead of Nivolumab and Ipilimumab would be more beneficial and safer. Therefore, Arm C is designed to evaluate the efficacy of the combination of FLOT plus Nivolumab in the same patient group of the other study arms and descriptively compare this treatment with mFOLFOX plus Nivolumab plus Ipilimumab (Arm A) and with mFOLFOX alone (Arm B).

As our study generally aims at gaining insights into the potentially most optimal chemo-immunotherapy regimen (therapy optimization) for a future trial, the implementation of Arm C fits well into the concept of the trial. With Arm C our trial evaluates three variations for the concept of immunochemotherapy: chemotherapy doublet plus immunotherapy doublet administered in parallel, chemotherapy doublet plus immunotherapy doublet administered sequentially, and chemotherapy triplet plus immune monotherapy administered in parallel.

Because Arms A and B are fully recruited, it is not possible to perform a randomized comparison for Arm C. However, using the patients from Arms A and B as a comparator is still better than historical controls as these patients are treated by the same centres, under the same therapeutic and diagnostic guidelines, and in a close time period.

It is important to note that FLOT is a well established standard of care triplet regime in both, the neoadjuvant and metastatic settings. Accordingly, according to current S3 guidelines, docetaxel-containing triplet therapy such as FLOT can be considered for patient treatment, based on numerous studies on this field (e.g. van Cutsem et al., 2006 and Wagner et al., 2017).

Approximately 20-30% of patients with mGC in the US (Davis et al NCCN Network Annual Conference and Abrams, ESMO GI 2016# PD-034) and a comparable proportion of patients in Germany receive a taxane-containing triplet as first-line therapy. Rationale for this in most cases is an expected faster and more frequent treatment response, which is crucial for the therapy choice especially for patients with a high remission pressure.

1.8 Overall Risk/Benefit Assessment

Subjects with advanced or metastatic GC and GEJ cancer present a great unmet need. Preliminary results from CA209032, in which nivolumab-plus-ipilimumab treatment was administered to heavily pretreated subjects with advanced/metastatic GC and GEJ cancers, demonstrated clinical activity in subjects whose tumors did or did not express PD-L1; better results were obtained in subjects with PD-L1 expressing (PD-L+) tumors. In addition, in various other tumor types, nivolumab alone and in combination with ipilimumab demonstrated clinical benefits independent or dependent of PD-L1 expressing status. Preliminary data from NSCLC study suggests that nivolumab in combination with platinum doublet has additive anti-tumor activity in subjects regardless of PD-L1 expression, but numerically higher ORR observed in non-squamous NSCLC (Rizvi and Hellmann 2016). Based on this clinical experience, the AIO-STO-0417 trial will enroll patients regardless of PD-L1 status.

The safety profile of nivolumab combined with ipilimumab in GC/GEJ cancer, as measured by total and Grade 3/4 AEs, is comparable to that of a 1L platinum doublet. The safety profile of this nivolumab-plus-ipilimumab combination is characterized by immune-related toxicities, such as diarrhea, rash, pneumonitis, liver toxicity, and endocrinopathies. These events were mostly low grade and manageable with the use of corticosteroids. The ongoing internal and external studies of PD-L1 inhibitors in combination with chemotherapy in treatment-naive advanced GC/GEJ subjects suggest that this combination is tolerable and manageable, no new safety signals being detected. The safety profile of the combination used in the AIO-STO-0417 trial in GC and GEJ cancer is consistent with that reported in subjects with previously untreated melanoma. Recently, encouraging efficacy and manageable safety was reported for the combination of Pembrolizumab with Cisplatin and 5-FU in treatment naïve advanced GC subjects treated in cohort 2 of the KEYNOTE-059 trial (Bang et al 2017). For the sequential cohort, an even more favorable toxicity profile is anticipated.

To ensure an ongoing favorable risk/benefit assessment for subjects enrolled in the AIO-STO-0417 trial, an independent Data Monitoring Committee (DMC) will be utilized to monitor the safety and clinical activity of the treatments throughout the conduct of the trial.

2. Study Objective

The primary objective is to determine the clinical performance of the experimental regimen in patients with previously untreated HER2 negative locally advanced or metastatic esophagogastric adenocarcinoma in terms of progression free survival (acc. to RECIST v1.1).

Secondary objectives are to determine efficacy in terms of objective response rate (acc. to RECIST v1.1) and overall survival, as well as tolerability (acc. to NCI CTC AE v4.03) of the experimental regimen. In addition histopathological types and molecular parameters such as immune cell composition and PD-L1 expression as determined by quantitative mRNA (RT-PCR) will be correlated with efficacy in an exploratory analysis.

3. Study Design

This is a randomized, open labelled multicenter phase II trial followed by a non-randomized arm.

3.1 Primary Endpoint

The primary endpoint is:

- Progression Free Survival acc. to RECIST v1.1 based on the ITT population for patients treated with mFOLFOX plus Nivolumab plus Ipilimumab (Arm A) vs. patients treated with mFOLFOX alone (Arm B) and progression-free survival rate (PFS@6) for Arms A2 and C.

3.2 Secondary Endpoints

The secondary endpoints will include:

- Progression Free Survival acc. to RECIST v1.1 for Arms A1, A2 and C
- Progression Free Survival rate at 6 months (PFS@6) for Arms A and B
- Overall Response Rate (ORR) according to RECIST v1.1
- Duration of response and disease stabilization
- Overall survival (OS)
- Subgroup analysis including PFS and OS by PD-L1 expression status
- Safety (according to NCI-CTCAE V 4.03) and tolerability
- Quality of life (EORTC QLQ-C30). The QoL analyses will include QoL mean values, QoL response and time to symptom deterioration (TTSD)
- Translational research: correlation of biomarkers potentially associated with clinical efficacy (OS, PFS and ORR) from nivolumab plus/minus ipilimumab by molecular quantitation of target gene expression and immune cell composition (e.g. CTLA4, PD1, PDL1, PDL2, CD80, CD4, CD8, CD68, IGKC, CXCL9, CXCL10, CXCL13) within the tumor microenvironment and exploratory analyses based on known histological subtypes (e.g. diffuse vs. intestinal or G1/2 vs. G3) and immune related features (EBV status, MSI status, mutational load).

4. Study Population

4.1 Number of Patients

207 patients will be randomized in Arm A and B, A1 and A2; 50 patients will be included in Arm C. Patients withdrawn from the trial will not be replaced.

4.2 Selection criteria

Patients will be enrolled into the trial according to the selection criteria in section 4.2.1 and 4.2.2.

4.2.1 Inclusion criteria

1. All subjects must have inoperable, advanced or metastatic GC or GEJ adenocarcinoma.
2. Subjects must have HER2-negative disease defined as either IHC 0 or I+ or IHC 2+, the latter in combination with ISH-, as assessed locally on a primary or metastatic tumour.
3. Subject must be previously untreated with systemic treatment given as primary therapy for advanced or metastatic disease.

4. Prior adjuvant or neoadjuvant chemotherapy, radiotherapy and/or chemoradiotherapy are permitted as long as the last administration of the last regimen (whichever was given last) occurred at least 6 months prior to randomization/enrolment.
5. Palliative radiotherapy is allowed and must be completed 2 weeks prior to randomization/enrolment.
6. Subjects must have measurable or evaluable non-measurable disease as assessed by the investigator, according to RECIST v1.1 (Appendix D).
7. ECOG performance status score of 0 or 1 (Appendix B).
8. Life expectancy > 12 weeks
9. Screening laboratory values must meet the following criteria (using NCI CTCAE v.4.03):
    a. WBC ≥ 2000/uL
    b. Neutrophils ≥ 1500/µL
    c. Platelets ≥ $100x10^3$/µL
    d. Hemoglobin ≥ 9.0 g/dL
    e. Serum creatinine ≤ 1.5 x ULN
    f. AST ≤ 3.0 x ULN (or ≤ 5.0X ULN if liver metastates are present)
    g. ALT ≤ 3.0 x ULN (or ≤ 5.0X ULN if liver metastates are present)
    h. Total Bilirubin ≤ 1.5 x ULN (except subjects with Gilbert Syndrome who must have a total bilirubin level of < 3.0 x ULN)
10. Males and Females* ≥ 18 years of age

    *There are no data that indicate special gender distribution. Therefore patients will be enrolled in the study gender-independently.
11. Subjects must have signed and dated an IRB/IEC approved written informed consent form in accordance with regulatory and institutional guidelines. This must be obtained before the performance of any protocol-related procedures that are not part of normal subject care.
12. Subjects must be willing and able to comply with scheduled visits, treatment schedule, laboratory tests and other requirements of the study.
13. Women of childbearing potential (WOCBP) must have a negative serum or urine pregnancy test (minimum sensitivity 25 IU/L or equivalent units of HCG) within 24 hours prior to the start of study drug. Women must not be breastfeeding.
14. WOCBP must agree to follow instructions for method(s) of contraception for a period of 30 days (duration of ovulatory cycle) plus the time required for the investigational drug to undergo 5 half-lives. The terminal half-lives of nivolumab and ipilimumab are approximately 25 days and 15 days, respectively. WOCBP should use an adequate method to avoid pregnancy for approximately

5 months (30 days plus the time required for nivolumab to undergo 5 half-lives) after the last dose of investigational drug.

15. Males who are sexually active with WOCBP must agree to follow instructions for method(s) of contraception for a period of 90 days (duration of sperm turnover) plus the time required for the investigational drug to undergo 5 half-lives. The terminal half-lives of nivolumab and ipilimumab are approximately 25 days and 15 days, respectively. Males who are sexually active with WOCBP must continue contraception for approximately 7 months (90 days plus the time required for nivolumab to undergo 5 half-lives) after the last dose of investigational drug. In addition, male subjects must be willing to refrain from sperm donation during this time.

Notes regarding reproductive status:

Azoospermic males are exempt from contraceptive requirements. WOCBP who are continuously not heterosexually active are also exempt from contraceptive requirements, but still must undergo pregnancy testing as described in this section.

Investigators shall counsel WOCBP and male subjects who are sexually active with WOCBP on the importance of pregnancy prevention and the implications of an unexpected pregnancy.

Investigators shall advise on the use of highly effective methods of contraception (see table 2), which have a failure rate of < 1% when used consistently and correctly.

Table 2: Methods of contraception

HIGHLY EFFECTIVE METHODS OF CONTRACEPTION

- Hormonal methods of contraception including oral contraceptive pills (combination of estrogen and progesterone), vaginal ring, injectables, implants and intrauterine devices (IUDs)
- Nonhormonal IUDs, such as ParaGard®
- Bilateral tubal ligation
- Vasectomy
- Complete Abstinence*

*Complete abstinence is defined as complete avoidance of heterosexual intercourse and is an acceptable form of contraception for all study drugs. Acceptable alternate methods of highly effective contraception must be discussed in the event that the subject chooses to forego complete abstinence.

UNACCEPTABLE METHODS OF CONTRACEPTION

- Diaphragm with spermicide
- Cervical cap with spermicide
- Vaginal sponge

- Condom
- Withdrawal (coitus interruptus)
- Progestin only pills by WOCBP subject or male subject's WOCBP partner
- Periodic abstinence (calendar, symptothermal, post-ovulation methods)

4.2.2 Exclusion criteria

1. Malignancies other than disease under study within 5 years prior to inclusion, with the exception of those with a negligible risk of metastasis or death (e.g., expected 5-year OS > 90%) treated with expected curative outcome (such as adequately treated carcinoma in situ of the cervix, basal or squamous cell skin cancer, localized prostate cancer treated surgically with curative intent, ductal carcinoma in situ treated surgically with curative intent)
2. Subjects with untreated symptomatic CNS metastases. Subjects are eligible if CNS metastases are asymptomatic (this includes patients with unknown CNS metastatic status who have no clinical signs of CNS metastases) or those with asymptomatic or symptomatic CNS who are adequately treated and are neurologically returned to baseline (except for residual signs or symptoms related to the CNS treatment) for at least 2 weeks prior to randomization/enrolment. In addition, subjects must be either off corticosteroids, or on a stable or decreasing dose of < 10 mg daily prednisone (or equivalent) for at least 2 weeks prior to randomization/enrolment. Patients with unknown CNS metastatic status and any clinical signs indicative of CNS metastases are eligible if CNS metastases are excluded using CT and/or MRI scans, or CNS metastases are confirmed but adequately treated as described above.
3. Subjects with active, known, or suspected autoimmune disease. Subjects with Type I diabetes mellitus, residual hypothyroidism due to autoimmune thyroiditis only requiring hormone replacement, or skin disorders (such as vitiligo, psoriasis, or alopecia) not requiring systemic treatment are permitted to enroll. For any cases of uncertainty, it is recommended that the medical monitor be consulted prior to signing informed consent.
4. Subjects with a condition requiring systemic treatment with either corticosteroids (> 10 mg daily prednisone equivalents) or other immunosuppressive medications within 14 days of study drug administration. Inhaled or topical steroids, and adrenal replacement doses > 10 mg daily prednisone equivalents are permitted in the absence of active autoimmune disease.
5. Prior treatment with an anti-PD-1, anti-PD-L1, anti-PD-L2, anti-CD137, or anti-CTLA-4 antibody, or any other antibody or drug specifically targeting T-cell co-stimulation or checkpoint pathways.
6. All toxicities attributed to prior anti-cancer therapy other than hearing loss, alopecia and fatigue must have resolved to Grade 1 (NCI CTCAE version 4.03) or baseline before administration of study drug.
7. > Grade 1 peripheral neuropathy according to CTCAE version 4.0
8. Known Dihydropyrimidine dehydrogenase (DPD) deficiency

9. Any serious or uncontrolled medical disorder or active infection that, in the opinion of the investigator, may increase the risk associated with study participation, study drug administration, or would impair the ability of the subject to receive study drug.
10. Ascites which cannot be controlled with appropriate interventions.
11. Unstable cardiac disease despite treatment, myocardial infarction within 6 months prior to study entry; congestive heart failure NYHA grade 3 and 4
12. Significant acute or chronic infections including, among others:

    a. Positive test for human immunodeficiency virus (HIV) or known acquired immunodeficiency syndrome (AIDS).

    b. Any positive test result for hepatitis B virus or hepatitis C virus indicating acute or chronic infection.
13. History of allergy or hypersensitivity to study drugs or any constituent of the products
14. Patient who has been incarcerated or involuntarily institutionalized by court order or by the authorities § 40 Abs. 1 S. 3 Nr. 4 AMG.
15. Patients who are unable to consent because they do not understand the nature, significance and implications of the clinical trial and therefore cannot form a rational intention in the light of the facts [§ 40 Abs. 1 S. 3 Nr. 3a AMG].

4.2.3 Women of Childbearing Potential

Women of childbearing potential (WOCBP) is defined as any female who has experienced menarche and who has not undergone surgical sterilization (hysterectomy or bilateral oophorectomy) and is not postmenopausal. Menopause is defined as 12 months of amenorrhea in a woman over age 45 years in the absence of other biological or physiological causes. In addition, females under the age of 55 years must have a serum follicle stimulating hormone (FSH) level > 40 mIU/mL to confirm menopause.

*Females treated with hormone replacement therapy (HRT) are likely to have artificially suppressed FSH levels and may require a washout period in order to obtain a physiologic FSH level. The duration of the washout period is a function of the type of HRT used. The duration of the washout period below are suggested guidelines and the investigators should use their judgement in checking serum FSH levels.

- 1 week minimum for vaginal hormonal products (rings, creams, gels)
- 4 week minimum for transdermal products
- 8 week minimum for oral products

Other parenteral products may require washout periods as long as 6 months. If the serum FSH level is > 40 mIU/mL at any time during the washout period, the woman can be considered postmenopausal.

5. Study Procedures and Methodology

5.1 Overall Study Schedule Overview

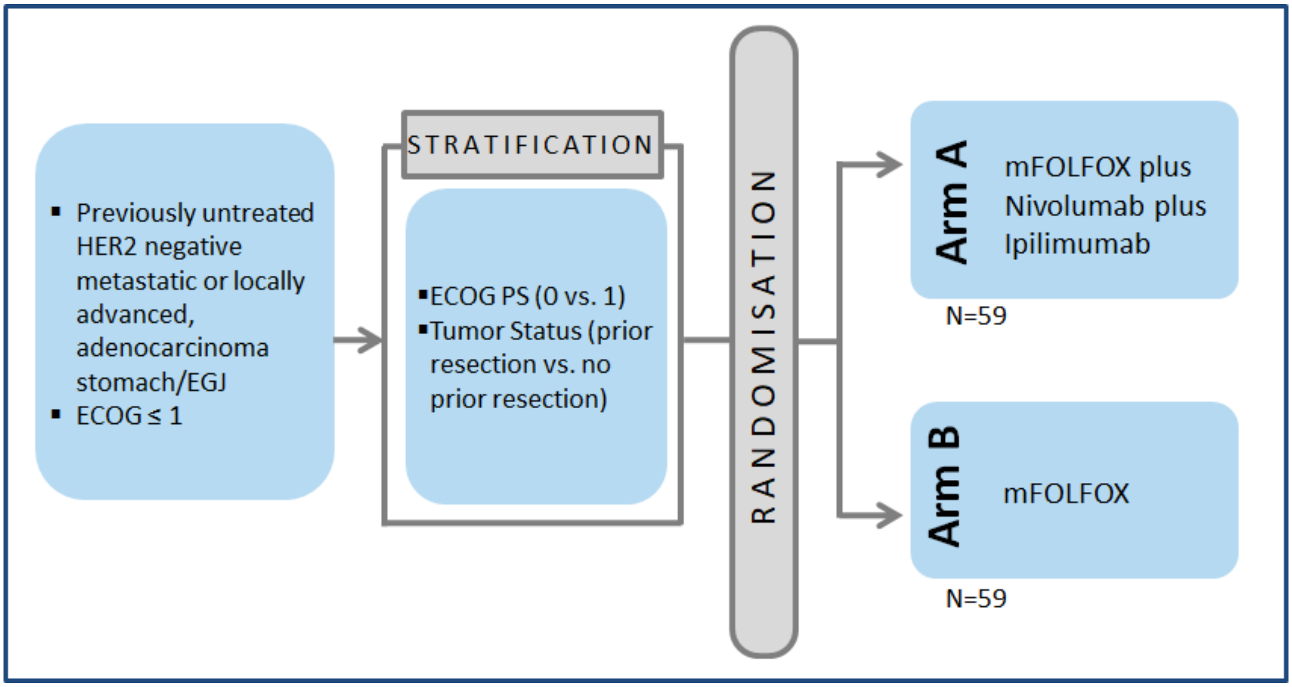

Figure 1: Overall study schedule overview for Arm A vs. B

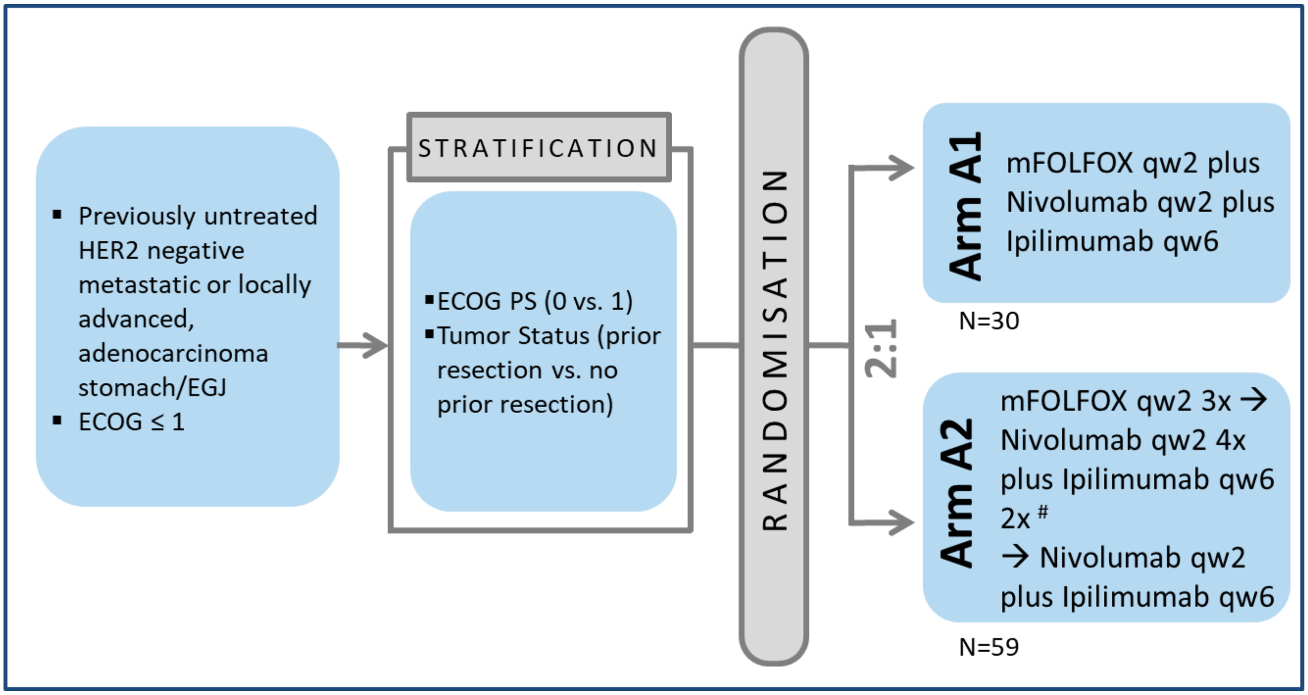

Figure 2: Overall study schedule overview for Arm A1 vs. A2

in Arm A2, upon investigator´s decision the sequence consisting of mFOLFOX 3x followed by Nivolumab 4x plus Ipilimumab 2x may be repeated as long as medically reasonable. After discontinuation of

chemotherapy, immunotherapy will be continued with Nivolumab every 2 weeks and Ipilimumab every 6 weeks.

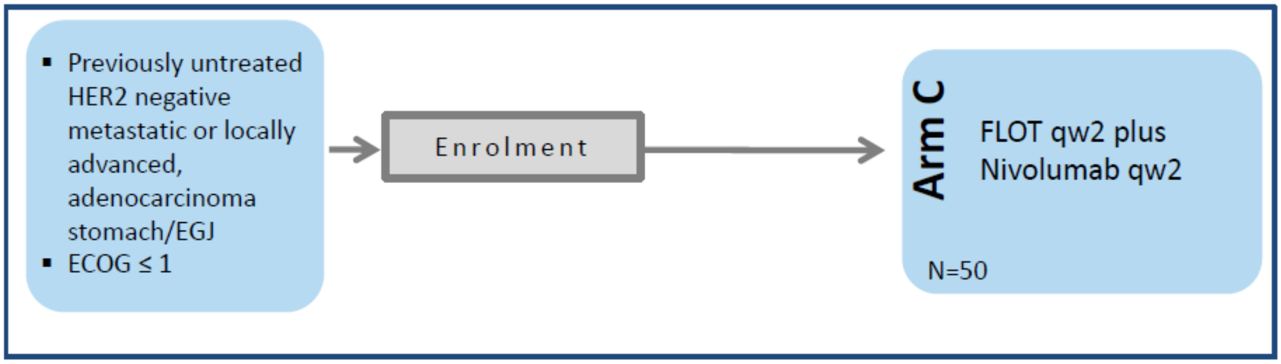

Figure 3: Overall study schedule overview for Arm C

5.2 Treatment

5.2.1 Randomization/enrolment

After inclusion in the treatment phase patients will be randomized to arm A or B in a 1:1 ratio into Arm A and B for the first 118 patients and in a 1:2 ratio into Arm A1 and A2 in the subsequent 89 patients stratified according to the following criteria

- ECOG PS (0 vs 1)
- Tumor status (prior resection vs. no prior resection)

Arm C (FLOT cohort) will be single arm including subsequent 50 patients.

5.2.2 Investigational Product

In this protocol, the investigational products are Nivolumab solution for injection and Ipilimumab solution for injection, Oxaliplatin solution for injection, Fluorouracil solution for injection, Docetaxel solution for injection (Arm C) and Leucovorin solution for injection. Nivolumab and ipilimumab are not approved for first-line treatment of EGA. Thus, the manufacturer will supply both drugs. Fluorouracil/Leucovorin, Oxaliplatin and Docetaxel (Arm C) is standard of care for the treatment of EGA and will not be supplied or reimbursed. All used agents nivolumab, ipilimumab and Oxaliplatin, Fluorouracil, Leucovorin and Docetaxel will be defined as investigational medicinal product (IMP).

The IMPs (nivolumab, ipilimumab) will be supplied by the central pharmacy and will bear a label with the identification required by local law, the protocol number, drug identification and dosage as well as the statement "For Clinical Trial Use Only". The packaging and labeling of the study medication will be in accordance with local regulations. All the requirements of Annex 13 of the Good Manufacturing Practices guideline for labeling investigational drug will be fulfilled.

5.2.3 Dosing and schedule

All eligible patients will be randomized to

Arm A/A1

Nivolumab 240mg "Flatdose" i.v. d1 over 30 minutes
every 2 weeks until disease progression or inacceptable toxicity

followed by

Ipilimumab 1mg/kg i.v. d1 over 30 minutes
every 6 weeks until disease progression or inacceptable toxicity

followed by

FOLFOX

Oxaliplatin at a dose of 85 mg/m² iv over two hours (day 1)
Leucovorin at a dose of 400 mg/m² iv over two hours (day 1)
Fluorouracil at a dose of 400 mg/m² iv bolus (day 1)
Fluorouracil at a dose of 2400 mg/m² iv continuous infusion over 44 hours (day 1+2)
every 2 weeks until disease progression or inacceptable toxicity or end of study treatment.
Chemotherapy can also be administered per local standard.

Table 3: Dose Schedule of FOLFOX

**Treatment group A + B**	**Drug name**	**Cycle 1 till EOT (Q2 weeks)**
FOLFOX	Oxaliplatin 85 mg/m²	Day 1
	Leucovorin 400 mg/m²	
	Fluorouracil 400 mg/m²	
	Fluorouracil 2400 mg/m²	Day 1 & 2 (44 h)

On the day of infusion, nivolumab has to be administered first. At therapy days planned for ipilimumab (every 6 weeks), the second infusion will be ipilimumab, and will start at the earliest 30 minutes after completion of the nivolumab infusion. Chemotherapy is administered after ipilimumab (if given) or after nivolumab on treatment days planned without ipilimumab. Chemotherapy

procedures will follow local standards (e.g. sequence of substances) as it is considered standard of care.

Therapy can also be splitted, administering nivolumab/ipilimumab on day one and FOLFOX starting at day two of the cycle.

OR

Arm A2

3 cycles of induction chemotherapy with FOLFOX:
FOLFOX
Oxaliplatin at a dose of 85 mg/m² iv over two hours (day 1)
Leucovorin at a dose of 400 mg/m^2 iv over two hours (day 1)
Fluorouracil at a dose of 400 mg/m² iv bolus (day 1)
Fluorouracil 2400 mg/m² iv continuous infusion over 44 hours (days 1+2)
every 2 weeks
See also Table 3 for Dose Schedule of FOLFOX above. Chemotherapy can also be administered per local standard.

Followed by immunotherapy consisting of:

4 administrations of Nivolumab 240mg "Flatdose" i.v. d1 over 30 minutes
every 2 weeks

and

2 administrations of Ipilimumab 1mg/kg i.v. d1 over 30 minutes
every 6 weeks

Repetition of chemotherapy and immunotherapy:

The above described therapy sequence consting of 3 cycles of FOLFOX followed by immunotherapy may be repeated starting two weeks after last administration of immunotherapy once, or, if medically reasonable, for an unlimited number of repetitions upon investigator decision. However, repetition of chemotherapy after the first 3 cycles is optional and may be skipped.

After completion or discontinuation of chemotherapy, **immunotherapy** will be continued consisting of:

Nivolumab at 240mg "Flatdose" i.v. d1 every 2 weeks
and Ipilimumab at 1mg/kg i.v. d1 every 6 weeks

Study therapy as described above will be administered until disease progression, inacceptable toxicity, patient's request, or end of study treatment phase (24 months).

At days where Nivolumab and Ipilimumab are administered, Nivolumab will be applied first and is then followed by Ipilimumab as described for Arm A/A1.

OR

Arm B (closed after a total of 118 patients have been randomized into Arm A + B)

FOLFOX

Oxaliplatin at a dose of 85 mg/m² iv over two hours (day 1)
Leucovorin at a dose of 400 mg/m² iv over two hours (day 1)
Fluorouracil at a dose of 400 mg/m² iv bolus (day 1)
Fluorouracil 2400 mg/m² iv continuous infusion over 44 hours (days 1+2)
every 2 weeks until disease progression or inacceptable toxicity or end of study treatment.

See also Table 3 for Dose Schedule of FOLFOX above. Chemotherapy can also be administered per local standard.

OR enrolled into

Arm C

Nivolumab 240mg "Flatdose" i.v. d1 every 2 weeks until disease progression or inacceptable toxicity

Followed by

FLOT

**Docetaxel**	at a dose of 50 mg/m² iv over one hour (day 1)
**Oxaliplatin**	at a dose of 85 mg/m² iv over two hours (day 1)
**Leucovorin***	at a dose of 200 mg/m² iv over one hour (day 1)
**Fluorouracil**	at a dose of 2600 mg/m² iv over 24 hours (day 1)

* Note: Leucovorin can be replaced by sodium folinate that is given according to local guideline.

Cycles are repeated every 2 weeks until disease progression or inacceptable toxicity or end of study treatment. After completion or discontinuation of chemotherapy, immunotherapy may be continued consisting of:

Nivolumab at 240mg "Flatdose" i.v. d1 every 2 weeks

Chemotherapy can also be administered per local standard.

Therapy can also be splitted, administering nivolumab on day one and FLOT starting at day two of the cycle.

Subjects may be dosed no less than 12 days from the previous dose of drug. There are no premedications recommended for nivolumab and ipilimumab on the first cycle.

For FOLFOX, premedication is not usually required in the first cycle. For subsequent cycles, adequate premedication may be administrated per local standard.

For FLOT, premedication, e.g prophylactic steroids as antiemetic therapy can be given at discretion of the investigator for a maximum of three days per cycle. A modern combination of a neurokinin-1-rezeptor-antagonist and 5-HT3-receptor-antagonist on d1 and a 5-HT3-receptor-antagonist on d2-3 is recommended. Table 4 gives an example for the antiemetic treatment schema.

However, the investigators may administer alternative antiemetic therapy according to local guidelines if equi-effective.

If patients develop nausea and or vomiting of grade 3 or 4 or any grade leading to discontinuation or interruption of treatment, the investigator may add steroids to antiemetic schema the respective cycle and all other subsequent chemotherapy cycles.

Table 4: Schema for antiemetic therapy for FLOT (example). Antiemetic therapy can also be performed according to local guidelines.

Day	Drug class	Example
d1<br>(acute emesis)	Neurokinin-1-rezeptor-antagonist and 5-HT3-receptor-antagonists	<u>Alternatives:</u><br>Aprepitant 125 mg (80 mg d2-3)<br>+ 5-HT3-receptor-antagonists (see d2-3) OR<br>Rolapitant 180 mg<br>+ 5-HT3-receptor-antagonists (see d2-3) OR<br>Netupitant 300 mg + 0.5 mg palonosetron
d2-3<br>(delayed emesis)	5-HT3-receptor-antagonists	<u>Alternatives:</u><br>Granisetron; 2 mg p.o. / 1 mg i.v.<br>Ondansetron; 16-24 mg p.o. / 8 mg i.v.<br>Tropisetron; 5 mg p.o. / i.v.<br>Dolasetron; 200 mg p.o.<br>Palonosetron; 0.5 mg p.o. / 0.25 mg i.v.

Dihydropyrimidine dehydrogenase (DPD) plays an important role in the metabolism of fluorouracil. There have been reports of increased fluorouracil toxicity in patients who have reduced activity/deficiency of DPD.

Fluorouracil (5-FU) must not be given in combination with brivudin, sorivudin and analogues. Brivudin, sorivudin and analogues are potent inhibitors of the 5-FU-metabolising enzyme dihydropyrimidine dehydrogenase (DPD). Fluorouracil (5-FU) must not be given to patients

homozygotic for dihydropyrimidine dehydrogenase [mutations] (DPD). Where applicable, determination of DPD enzyme activity is indicated before starting treatment with 5-fluoropyrimidines.

The dosing calculations should be based on the actual body weight. If the subject's weight on the day of dosing differs by > 10% from the weight used for the last dose calculation, the dose must be recalculated. Recalculation of the chemotherapy dose for body weight changes can also be done according to local guidelines. All doses should be rounded to the nearest milligram. For nivolumab and ipilimumab, no dose modifications are allowed.

Subjects should be carefully monitored for infusion reactions during nivolumab and ipilimumab administration. If an acute infusion reaction is noted, subjects should be managed according to protocol section 6.1.5.

5.2.4 Treatment duration

Treatment with each of the components FOLFOX, FLOT, nivolumab and/or ipilimumab will be administered until progression (according to RECIST v1.1), intolerable toxicity, patient's request,or end of study treatment phase (24 months). The study treatment will be limited to a maximum of 24 months. If one component of the treatment is stopped for any cause, the other components can be continued.

5.2.5 Study medication

The study medications (nivolumab and ipilimumab) will be supplied by the manufacturer and delivered to the local pharmacy for preparation and administration.

Table 5: product information

Product Description/ Class / Dosage Form	Potency	IP/Non-IMP	Packaging / Appearance	Storage Conditions
BMS-936558<br>Nivolumab Solution for Injection	100 mg<br>(10 mg/mL)<br>or<br>40 mg<br>(10 mg/mL)	IP	10 mL per vial<br>(5 or 10 vials per carton)<br>or<br>4 mL/vial<br>(240 mg kits: 2-100 mg vials and 1-40 mg vial)	Store at 2° - 8° C<br>Protect from light and freezing
Ipilimumab Solution for Injection	200 mg<br>(5 mg/mL)	IP	40 mL/vial<br>(4 vials/carton)	Store at 2° - 8° C<br>Protect from light and freezing

Please refer to the current version of the Investigator Brochures and/or pharmacy reference sheets for complete storage, handling, dispensing, and infusion information.

If stored in a glass front refrigerator, vials should be stored in the carton. Recommended safety measures for preparation and handling of nivolumab and ipilimumab include laboratory coats and gloves.

For additional details on prepared drug storage and use time of nivolumab and ipilimumab under room temperature/light and refrigeration, please refer to the BMS-936558 (nivolumab) and BMS-734016 (ipilimumab) Investigator Brochure section for "Recommended Storage and Use Conditions".

The Summary of Product Characteristics (SmPC, "Fachinformation") for the used backbone drug (oxaliplatin, 5-FU, leucovorin, docetaxel) will be supplied to ensure administration of drugs according to SmPC.

5.2.5.1 *Handling and Dispensing*

The investigator should ensure that the study drug is stored in accordance with the environmental conditions (temperature, light, and humidity) as per product information and the Investigator Brochure and per local regulations. It is the responsibility of the investigator to ensure that investigational product is only dispensed to study subjects. The investigational product must be dispensed only from official study sites by authorized personnel according to local regulations. If concerns regarding the quality or appearance of the study drug arise, the study drug should not be dispensed and contact the sponsor/sponsor representative immediately.

Please refer to the current version of the Investigator Brochure and/or shipment reference sheets for additional information on storage, handling, dispensing, and infusion information for BMS-936558 (nivolumab) and BMS-734016 (ipilimumab).

5.2.5.2 *Treamtent administration and study site qualification*

Administration of study treatment will be performed only at study sites after evaluation of the qualification and approval by the authorities. These sites are qualified medical facilities e.g. hospitals with specialized oncology departments and oncology medical private practices. All study sites have to have experience in the oncological treatment of patients with cancer, including the routine use of checkpoint inhibitors, in the use of the administered therapeutical substances and in the conduction of oncological trials.

Administration of study treatment will be done by authorized medical staff, such as investigators and study nurses, who are qualified regarding indication, clinical trial experience and experience in indication and routine administration of infusions according to local standards and local regulations. The study group at the trial site will always be led by the responsible principal investigator and his/her designee whose qualification for conduct of this trial was evaluated and approved by the authorities.

The availability of adequate emergency equipment is mandatory for participation of a trial site and is assured by the qualification statements of the investigational sites. Qualification of the site including emergency equipment is evaluated and approved by the authorities before participation.

5.2.5.3 *Destruction*

Investigator drug destruction is allowed provided the following minimal standards are met:

- On-site disposal practices must not expose humans to risks from the drug.
- On-site disposal practices and procedures are in agreement with applicable laws and regulations, including any special requirements for controlled or hazardous substances.
- Written procedures for on-site disposal are available and followed. The procedures must be filed with the ISF and a copy provided to the sponsor or his representative upon request.
- Records are maintained that allow for traceability of each container, including the date disposed of, quantity disposed, and identification of the person disposing the containers. The method of disposal, ie, incinerator, licensed sanitary landfill, or licensed waste disposal vendor must be documented.
- Accountability and disposal records are complete, up-to-date, and available for the Sponsor/sponsor representative to review throughout the clinical trial period as per the study agreement.

If conditions for destruction cannot be met, please contact the sponsor/sponsor representative.

It is the Investigator's responsibility to arrange for disposal of all empty containers, provided that procedures for proper disposal have been established according to applicable federal, state, local, and institutional guidelines and procedures, and provided that appropriate records of disposal are kept.

5.2.6 Concomitant medication

5.2.6.1 *Permitted therapy*

Concomitant therapy includes any prescribed medications or over-the-counter preparations used by a patient between the 7 days preceding the screening evaluation and the treatment discontinuation visit.

Subjects are permitted the use of topical, ocular, intra-articular, intranasal, and inhalational corticosteroids (with minimal systemic absorption). Adrenal replacement steroid doses > 10 mg daily prednisone are permitted. A brief (less than 3 weeks) course of corticosteroids for prophylaxis (eg, contrast dye allergy) or for treatment of non-autoimmune conditions (eg, delayed-type hypersensitivity reaction caused by a contact allergen) is permitted.

All concomitant medications should be reported to the investigator and recorded on the appropriate CRF at baseline.

5.2.6.2 *Prohibited Therapy*

The following medications are prohibited during the study (unless utilized to treat a drug related adverse event):

- Immunosuppressive agents
- Immunosuppressive doses of systemic corticosteroids (except as stated in Section 4.2.2)
- Any concurrent anti-neoplastic therapy (ie, chemotherapy, hormonal therapy, immunotherapy, extensive, non-palliative radiation therapy, or standard or investigational agents for treatment of GC/GEJ).

The concomitant use of herbal therapies is not recommended because their pharmacokinetics, safety profiles, and potential drug-drug interactions are generally unknown.

Fluorouracil (5-FU) must not be given in combination with brivudin, sorivudin and analogues. Brivudin, sorivudin and analogues are potent inhibitors of the 5-FU-metabolising enzyme dihydropyrimidine dehydrogenase (DPD).

The above list of medications is not necessarily comprehensive. The investigator should consult the prescribing information for any concomitant medication and contact the coordinating investigator or sponsor/sponsor representative if questions arise regarding medications not listed above.

5.3 Assessments and Guidelines for Visits

5.3.1 Baseline assessments (within 4 weeks before treatment start)

Consenting patients will have the following screening/baseline assessments performed within 4 weeks prior to the first treatment.

- Her2 assessment (local); already available investigations can be used for the study
- Review of inclusion and exclusion criteria
- Medical and medication history, physical examination including height, weight, ECOG-performance status and concomitant medication
- Laboratory Tests:
    - Hematology panel: hemoglobin, platelets, WBC with neutrophils, lymphocytes, monocytes, eosinophils, and basophils
    - Chemistry panel: sodium, potassium, calcium, magnesium, serum creatinine, urea, alkaline phosphatase, AST, ALT, total and direct bilirubin, glucose, lipase, amylase, LDH
    - Coagulation: INR, aPTT
    - Free T3/T4 and TSH
    - CEA, CA 19-9
    - Hepatitis B/C screening test (HBsAg, anti-HBc, anti-HBs, anti-HCV)
    - HIV testing within 7 days prior to start of the treatment
    - Pregnancy test for women of childbearing potential within 24 hours prior to start of the treatment

- Blood draw (2 EDTA tubes a 2 mL)
- Obtain paraffin-embedded tumor-tissue for PD-L1 assessment and translational research. Centers may send either a paraffin block or slices. If slices are sent, please send 1x HE stained slice, 4 x 5 µm unstained tissue slices on glass slides.
- ECG
- Quality of life assessment (EORTC QLQ-C30)
- Disease assessment by radiological imaging of the chest, abdomen, pelvis and all other sites of disease or any clinically indicated sites (CT/MRI-scan). Already available investigations can be used for the study if within the time frame.

The investigator will confirm the patient's eligibility after all baseline scans and laboratory results have been reviewed.

5.3.2 Assessments during treatment

5.3.2.1 *Assessment at start of treatment and every 2 weeks (+3/-2 days) thereafter*

The following assessments will be made previous to any new cycle. The baseline assessments may be used if within 3 days of day 1 cycle 1. Safety-relevant assessments, including pregnancy test have to be completed before dosing.

- Physical examination including height, weight, ECOG-performance status, assessment of toxicity
- Laboratory tests
  - Hematology panel: hemoglobin, platelets, WBC with neutrophils, lymphocytes, monocytes, eosinophils, and basophils
  - Chemistry panel: sodium, potassium, calcium, magnesium, serum creatinine, urea, alkaline phosphatase, AST, ALT, total and direct bilirubin, glucose, lipase, amylase, LDH
  - Free T3/T4 and TSH (after every 4th 2-week cycle = approximately every 8 weeks)
  - Pregnancy test for women of childbearing potential (after every 4th 2-week cycle = approximately every 8 weeks)
  - CEA, CA 19-9 (only every 8 weeks, together with imaging)
- Quality of life assessment (EORTC QLQ-C30) after every 4th cycle (= approximately every 8 weeks (prior to imaging) until EOT, afterwards every 3 months)
- Disease assessment (tumor response assessment) by radiological imaging of the chest, abdomen, pelvis and all other sites of disease (CT/MRI-scan) (every 8 weeks until EOT)

5.3.2.2 *Tumor response assessment (every 8 weeks ±7 days)*

During treatment tumor response will be assessed by the investigator according to RECIST v1.1 (Radiological imaging by CT and/or MRI of the chest, abdomen, pelvis and all other sites of disease) after every 4th cycle, which represents the standard of care in Germany. After treatment discontinuation for reasons other than progressive disease imaging will be performed every 3 months until progression or death, which represents the standard of care in Germany.

5.3.2.3 *Final staging (end of treatment)*

The following assessments will be made if patient discontinues treatment due to progression (e.g. lack of therapeutic efficacy), severe toxicity disabling further treatment continuation or severe adverse events related to the treatment.

- Physical examination including height, weight, ECOG-performance status, assessment of toxicity
- Laboratory tests
    - o Hematology panel: hemoglobin, platelets, WBC with neutrophils, lymphocytes, monocytes, eosinophils, and basophils
    - o Chemistry panel: sodium, potassium, calcium, magnesium, serum creatinine, urea, alkaline phosphatase, AST, ALT, total and direct bilirubin, glucose, lipase, amylase, LDH
    - o Coagulation: INR, aPTT
    - o Free T3/T4 and TSH
    - o Pregnancy test for women of childbearing potential
    - o CEA, CA 19-9
- ECG
- Quality of life assessment (EORTC QLQ-C30)
- Disease assessment by radiological imaging of the chest, abdomen, pelvis and all other sites of disease (CT/MRI-scan)

NOTE: Tumor assessment using CT/MRI and QoL-assessment are not a mandatory part of the EOT if last assessment is not older than 6 weeks. Except for toxicity assessment, further EOT examinations may be skipped if results of examinations are available which are not older than 14 days at EOT.

5.3.3 Extended safety follow-up: 30 days and 100 day safety follow-up (±7 days)

- Physical examination, performance status (ECOG), assessment of toxicity

- Laboratory tests (hematology and chemistry panel as above), including free T3/T4 and TSH and pregnancy test for women of childbearing potential

Given the potential risk for delayed immune-related toxicities, safety follow-up must be performed at 30 and 100 days after the last dose of study therapy administration (all arms). The extended monthly safety follow-up beyond 30 days after last study drug administration may be performed either via a site visit or via a telephone call with subsequent site visit requested in case any concerns noted during the telephone call. If there are ongoing adverse events on site visits including laboratory tests will be conducted.

5.3.4 Individual Follow-up

All subjects will be followed every 3 months ± 28 days for up to four years after start of recruitment (this is the individual follow-up, which differs from the over all minimum follow up determined by the statistical calculation and which is 3 years consisting of 2 years recruitment and 1 year follow up after last patient-in).

In case of progressive disease after study treatment only:

- Survival, disease status, protracted toxicity, further treatment

In any other case additionally:

- Physical examination including weight, ECOG-performance status
- Disease assessment (every 3 months)
- Quality of life assessment (EORTC QLQ-C30) (every 3 months)

5.4 Study Duration

Study duration is planned as follows:

Recruitment period	45 months
Follow up (starting from last patient in)	12 months
Further follow up	6 months
Overall trial duration/maximum duration per individual patient	63 months

5.5 End of Trial

All randomized/enrolled patients will be followed up for at least 12 months from the date of randomization/enrolment until the individual end of the study or for a maximum of 4 years. After follow-up, the data cleaning process will be finalized and database will be closed.

Database closure is defined as the end of the trial: Sites need to collect survival data of patients and are involved in the data cleaning process actively (e.g. additional source data may be requested and additonal monitoring visits may be neccessary). Therefore, database closure is considered the end of the trial.

5.6 Study Termination

5.6.1 Interim Safety Analysis

In this trial, there will be an interim safety analysis conducted when the first 15 patients have been enrolled into the experimental study arm and treated for at least 6 weeks. Safety data of the first 15 patients in Arm A and the corresponding patients in Arm B will be analysed based on a comprehensive analysis plan and reviewed by the lead investigators (Prof. Götze and Dr. Lorenzen) and by the IDMC. Recruitment will not be stopped during this analysis. In case the recruitment is stopped prematurely for toxicity after the safety analysis, there will be a meeting of the steering committee to decide on further steps. The steering committee can decide to permanently discontinue the trial or to amend the protocol.

5.6.2 Patient Withdrawal

Patients will be withdrawn from therapy based on the following reasons:

- Treatment for max. 24 months was received (treatment completed)
- Post-consent determination of ineligibility based on safety criteria
- Lack of therapeutic efficacy, as evidenced by progression
- Treatment related toxicity according to dose modification criteria (section 6)
- Pregnancy
- Physician's judgment following an adverse event
- Termination by the sponsor, or a regulatory authority
- Any other reason for withdrawal that the study physician or patient indicates is in the overall best interest of the patient

After withdrawal of therapy, follow-up has to be continued up to the end of trial.

The trial participation of a patient terminates at the regular end of trial or on her/his own wish. If a patient withdraws the consent to study participation, the follow-up ends at the day the patient determines. Patients who voluntarily withdraw consent or who are withdrawn by the study physician for any reason after receiving therapy will be followed-up for the regular post-dose follow up period (i.e. 30 and 100 days), if patient agrees. The discussion on participation in the FUP period after discontinuation of treatment should be documented in the patient file. If feasible (only withdrawal of trial participation, but not for follow up) patients will be followed by clinical visitations or telephone contact post withdrawal for assessment of overall survival.

If a patient dies prior to the last scheduled study visit, the date and cause of death will be recorded.

5.6.3 Study Completion

The total duration of recruitment is estimated to be 45 months. Follow-up time is 12 months, starting from last patient in. Six additional months are needed for analysis.

6. Dose Modifications

6.1 General Remarks

- Toxicity will be graded according to NCI CTCAE, version 4.03 (Appendix C). Treatment modifications described below are applied according to this severity grading. Toxicities of severity grade 1 only will not lead to any dose reduction or cycle delay. The same holds for adverse reactions without any potential of serious or life-threatening complications according to the judgment of the physician (e.g. alopecia).
- Presumably, severe overlapping toxicity between Folfox, FLOT and the check point inhibitor(s) (nivolumab and/or ipilimumab) will not occur. Thus, in case of toxicity requiring treatment modification, this alteration should reflect the causal relationship of the respective drug(s). For example, if the toxicity is unequivocally caused by only Folfox, a dosage modification of the other drugs is not required. This does not apply for nivolumab and ipilimumab, which have overlapping toxicities resulting in dose modification of both drugs.
- If toxicity requires a dosing delay or interruption of any drug for more than 6 weeks, that drug should be discontinued. The patient can remain on study with the remaining drugs and will continue to be evaluated according to study procedures.
- If more than one different type of toxicity occurs concurrently, the most severe grade will determine the modification.
- No dose reductions or dose escalations are permitted in this trial for the check point inhibitors (nivolumab and/or ipilimumab) (besides adaptation for body weight changes). For chemotherapy, dose modifications are described separately in section 6.1.5.
- In case of acute allergic reactions of grade 3 or 4, the respective agent should be discontinued permanently; in case of grade 1 or 2, it is up to the physician to continue treatment without dose modification, if this is in the best interest of the patient.
- Each treatment modification or delay has to be documented in the CRF, including the respective reason.
- According to investigators´ decision, Ipilimumab can (should) be discontinued and treatment can (should) be continued with Nivolumab alone in case of drug-related AEs not fulfilling the criteria for discontinuation of both drugs but would appear inaccepable for the investigators.

It is allowed to administer either nivolumab or ipilimumab or chemotherapy doublet or triplet or single drug alone if the other(s) are discontinued due to toxicity, judging by the treating investigator

if it is the best interest in the subjects. The assessments for discontinuation of nivolumab/ipilimumab and/or chemotherapy should be made separately. It is allowed to continue nivolumab/ipilimumab alone when chemotherapy has been discontinued due to toxicity. Chemotherapy doublet or single drug is allowed to continue if nivolumab/ipilimumab met its discontinuation criteria.

6.1.1 Dose Delay Criteria

Because of the potential for clinically meaningful AEs, related to administration of nivolumab alone or in combination with ipilimumab, requiring early recognition and prompt intervention, management algorithms have been developed for suspected AEs of selected categories (please refer to the IB (Investigator Brochure) for Nivolumab).

Dose delay criteria apply for all drug-related adverse events (regardless of whether or not the event is attributed to nivolumab alone or in combination with ipilimumab). All study drugs must be delayed until treatment can resume.

Administration of Nivolumab alone or in Combination with Ipilimumab should be delayed for the following:

Any Grade 3 skin, drug-related AE

Any Grade ≥ 2 non-skin, drug-related AE, with the following exceptions:

- Grade 2 drug-related fatigue or laboratory abnormalities do not require a treatment delay

Any Grade 3 drug-related laboratory abnormality, with the following exceptions for lymphopenia, leukopenia, AST, ALT, total bilirubin, or asymptomatic amylase or lipase:

- Grade 3 lymphopenia or leukopenia does not require dose delay.
- If a subject has a baseline AST, ALT, or total bilirubin that is within normal limits, delay dosing for drug-related Grade ≥ 2 toxicity.
- If a subject has baseline AST, ALT, or total bilirubin within the Grade 1 toxicity range, delay dosing for drug-related Grade ≥ 3 toxicity.
- Any Grade ≥ 3 drug-related amylase or lipase abnormality that is not associated with symptoms or clinical manifestations of pancreatitis does not require dose delay. The Investigator should be consulted for such Grade ≥ 3 amylase or lipase abnormalities.

Any AE, laboratory abnormality, or intercurrent illness which, in the judgment of the investigator, warrants delaying the dose of study medication.

Subjects who require delay of nivolumab or nivolumab in combination with ipilimumab should be re-evaluated weekly or more frequently if clinically indicated and resume dosing when re-treatment criteria are met.

Requirements for FLOT chemotherapy administration

Centers can administer FLOT chemotherapy if:

- leukocytes are at ≥ 3.000/mm³* and
- thrombocytes are at ≥ 100.000/mm³ and
- relevant non-hematologic toxicity is < grade 2 and
- in the absence of fever or a relevant infection

*Note: For FLOT therapy, the overall number of leukocytes is relevant. Treatment can be continued if leukocytes are at 3.000/µl or higher or neutrophils ≥ 1.000/µl .

If above mentioned criteria are not met on day 1, patients should receive the supportive care required according to local protocols until all requirements for treatment continuation are met.

6.1.2 Criteria to Resume Treatment

Subjects may resume treatment with study drug when the drug-related AE(s) resolve to Grade≤1 or baseline value, with the following exceptions:

- Subjects may resume treatment in the presence of Grade 2 fatigue
- Subjects who have not experienced a Grade 3 drug-related skin AE may resume treatment in the presence of Grade 2 skin toxicity
- Subjects with baseline Grade 1 AST/ALT or total bilirubin who require dose delays for reasons other than a 2-grade shift in AST/ALT or total bilirubin may resume treatment in the presence of Grade 2 AST/ALT OR total bilirubin
- Subjects with combined Grade 2 AST/ALT AND total bilirubin values meeting discontinuation parameters should have treatment permanently discontinued
- Drug-related pulmonary toxicity, diarrhea, or colitis, must have resolved to baseline before treatment is resumed. Subjects with persistent Grade 1 pneumonitis after completion of a steroid taper over at least 1 month may be eligible for retreatment if investigator allows.
- Drug-related endocrinopathies adequately controlled with only physiologic hormone replacement may resume treatment if investigator allows.

If the criteria to resume treatment are met, the subject should restart treatment at the next scheduled timepoint per protocol. However, if the treatment is delayed past the next scheduled timepoint per protocol, the next scheduled timepoint will be delayed until dosing resumes.

If treatment is delayed or interrupted for > 6 weeks, the subject must be permanently discontinued from study therapy, except as specified in discontinuation section.

6.1.3 Management Algorithms

Immuno-oncology (I-O) agents are associated with AEs that can differ in severity and duration than AEs caused by other therapeutic classes. Nivolumab and ipilimumab are considered as an immuno-oncology agent in this protocol. Early recognition and management of AEs associated with immuno-oncology agents may mitigate severe toxicity. Management algorithms have been developed to assist investigators in assessing and managing the following groups of AEs:

Gastrointestinal, Renal, Pulmonary, Hepatic, Endocrinopathies, Skin, Neurological, Myocarditits.

For subjects expected to require more than 4 weeks of corticosteroids or other immunosuppressants to manage an AE, consider recommendations provided in the algorithms. These algorithms are found in the IB for Nivolumab and Ipilimumab. The guidance provided in these algorithms should not replace the Investigator's medical judgment but should complement it.

6.1.4 Discontinuation Criteria

Nivolumab and/or ipilimumab should be permanently discontinued for the following:

- Any Grade 2 drug-related uveitis or eye pain or blurred vision that does not respond to topical therapy and does not improve to Grade 1 severity within the re-treatment period OR requires systemic treatment
- Any Grade 3 non-skin, drug-related adverse event lasting > 7 days, with the following exceptions for drug-related laboratory abnormalities, uveitis, pneumonitis, bronchospasm, hypersensitivity reactions, and infusion reactions, and endocrinopathies:
    - Grade 3 drug-related uveitis, pneumonitis, bronchospasm, hypersensitivity reaction, or infusion reaction of any duration requires discontinuation
    - Grade 3 drug-related endocrinopathies adequately controlled with only physiologic hormone replacement do not require discontinuation
    - Grade 3 drug-related laboratory abnormalities do not require treatment discontinuation except those noted below
        - Grade 3 drug-related thrombocytopenia > 7 days or associated with bleeding requires discontinuation
        - Any drug-related liver function test (LFT) abnormality that meets the following criteria require discontinuation:
            - AST or ALT > 8 x ULN
            - Total bilirubin > 5 x ULN
            - <u>Concurrent</u> AST or ALT > 3 x ULN and total bilirubin > 2 x ULN
- Any Grade 4 drug-related adverse event or laboratory abnormality, except for the following events which do not require discontinuation:

  - Isolated Grade 4 amylase or lipase abnormalities that are not associated with symptoms or clinical manifestations of pancreatitis and decrease to < Grade 4 within 1 week of onset.
  - Isolated Grade 4 electrolyte imbalances/abnormalities that are not associated with clinical sequelae and are corrected with supplementation/appropriate management within 72 hours of their onset
  - Grade 4 lymphopenia or leucopenia
  - Grade 4 drug-related endocrinopathy adverse events, such as adrenal insufficiency, ACTH deficiency, hyper- or hypothyroidism, or glucose intolerance, which resolve or are adequately controlled with physiologic hormone replacement (corticosteroids, thyroid hormones) or glucose-controlling agents, respectively, may not require discontinuation after discussion with and approval from the Investigator.

- Any dosing interruption lasting > 6 weeks with the following exceptions:
  - Dosing delays or interruptions to allow for prolonged steroid tapers to manage drug-related adverse events are allowed. Prior to re-initiating treatment in a subject with a dosing interruption lasting > 6 weeks, the Coordinating Investigator must be consulted. Tumor assessments should continue as per protocol even if dosing is interrupted or delayed
  - Dosing interruptions or delays lasting > 6 weeks that occur for non-drug-related reasons may be allowed if approved by the Coordinating Investigator. Prior to re-initiating treatment in a subject with a dosing interruption lasting > 6 weeks, the Coordinating Investigator must be consulted. Tumor assessments should continue as per protocol even if dosing is interrupted

- Any adverse event, laboratory abnormality, or intercurrent illness which, in the judgment of the Investigator, presents a substantial clinical risk to the subject with continued nivolumab and ipilimumab dosing

According to investigators´ decision, Ipilimumab can be discontinued and treatment can be continued with Nivolumab alone in case of drug-related AEs not fulfilling the criteria for discontinuation of both drugs but would appear inacceptable for the investigators.

6.1.5 Dose Modification Criteria for Docetaxel, Oxaliplatin-Plus-Fluoropyrimidine Treatment

Dose modifications of docetaxel, oxaliplatin, leucovorin and fluorouracil are permitted according to local standards or local package inserts.

- Doses of any study drug omitted for toxicity are not replaced or restored; instead, the patient should resume the planned treatment cycles. Supportive care (for example, colony-stimulating factors [CSFs], blood and blood products, etc. can be administered in accordance with the latest American Society of Clinical Oncology (ASCO) or other equivalent guidelines.
- Dose modification, for non-serious and non-life-threatening toxicities like alopecia, altered

taste or nail changes may not be required and the final decision is left to the discretion of the treating investigator.

- In situations where concomitant toxicities of varying severity exist, dose modification will be tailored for the toxicity with highest CTCAE grading.
- If there is a delay or modification in administration of study drug(s) due to toxicity, treatment with the other study agent(s) should continue as scheduled. If clinically appropriate, the investigator can delay all treatment components up to a maximum of 7 days to allow synchronized administration of all agents.

If toxicity related to any component of chemotherapy does not resolve in the same treatment cycle, the administration of that component can be delayed up to 6 weeks. If the toxicity does not resolve within 6 weeks, that component will be discontinued unless it is determined by the treating investigator that the patient might benefit from continuation of the component.

Reasons for dose modifications of FOLFOX and recommended dose modifications are provided in Table 6 and 7.

Table 6: Recommended Dose Modifications of FOLFOX

		**Dose Modification**	
**Drug**	**Starting Dose**	Dose Level - 1	Dose Level - 2
Oxaliplatin	85 mg/m^2	70 mg/m^2	50 mg/m^2
5-FU	Bolus 5-FU: 400 mg/m^2<br>Leucovorin: 400 mg/m^2<br>Infusion 5-FU: 2400 mg/m^2 /44 hours	Bolus 5-FU: 300 mg/m^2<br>Leucovorin: 300 mg/m^2<br>Infusion 5-FU: 2000 mg/m^2 /44 hours	Bolus 5-FU: 200 mg/m^2<br>Leucovorin: 200 mg/m^2<br>Infusion 5-FU: 1600 mg/m^2 /44 hours

5-FU: 5-fluorouracil

Table 7: Dose Modifications of FOLFOX

Toxicity	Definition	During a course of therapy	Dose adjustment for next treatments
Neutropenia	Grade 3 or greater	Interrupt until resolved to ≤ Grade 2	Dose level -1<br>*If treatment delayed for 4 consecutive weeks, discontinue all treatment
Thrombocytopenia	Grade 2	Interrupt until resolved to ≤ Grade 1	Dose level -1<br>*If Grade 2 persists > 7 days, oxaliplatin reduced by 2 dose levels when platelets improve to Grade 1
	Grade 3	Interrupt until resolved to ≤ Grade 1	Dose level -1<br>*If Grade 3 persists > 7 days, oxaliplatin reduced by 2 dose levels when platelets improve to Grade 1
	Grade 4	Interrupt until resolved to ≤ Grade 1	Dose level -2<br>*If Grade 4 persists > 7 days, oxaliplatin reduced by 2 dose levels when platelets improve to Grade 1
Neurologic toxicity	Grade 2 peripheral sensory neuropathy	Interrupt until resolved to ≤ Grade 1	Oxaliplatin dose -1<br>Continue 5-FU and leucovorin<br>*If oxaliplatin delayed for neurologic toxicityfor 4 consecutive weeks, discontiue oxaliplatin, continue 5-FU and leucovorin
	Grade 3 or greater<br>peripheral sensory neuropathy	Discontinue oxaliplatin	Continue 5-FU and leucovorin
Gastrointestinal toxicities	Grade 2 or greater diarrhea	Interrupt until resolved to ≤ Grade 1	Dose level -1<br>If dose delayed for diarrhea for 4 consecutive weeks, discontinue all treatment

Reasons for dose modifications of FLOT (Arm C) and recommended dose modifications are provided for hematologic, renal and other toxicity:

Patients who experience febrile neutropenia (despite the use of G-CSF), or thrombocytopenia causing bleeding, or any other hematological dose limiting toxicities (DLT, investigator decision) will receive a dose reduction of docetaxel and oxaliplatin to 75% of the initial dose. Subsequent dose limiting toxicities lead to a further dose reduction to 50% of the initial dose level. If DLTs reoccur at the 50% dose level, the investigator may remove one or both of the drugs (investigator decision).

If a creatinine clearance of < 30 ml/min is measured, oxaliplatin treatment will be stopped. 5-FU and docetaxel administration can be continued under consideration of the recommendations given in the respective SmPCs (Fachinformation).

If non-hematologic toxicities ≥ grade 3 appear, the dose of the chemotherapeutic agent most likely responsible for the observed toxicity should be reduced to 75% of the initial dose (for all further cycles). This procedure may apply to grade 2 toxicities upon investigator decision.

In case of repeated toxicity, a further dose reduction to 50% of the initial dose should be performed. If the toxicity re-occurs at the 50% dose level, the investigator should remove the relevant drug(s) or stop the whole treatment.

Each of the chemotherapeutic agents administered in the course of this study are market approved in Germany and all participating investigators will have broad experience with these medications. Therefore, investigators are permitted to deviate from the recommendations given above in reasonable cases. For example, investigators are allowed to reduce or remove one or more components to the FLOT regimen in accordance with their own local guidelines if they feel that this is in the best interest of the patient.

For toxicities not listed above, dose modifications are permitted per local standards.

Subjects may also discontinue oxaliplatin following multiple cycles if, in the investigator’s judgment, cumulative toxicity is likely to increase over time and become problematic.

6.1.6 Discontinuation Criteria for FOLFOX

Discontinuation of FOLFOX chemotherapy will be based on local criteria and has to be documented in the eCRF.

6.1.7 Discontinuation Criteria for FLOT

Discontinuation of FLOT chemotherapy will be based on local criteria and has to be documented in the eCRF.

6.1.8 Treatment Infusion Reactions related to Nivolumab or Ipilimumab

Since nivolumab and ipilimumab contains only human immunoglobulin protein sequences, it is unlikely to be immunogenic and induce infusion or hypersensitivity reactions. However, if such a reaction were to occur, it might manifest with fever, chills, rigors, headache, rash, pruritis, arthralgias, hypo- or hypertension, bronchospasm, or other symptoms of allergic-like reactions.

All Grade 3 or 4 infusion reactions should be reported as an SAE if criteria are met. Infusion reactions should be graded according to NCI CTCAE, version 4.03 (Appendix C) guidelines.

Treatment recommendations are provided below and may be modified based on local treatment standards and guidelines as appropriate:

For Grade 1 symptoms: (Mild reaction; infusion interruption not indicated; intervention not indicated)

Remain at bedside and monitor subject until recovery from symptoms. The following prophylactic premedications are recommended for future infusions: diphenhydramine 50 mg (or equivalent) and/or paracetamol 325 to 1000 mg (acetaminophen) at least 30 minutes before nivolumab and ipilimumab administrations.

For Grade 2 symptoms: (Moderate reaction requires therapy or infusion interruption but responds promptly to symptomatic treatment [eg, antihistamines, non-steroidal anti inflammatory drugs, narcotics, corticosteroids, bronchodilators, IV fluids]; prophylactic medications indicated for 24 hours).

Stop the nivolumab or ipilimumab infusion, begin an IV infusion of normal saline, and treat the subject with diphenhydramine 50 mg IV (or equivalent) and/or paracetamol 325 to 1000 mg (acetaminophen); remain at bedside and monitor subject until resolution of symptoms. Corticosteroid or bronchodilator therapy may also be administered as appropriate. If the infusion is interrupted, then restart the infusion at 50% of the original infusion rate when symptoms resolve; if no further complications ensue after 30 minutes, the rate may be increased to 100% of the original infusion rate. Monitor subject closely. If symptoms recur then no further, nivolumab and ipilimumab will be administered at that visit. Administer diphenhydramine 50 mg IV, and remain at bedside and monitor the subject until resolution of symptoms. The amount of study drug infused must be recorded on the electronic case report form (eCRF). The following prophylactic premedications are recommended for future infusions: diphenhydramine 50 mg (or equivalent) and/or paracetamol 325 to 1000 mg (acetaminophen) should be administered at least 30 minutes before additional nivolumab and ipilimumab administrations. If necessary, corticosteroids (recommended dose: up to 25 mg of IV hydrocortisone or equivalent) may be used.

For Grade 3 or Grade 4 symptoms: (Severe reaction, Grade 3: prolonged [ie, not rapidly responsive to symptomatic medication and/or brief interruption of infusion]; recurrence of symptoms following initial improvement; hospitalization indicated for other clinical sequelae [eg, renal impairment, pulmonary infiltrates]). Grade 4: (life threatening; pressor or ventilatory support indicated).

Immediately discontinue infusion of nivolumab or ipilimumab. Begin an IV infusion of normal saline, and treat the subject as follows. Recommend bronchodilators, epinephrine 0.2 to 1 mg of a 1:1,000 solution for subcutaneous administration or 0.1 to 0.25 mg of a 1:10,000 solution injected slowly for IV administration, and/or diphenhydramine 50 mg IV with methylprednisolone 100 mg IV (or equivalent), as needed. Subject should be monitored until the investigator is comfortable that the symptoms will not recur. Nivolumab or ipilimumab will be permanently discontinued. Investigators should follow their institutional guidelines for the treatment of anaphylaxis. Remain at bedside and monitor subject until recovery from symptoms.

In the case of late-occurring hypersensitivity symptoms (eg, appearance of a localized or generalized pruritis within 1 week after treatment), symptomatic treatment may be given (eg, oral antihistamine, or corticosteroids).

7. Criteria of Evaluation

7.1 Progression Free Survival (PFS)

Time from randomization/enrolment (Arm C) to the date of first observed disease progression (investigator assessment according to RECIST 1.1) or death from any cause. Subjects who die without a reported prior progression will be considered to have progressed on the date of their death. Subjects who did not progress or die will be censored on the date of their last evaluable tumor assessment. Subjects who did not have any on study tumor assessments and did not die will be censored on the date they were registered. Subjects who started any subsequent anti-cancer therapy without a prior reported progression will be censored at the last evaluable tumor assessment prior to or on the date of initiation of the subsequent anti-cancer therapy.

7.2 Overall Response Rate (ORR)

Overall response rate will be assessed according to RECIST v1.1 (refer to appendix D). Overall response rate will be defined as the proportion of randomized/enrolled subjects with best response of complete or partial reponse.

7.3 Overall Survival (OS)

Overall survival will be determined as time from the randomization/enrolment date to the date of death. A subject who has not died will be censored at last known date alive.

7.4 Safety Endpoints

Safety assessments will include physical examinations including performance status (ECOG), clinical laboratory profile and adverse events.

All observed toxicities and side effects will be graded according to NCI CTCAE v4.03 for all patients and the degree of association of each with the procedure assessed and summarized. Treatment related serious adverse events rate (SAE) will be determined.

7.5 Quality of life assessment (EORTC QLQ-C30)

Quality of life will be assessed using the EORTC QLQ-C30 questionnaire at baseline, during treatment after every 4th cycle (= approximately every 8 weeks; prior to imaging) and after EOT every 3 months until progression.

8. Translational research

8.1 Translational research projects

The following translational research is currently planned, but may be adapted taking into account new research data:

- FFPE tissue will be centrally tested for target gene expression and immune cell composition by molecular quantitation of PD-L1, PD-L2, PD1, CTLA4, CD80, CD3, CD4, CD8, FOXP3, CD68, IGKC, CXCL9, CXCL10, CXCL13 and correlated with clinical efficacy.
- In addition FFPE tissue will be centrally tested for MSI, EBV, ERBB2, MMP7, MMP9, RTK signalling pathway members and mutation status (kRAS, TP53).

Thus, the tumor block or slices will be obtained at baseline. EDTA blood will be collected once.

8.2 Sampling time points and materials

Paraffin-embedded tumor-tissue for PD-L1 assessment and translational research will be obtained at baseline. Centers may send either a paraffin block or slices. If slices are sent, please send 1 x HE stained slice, 4 x 5 µm unstained tissue slices on glass slides..

FFPE tissue slices will be sent to :

Institut für Klinische Krebsforschung IKF GmbH
at Krankenhaus Nordwest
Steinbacher Hohl 2-26
60488 Frankfurt
Germany

Blood (2 EDTA tubes a 2 mL) will be collected once and should be sent together with the related, completely filled forms via postal service (without cooling) to:

Dr. Ralph Wirtz
STRATIFYER

Molecular Pathology GmbH
Werthmannstr. 1c
50935 Köln
Germany

8.3 Sample storage and archiving

Residual material from the blood samples and parts of the tissue blocks will be destroyed or archived at the authorized institution for a period of at least 15 years after the end of the study for further investigations regarding the disease. After testing has been completed the remainder of the sample will be kept for any follow-up investigation for an indefinite period. If necessary, tissue samples can be returned to the responsible pathologist and be archived there.

If consent to the planned examinations as described within the informed consent form is revoked by the patient, the blood and tissue samples will not be used for further examinations. Remaining blood samples will be destroyed, remaining tissue samples will be sent back to the responsible pathologist for archiving

9. Assessment of Adverse Events & Safety Reporting

Adverse event reporting summary

It is the responsibility of the investigators to record all adverse events in the eCRF. Any serious adverse event (SAE, irrespective of suspected causal relationship) which occurs after the patient has given written informed consent and up to the end of the safety follow-up period (100 days after last dose in all arms) must be reported in writing within 24 hours after site awareness to the sponsor/sponsor representative.

Reports have to be sent via fax or email to:

Institut für Klinische Krebsforschung IKF GmbH
at Krankenhaus Nordwest
Steinbacher Hohl 2-26
60488 Frankfurt/Main
Germany
Fax: +49 / (0)69 / 7601-3655
Email: sae@ikf-khnw.de

9.1 Reference safety documents

The current versions of the Investigators' Brochure (IB) of nivolumab and ipilimumab as well as a current SmPC of Docetaxel, Oxliplatin, 5-Fluorouracil and Leucovorin will be used as reference documents and will be provided to the investigators in the Investigator's Site File.

9.2 Adverse Events Definitions

9.2.1 Adverse Event

An adverse event (AE) is defined in the International Conference on Harmonization (ICH) Guideline for Good Clinical Practice as "any untoward medical occurrence in a patient or clinical investigation subject administered a pharmaceutical product and which does not necessarily have a causal relationship with this treatment." (ICH E6: section 1.2).

An adverse event can therefore be any unfavorable and unintended sign (including an abnormal laboratory finding, for example), symptom, or disease temporally associated with the use of a medicinal (investigational) product, whether or not considered related to the medicinal (investigational) product.

Worsening of a pre-existing medical condition (e.g. diabetes, migraine headaches, gout) should be considered an adverse event if there is either an increase in severity, frequency, or an association with significantly worse outcomes.

Interventions for pre-treatment conditions (e.g. elective cosmetic surgery) or medical procedures that were planned before study enrolment are not considered adverse events.

9.2.2 Serious Adverse Event

A serious adverse event (SAE) is defined as any untoward medical occurrence (adverse event) that at any dose:

- results in death,
- is life-threatening (subject was at immediate risk of death at the time of the event),
- requires in-patient hospitalization or prolongation of existing hospitalization,
- results in persistent or significant disability/incapacity,
- is a congenital anomaly / birth defect or,
- any other significant medical condition.

A hospitalization meeting the regulatory definition for "serious" is any inpatient hospital admission that includes a minimum of an overnight stay in a health care facility. Any adverse event that does not meet one of the definitions of serious i.e. important medical events that may not be immediately life-threatening or result in death or hospitalization but may jeopardize the patient or may require interventions to prevent one of the other outcomes listed above (e.g. emergency room visit, outpatient surgery, or requires urgent investigation) may be considered by the investigator to meet the "other significant medical condition" criterion for classification as a serious adverse event. Examples include allergic bronchospasm, convulsions, and blood dyscrasias.

Hospitalization for performing of protocol-required procedures or administration of study treatment or hospitalizations for procedures planned prior to study start and elective hospitalizations are not classified as an SAE.

Progression of the underlying malignant disease and symptoms caused by progression of the underlying tumor disease need not to be reported as SAE in this protocol, unless progression or symptoms of progression are assessed as causally related to study medication.

9.2.3 Other reportable events

The following events are reportable and must be handeled as SAEs:

- Is a new cancer (that is not a condition of the study);
- overdose: An overdose is defined as a subject receiving a dose of IMP in excess of that specified in the Investigator's Brochures, unless otherwise specified in this protocol.
- pregnancy
- Transmission of an infectious agent via medicinal product
- Drug induced liver injury defined as follows:
    - ALT or AST elevation > 3 times upper limit of normal (ULN)

    AND

    - Total bilirubin > 2 times ULN, without initial findings of cholestasis (elevated serum alkaline phosphatase),

    AND

    - No other immediately apparent possible causes of AT elevation and hyperbilirubinemia, including, but not limited to, viral hepatitis, pre-existing chronic or acute liver disease, or the administration of other drug(s) known to be hepatotoxic.

9.2.4 Unexpected Adverse Event

An unexpected adverse event is any adverse drug event, the specificity or severity of which is not consistent with the current version of the Investigators' Brochure of the IMPs or the respective SmPC as applicable. Also, reports which add significant information on specificity or severity of a known, already documented adverse event constitute unexpected adverse events. An event more specific or more severe than described in the current version of the Investigators' Brochure of the IMPs or the respective SmPC (as applicable) would be considered "unexpected".

A suspected unexpected serious adverse reaction (SUSAR) is a serious adverse reaction, the nature, or severity of which is not consistent with the applicable safety reference document (current IB or SmPC). All suspected adverse reactions related to the study medication which

occur in the concerned trial and that are both unexpected and serious (SUSARs) are subject to expedited safety reporting.

9.3 Assessment of relationship - Adverse drug reaction

The causal relationship to study drug is determined by a physician and should be used to assess all adverse events (AE). The causal relationship can be one of the following:

- **Related:** There is a reasonable causal relationship between study drug administration and the AE.
- **Not related:** There is not a reasonable causal relationship between study drug administration and the AE.

The term "reasonable causal relationship" means there is evidence to suggest a causal relationship.

In case of a missing causality assessment in the eCRF or SAE reporting form, the event will be regarded as "probably related" unless further specified.

A serious ADR (SADR) is an adverse drug reaction that meets the definition of a serious event (provided above).

9.4 Assessment of severity

Intensity of adverse events will be graded using the National Cancer Institute Common Terminology Criteria for Adverse Events (CTCAE), version 4.03.

If an adverse event occurs which is not contained in the CTCAE version 4.03, the five-point scale below will be used.

Grade 1:	Mild; asymptomatic or mild symptoms; clinical or diagnostic observations only; intervention not indicated
Grade 2:	Moderate: minimal, local or noninvasive intervention indicated; limiting age-appropriate instrumental ADL
Grade 3:	Severe or medically significant but not immediately life-threatening; hospitalization or prolongation of hospitalization indicated; disabling; limiting self-care ADL
Grade 4:	Life-threatening consequences; urgent intervention indicated
Grade 5:	Death related to AE

9.5 Safety recording and reporting requirements

9.5.1 Recording periods

- *Non-serious* and *serious adverse events* are recorded continuously from time of signed informed consent until 100 days after last dose of IMP.
- *Other reportable events* are continuously recorded from time of signed informed consent until 100 days after last dose of IMP.
- Pregnancies occurring in a study subject are recorded from time of signed informed consent until **5 months** after last dose of IMP. Pregnancies occurring in a partner of a study subject are recorded from time of signed informed consent until **7 months** after last dose of IMP.

9.5.2 Recording and Reporting requirements

Adverse events: The investigator is responsible for ensuring that all adverse events observed by the investigator or reported by patient are properly captured in the patients' medical records.

Adverse events will be recorded in the AE page of eCRF using a recognized medical term or diagnosis that accurately reflects the event. Adverse events will be assessed by the investigator for severity, relationship to the investigational product, possible etiologies, and whether the event meets criteria of an SAE or other reportable event (as per section 9.2.3) and therefore requires expedited reporting.

The following variables will be collected for each AE:

- AE (verbatim)
- The date when the AE started and stopped
- Changes in NCI CTCAE grade and the maximum CTC grade attained
- Whether the AE is serious or not
- Investigator causality rating against IMPs (nivolumab, ipilimumab, chemotherapy, yes or no)
- Action taken with regard to IMP:
    - none
    - study drug temporarily interrupted
    - study drug dose modifications
    - study drug permanently discontinued.
- Outcome:
    - recovered/resolved
    - recovered/resolved with sequelae
    - not recovered/not resolved
    - fatal
    - unknown (only applicable if patient is lost to follow-up);

In addition, the following variables will be collected for SAEs as applicable:

- Date AE met criteria for serious AE
- Date Investigator became aware of serious AE
- Seriousness criterion
- Date of hospitalization
- Date of discharge
- Probable cause of death
- Date of death
- Autopsy performed
- Description of AE
- Causality assessment in relation to other Study procedure(s)

Serious adverse events:

- For each patient any adverse event or abnormal laboratory test value that is serious occurring during the course of the study must be reported immediately (within 24 hours / GCP-V § 12(4)) after awareness to the sponsor/sponsor representative via fax utilizing a completed SAE Report Form.

- Serious Adverse Events that are **unexpected** and **considered related** to IMP and occur **after the completion of the trial** should be reported to the sponsor/sponsor representative within one working day [ICH E2A III.E.3].

Other reportable events:

- For each study subject any adverse event that fulfils the criteria in section 9.2.3 occurring during the course of the study must be reported immediately (within 24 hours) after awareness to the sponsor or his representative (CRO) via fax utilizing a completed SAE Report Form.
- Overdose: Any overdose of a study subject with any of the IMPs, with or without associated AEs/SAEs, must be reported immediately (within 24 hours) after awareness to the sponsor/sponsor representative via fax utilizing a completed SAE Report Form.
- If the overdose results in an AE, the AE must also be recorded as an AE. Overdose does not automatically make an AE serious, but if the consequences of the overdose are serious, for example death or hospitalization, the event is serious and must be recorded and **reported** as an SAE.

Pregnancies:

- Pregnancies occurring in a study subject or partner of a study subject are reported **within 24 hours** of knowledge of the event to the sponsor/sponsor representative using the Pregnancy Report Form.

Follow-up information regarding the course of the pregnancy, including perinatal and neonatal outcome and, where applicable, offspring information must be reported on the Pregnancy Surveillance Form. See section 9.6.6 for further details.

- If an SAE is experienced in addition to or related to the pregnancy e.g. an induced or spontaneous abortion, also an SAE Report has to be sent to the sponsor within 24 hours of first knowledge.

Abnormal laboratory results:

In general it is the investigator's responsibility to review all abnormal laboratory results and to determine if a given value represents a clinically significant change compared to previously obtained values and results in an Adverse Event or not.

- Abnormal laboratory test results will be recorded on the laboratory results pages of the eCRF. Laboratory-test-value abnormalities should additionally be considered an AE in case they are:
    1. Accompanied by clinical symptoms
    2. Leading to a change in study medication (e.g. dose modification, interruption or permanent discontinuation)
    3. Requiring a change in concomitant therapy (e.g. addition of, interruption of, discontinuation of, or any other change in a concomitant medication, therapy or treatment)
- Any laboratory result abnormality fulfilling the criteria for a serious adverse event (SAE) should be reported as such, in addition to being recorded as an adverse event in the eCRF.

9.5.3 Sponsor obligations

The sponsor, sponsor representative and the CRO will ensure compliance with all regulatory reporting requirements including the notification of the appropriate Ethics Committees, Competent Authority and participating investigators of all serious adverse events occurring at the sites in accordance with national law, ICH Good Clinical Practice and European / EMA requirements.

- A sponsor representative (e.g. CRO or medical expert) will medically review all SAE reports and perform the expectedness assessment.
- A sponsor representative (e.g. CRO) will forward SAE, other reportable events and pregnancy reports within one working day to the Coordinating Investigator (CI/LKP) and BMS.
- Every SAE, being assessed by either the investigator or the sponsor/sponsor representative as suspected to be related to IMP und assessed as being either unexpected or unexpected with regard to outcome or severity of the event will be reported by the

sponsor/sponsor representative as SUSAR to the competent authority, responsible ethics committee and investigators of the trial in line with the national regulations in effect (German drug law [AMG] and GCP-V § 13).

- Fatal or life-threatening SUSARs must be reported as soon as possible, but no later than 7 days; further important information to these cases may be reported as follow-up within additional 8 days. All others SUSARs have to be reported no later than 15 days. BMS will be notified in parallel.
- Also all adverse events which can change the benefit-risk ratio of the study drugs or otherwise fulfil the criteria outlined in GCP-V §13 Abs.4 have to be handled/reported as SUSARs. BMS will be notified in parallel.

9.6 Handling of Safety Parameters

9.6.1 Adverse events

Adverse events can be spontaneously reported or elicited during open-ended questioning, examination, or evaluation of a subject. (In order to prevent reporting bias, subjects should not be questioned regarding the specific occurrence of one or more AEs.)

It will be left to the investigator's clinical judgment to determine whether an adverse event is related and of sufficient severity to require the subject's removal from treatment or from the study. A subject may also voluntarily withdraw from treatment due to what he or she perceives as an intolerable adverse event. If either of these situations arises, the subject should be strongly encouraged to undergo an end-of-treatment assessment and be under medical supervision until symptoms cease or the condition becomes stable.

9.6.2 Treatment and Follow-up of Adverse Events

During the course of the study all AEs and SAEs should be proactively followed up for each subject. Every effort should be made to obtain a resolution for all events, even if the events continue after discontinuation/study completion. The investigator is responsible for following all SAEs until resolution, until the subject returns to baseline status, or until the condition has stabilized with the expectation that it will remain chronic, even if this extends beyond study participation.

9.6.3 Follow-up of Abnormal Laboratory Test Values

In the event of unexplained abnormal laboratory test values, the tests should be repeated immediately and followed up until they have returned to the normal range and/or an adequate explanation of the abnormality is found. If a clear explanation is established it should be recorded on the eCRF.

9.6.4 Overdose

The investigator will use clinical judgment to treat any overdose.

9.6.5 Drug induced liver injury

Drug-induced liver injury (DILI) is under constant surveillance by sponsors and regulators and is considered a protocol-specified adverse event (Other reportable event). Timely detection, evaluation, and follow-up of laboratory alterations of selected liver laboratory parameters to distinguish an effect of the investigational drug from other causes are important for patient safety and for the medical and scientific interpretation of the finding.

Wherever possible, timely confirmation of initial liver-related laboratory abnormalities should occur prior to the reporting of a potential DILI event.

Study subjects showing laboratory abnormalities as defined in section 9.2.3 need to be followed up until the protocol specific retreatment criteria have been met and according to 6.1.2 of this clinical trial protocol.

9.6.6 Pregnancies and contraception

Reproductive status

For this trial, male subjects will be considered to be of non-reproductive potential if they have azoospermia (whether due to having had a vasectomy or due to an underlying medical condition).

Female subjects will be considered of non-reproductive potential if they are either:

(1) postmenopausal (defined as at least 12 months with no menses without an alternative medical cause; in women < 45 years of age a high follicle stimulating hormone (FSH) level in the postmenopausal range may be used to confirm a post-menopausal state in women not using hormonal contraception or hormonal replacement therapy. In the absence of 12 months of amenorrhea, a single FSH measurement is insufficient.);

OR

(2) have had a hysterectomy and/or bilateral oophorectomy, bilateral salpingectomy or bilateral tubal ligation/occlusion, at least 6 weeks prior to screening;

OR

(3) has a congenital or acquired condition that prevents childbearing.

Counseling of study subjects and partners:

Subjects should be informed that taking the study medication may involve unknown risks to the fetus (unborn baby) if pregnancy were to occur during the study. In order to participate in the study subjects of childbearing potential must adhere to the contraception requirements (see section 4.2.1). If there is any question that a subject of childbearing potential will not reliably

comply with the requirements for contraception, that subject should not be entered into the study.

- Maternal exposure: Women of childbearing potential (WOCBP) must use appropriate method(s) of contraception. *WOCBP should use an adequate method to avoid pregnancy for 5 months after the last dose of nivolumab and ipilimumab*. A female patient must be instructed to immediately inform the investigator if she becomes pregnant during the study. Monitoring of the patient should continue until conclusion of the pregnancy.
- Paternal exposure: Men who are sexually active with WOCBP must use any contraceptive method with a failure rate of less than 1% per year. Men receiving IMPs and who are sexually active with WOCBP will be instructed to adhere to contraception for a period of 7 months after the last dose of investigational products. Male subjects must refrain from donating sperm during the study and for 7 months after the last dose of nivolumab and ipilimumab. A male study subject must be instructed to immediately inform the investigator if a pregnancy occurs in his partner during the study and up to 7 months after last dose of IMP.

Pregnancies

Pregnancy itself, or pregnancy of a subject's partner, is not regarded as an adverse event unless there is a suspicion that the investigational product under study may have interfered with the effectiveness of a contraceptive medication. Congenital abnormalities/birth defects and spontaneous miscarriages should be reported and handled as SAEs. Elective abortions without complications should not be handled as AEs. The outcome of any conception occurring from the date of the first dose until 5 months (female subjects) or 7 month (partners of male subjects) after the last dose (spontaneous miscarriage, elective termination, ectopic pregnancy, normal birth or congenital abnormality) should be followed up and documented even if the subject was withdrawn from the study.

Subjects who become pregnant during the study period must not receive additional doses of investigational product but will not be withdrawn from the study until the necessary safety follow-up has been completed. The investigator should counsel the subject; discuss the risks of continuing the pregnancy, and possible effects on the fetus. The pregnancy will be followed for outcome of the mother and child (including any premature terminations) and should be reported to the sponsor/sponsor representative, which will notify BMS or designee after outcome.

Pregnancy of a subject's partner is not considered to be an AE. However, the outcome of all pregnancies (spontaneous miscarriage, elective termination, ectopic pregnancy, normal birth, or congenital abnormality) occurring from the date of the informed consent 7 month after the last dose should, if possible, be followed up and documented. The investigator should counsel the subject's partner; discuss the risks of continuing the pregnancy, and possible effects on the fetus.

Where a report of pregnancy is received, prior to obtaining information about the pregnancy, the Investigator must obtain the consent of the subject's partner. Sponsor will provide a partner

ICF in line with local procedures and submit it to the relevant Ethics Committees (ECs)/Institutional Review Boards (IRBs) prior to use.

9.6.7 Adverse Drug reactions with Concomitant Medication

The investigators must be aware that for all concomitant medications the regulations of post marketing reporting for suspected adverse drug reactions apply, i.e. reporting to the marketing authorization holder or the local regulatory bodies.

9.7 Independent Data Monitoring Committee (IDMC)

An independent data monitoring committee will follow the progress of the clinical trial, evaluate the safety and primary efficacy parameters and will propose changes, ending or continuing of the trial to the sponsor. A separate IDMC charta will be developed and submitted to competent authority and EC. The planned interim safety analysis will be performed by the IDMC with the data collected and prepared by the sponsor.

10. Data Analysis and Statistical Considerations

10.1 Sample Size Calculation

The present trial is designed as a randomized phase II study followed by a non-randomized arm, which aims to estimate the therapeutic efficacy of three experimental regimen.

Arm A vs. Arm B:

PFS analysed according to the ITT principle for patients treated with mFOLFOX plus Nivolumab plus Ipilimumab (Arm A) vs. patients treated with mFOLFOX alone (Arm B) is the primary efficacy endpoint. The efficacy assumptions are derived from historical data.

The expected median PFS in the standard arm is 5.5 months; the expected median PFS in the experimental arm is 8.5 months. We, therefore, hypothesize that the experimental therapy is associated with clinically relevant improvement according to a HR of 0.68 (which would reflect the medians mention above). In the frame of a phase II testing, the use of a one-sided significance level of 10% is justified. Based on this, 118 randomized subjects (59 in the control and 59 in the experimental treatment group) will be enrolled to provide 80% power for detecting an average HR of 0.68 using the log rank test at a one-sided type I error of 10% and assuming an exponentially distriputed 5% drop out rate. The sample size calculation is follow up trigerred and is performed under the assumption of a 2 year enrollment periode followed by a 1 year follow up period starting with last patient-in. This leads to a minimum follow up time of 3 years, which is regarded sufficient enough in a patients population with an expected median survival of 9 to 11 months.

All eligible patients will be randomized in a 1:1 ratio into Arm A and B for the first 118 patients and in a 1:2 ratio into Arm A1 and A2 in the subsequent 89 patients according to the following stratification criteria:

- ECOG PS (0 vs 1)
- Tumor status (prior resection vs. no prior resection)

Arm A1 vs. Arm A2:
To evaluate if a sequential treatment of mFOLFOX plus Nivolumab plus Ipilimumab (Arm A2) is less toxic but equally effective as parallel treatment of mFOLFOX plus Nivolumab plus Ipilimumab (Arm A and A1) 57 patients are needed using a one-stage Fleming design (Fleming 1982) with following assumptions:

- The sequential therapy would be rated as unacceptable, if the actual PFS rate at 6 months (PFS@6) was only 47% or lower (corresponding to the median PFS of Arm B of 5.5 months)
- The sequential therapy would be considered to be a promising candidate for further development, if the true PFS@6 amounted to 61% or higher (corresponding to the expected median PFS of Arm A of 8.5 months)
- Probability to accept the sequential therapy as effective, in spite of a true PFS@6 of <47%: 10% (type I error)
- Probability to reject the sequential therapy as ineffective (<47%), although the true PFS@6 is promising (>61%): 20% (type II error, corresponding to a power of 80%)

Allowing for two non-informative drop-outs, 59 patients have to be recruited into Arm A2. 30 patients are to be allocated to the reference arm A1, according to the 1:2 randomization. The same stratification factors (ECOG PS and tumor status) as in the randomization of arms A and B are applied.
The final conclusion for the sequential treatment will depend on the definite PFS rate and its confidence interval, the respective findings in the reference arm, as well as the information on type, frequency and severity of toxicities.

Arm C:
Based on emerging data from recent phase III trials, immune checkpoint inhibitors such as Nivolumab and Pembrolizumab added to the doublet chemotherapy prolong OS and PFS, but the extent of improvement regarding PFS is smaller than expected and if administered as monotherapy, there is a an increased early mortality (crossing survival curves) with the checkpoint inhibitors as compared with chemotherapy (Tabernero et al. 2019; Janjigian et al. 2021; Kato et al. 2020). This is most likely explained by the fact that patients need time to establish antitumoral immunity, while some patients with aggressive disease experience early disease progression and death. This provides a rationale to test whether the intensification of chemotherapy using a triplet (mFOLFOX plus Docetaxel = FLOT) instead of the doublet, reducing immunotherapy to Nivolumab instead of Nivolumab and Ipilimumab would be more beneficial and safer. Therefore, Arm C is designed to evaluate the efficacy of the combination of FLOT plus Nivolumab in the same patient group and descriptively compare this treatment

with mFOLFOX plus Nivolumab plus Ipilimumab (Arm A) and with mFOLFOX alone (Arm B). The sample size is chosen according to clinical reasoning. No statistical hypothesis testing is planned. A sample size of 50 patients to be treated with FLOT plus Nivolumab is regarded sufficient to get a first sight into the efficacy and to obtain data on the feasibility, safety and toxicity of the the study treatment.

As our study generally aims at gaining insights into the potentially most optimal chemo-immunotherapy regimen (therapy optimization) for a future trial, the implementation of Arm C fits well into the concept of the trial. With Arm C our trial evaluates three variations for the concept of immunochemotherapy: chemotherapy doublet plus immunotherapy doublet administered in parallel, chemotherapy doublet plus immunotherapy doublet administered sequentially, and chemotherapy triplet plus immune monotherapy administered in parallel.

Because Arms A and B are fully recruited, it is not possible to perform a randomized comparison for Arm C. However, using the patients from Arms A and B as a comparator is still better than historical controls as these patients are treated by the same centres, under the same therapeutic and diagnostic guidelines in a close time period.

The KEYNOTE-062 study (Tabernero et al. 2019) reported a median PFS of 6.9 months for the combination of Pembrolizumab plus chemotherapy while the Checkmate 649 (Janjigian et al. 2021) reported a median PFS of 7.7 months for the combination of Nivolumab plus mFOLFOX. Therefore, we expect the true median PFS of FLOT plus Nivolumab to be between 8 and 9 months corresponding to PFS rates at 6 months of about 59% and 63%.

The final conclusion for the combination of FLOT plus Nivolumab will depend on the definite PFS rate at 6 months (and its confidence interval), the respective findings in the arms A and B, as well as the information on type, frequency and severity of toxicities. The precision of the estimation of the PFS@6 is provided by confidence intervals (CIs) in the following table, for different actual PFS@6 findings:

PFS@6	Exact 95% CI
29/50 (58%)	43.2% … 71.8%
30/50 (60%)	45.2% … 73.6%
31/50 (62%)	47.2% … 75.4%
32/50 (64%)	49.2% … 77.1%

10.2 Populations for Analysis

All patients receiving at least one dose of study treatment will be evaluable for safety and included in the safety population.

The Intention-to-treat (ITT) population will include all randomized or enrolled (Arm C) patients in the study.

Since the only difference between Arms A and A1 is the study period in which the patients are recruited in the corresponding Arms, the data of A and A1 can be pooled for explorative analyses if the baseline characteristics are balanced. Therefore, A_{pooled} (A+A1) can be used for

for all secondary endpoints in comparison with B and A2, respectively. Additionally A2 will be compared to A1 and B for all secondary endpoints.
If the baseline characteristics are not balanced between Arms A and A1, these data cannot be pooled. Consequently, for all secondary endpoints following comparisons will be of interest: A vs. B, A1 vs. A2 and A2 vs. B.

The results of Arm C will be compared descriptively with the results of Arm A and B.

10.3 Patient Demographics/Other Baseline Characteristics

The following demographic and baseline characteristics will be summarized descriptively by treatment group and in total:

- Gender and age
- ECOG performance status
- Location of the primary
- Histopathology (e.g. Lauren's type, grading)
- Disease status (type and number of organs involved)
- Molecular background (e.g. PD-L1)
- Other characteristics

10.4 Treatments (study treatments)

The number and dose of treatment cycles will be summarized by treatment group and in total.

10.5 Efficacy Analysis

10.5.1 Primary Efficacy Endpoint

Primary endpoint (Arms A and B):

Progression Free Survival according to RECIST v1.1

Time to event distributions will be estimated using Kaplan Meier techniques. This will be done for PFS based on investigator assessments according to RECIST 1.1). Median PFS along with 95% CI and HR for PFS along with 95% CI will be calculated.

Hypothesis testing will be performed with a one-sided log-rank test at a one-sided significance level of 0.1 (s. sample size calculation).

Primary endpoint Arm A2 and C:
Six months progression-free survival rate (PFS@6)

10.5.2 Secondary Efficacy Endpoints

The secondary efficacy endpoints will be the following variables:

- Progression Free Survival acc. to RECIST v1.1 for Arms A1, A2 and C
- Progression Free Survival rate at 6 months (PFS@6) for Arms A and B
- Overall Response Rate (ORR) according to RECIST v1.1
- Duration of response and disease stabilization
- Overall survival (OS)
- Subgroup analysis including PFS and OS by PD-L1 expression status
- Safety and tolerability
- Quality of life assessment (EORTC QLQ-C30)
- Translational research aiming at defining predictive parameters

For the time-to-event variables, the Kaplan-Meier method will be used and treatment groups will be compared using a log rank test. All resulting p-values for secondary endpoints will be considered descriptive and no p-value adjustment for multiple-testing will be performed.

Response rate, safety and tolerability will be documented in a descriptive way. Continuous variables will be compared using t-test and categorical variables using a Chi-square test.

All secondary efficacy analyses, excluding toxicity, which will be based on the safety population, will be based on the ITT population.

10.5.3 Safety analyses (toxicity)

Data from all subjects who receive one or more doses of study treatment will be incorporated into the safety analyses. Study treatment exposure will be summarized. Adverse events, vital sign measurements, ECOG performance status, clinical laboratory information, and concomitant medications will be tabulated and summarized by group. All toxicities will be summarized by relative and absolute frequency, severity grade based on the CTCAE Version 4.03. Serious adverse events (SAE) will be listed separately. Safety information obtained during the Follow-up period during each segment will be incorporated into these analyses. Graphical displays will be provided where useful in the interpretation of results.

11. Data management

11.1 Randomization/Enrolment Procedure

Randomization/enrolment to study treatment should occur within seven days after eligibility criteria have been met. Upon confirmation of eligibility, study subjects will be randomized centrally 1:1 to arm A (modified FOLFOX plus Nivolumab and Ipilimumab) or B (modified FOLFOX) during enrolment of the first 118 patients or, after completion of enrolment of the

first 118 patients, 1:2 to arm A1 (therapy schema identical to arm A) or A2 (sequential therapy of FOLFOX followed by Nivolumab and Ipilimumab) according to the above mentioned stratification factors. For the subsequent study phase consisting of Arm C (FLOT cohort), study subjects will be formally enrolled via eCRF upon confirmation of eligibility.

11.2 Patient identification list

All included patients have to be documented in a confidential patient identification list. This list contains the patient specific numbers (patient- and randomization/enrolment-number) together with date of birth and the full name of the patient. Patient related data will be just transmitted in pseudonymized form. The identification list will stay at each center.

11.3 Data capture

All data will be entered directly at the center by the site staff with remote data entry (RDE). A study-management software will be used for data capture and query management. Automatic edit checks will validate data directly during entry into the study database. Data will be evaluated for consistency, accuracy and completeness regularly. After completion of data capture data-base will be closed and the data will be transferred into the statistic software.

12. Quality assurance

12.1 Standardization

Criteria for assessing efficacy and safety endpoints will be standardized by using NCI-CTCAE Version 4.03 for safety issues, RECIST Version 1.1 for efficacy parameters. Every center has to reveal their laboratory norm values and their validation through certification.

12.2 Data access

All source data have to be in the patients file under the responsibility of the investigator. Documentation in the eCRF must correspond to source data in the patient file. For this trial source data are defined as:

- medical and demographical data
- results of laboratory and imaging data
- selection criteria
- signed informed consent form (original)

12.3 Monitoring/ Source Data Verification (SDV)

The monitoring will be conducted according to local requirements.

Monitoring will be performed by the sponsor´s monitors. The study monitor will review the CRF data for completeness and accuracy during the monitoring visits (source data verification

/ SDV). The study monitor will point out any discrepancies between source data and the data captured in the CRF. The monitor will issue electronic queries to site staff to initiate discrepancy resolution. Discrepancies which require CRF data corrections have to be re-solved by authorized site personnel by answering these monitoring queries.

The frequency of on-site visits will depend on the number of recruited patients and results of prior monitorings (risk-adapted monitoring). The monitor must be given access to subject medical records and other study-related records needed to verify the entries on the CRF. The investigator agrees to cooperate with the monitor to ensure that any problems detected in the course of these monitoring visits, including delays in completing case report forms, are resolved. The investigator has to ensure that all data required according to this protocol will be entered promptly in the CRF.

Quality control of data will be done by reviewing the data entered into the trial software for consistency, accuracy and completeness. During on-site visits the correct transmission of data into the CRF (source data verification) as well as informed consent forms, selection criteria, efficacy and safety parameters will be reviewed. The complete scale of the monitoring will be defined by the trial specific monitoring plan.

12.4 Audits and Inspections

To ensure quality of data, study integrity, and compliance with the protocol and the various applicable regulations and guidelines, the sponsor/sponsor representative may conduct site visits to institutions participating to protocols.

The investigator, by accepting to participate to this protocol, agrees to co-operate fully with any quality assurance visit undertaken by third parties, including representatives from the sponsor, national and/or foreign regulatory authorities or company supplying the product under investigation, as well as to allow direct access to documentation pertaining to the clinical trial (including CRFs, source documents, hospital subject charts and other study files) to these authorized individuals.

The investigator must inform the sponsor/sponsor representative immediately in case a regulatory authority inspection will be scheduled.

13. Regulatory and Legal Obligations

13.1 General provisions/Declaration of Helsinki

This study is conducted in agreement with the ICH Harmonized Tripartite Guideline on Good Clinical Practice, valid since 17.01.1997, the Declaration of Helsinki (in its current version)) and the respective national laws in its current version). The Principle Investigator has more than two years of experience in the conduction of clinical drug trials.

13.2 Patient Protection

The responsible investigator will ensure that this study is conducted in agreement with either the Declaration of Helsinki (in its current version) or the laws and regulations in its current version.

The protocol has been written, and the study will be conducted according to the ICH Harmonized Tripartite Guideline for Good Clinical Practice. The protocol will be approved by Independent Ethics Committees.

13.3 Competent authority

Prior to the start of the trial an application for authorization by the Competent Authority (CA) is submitted by the sponsor/sponsor representative including a copy of the protocol and other information and documents required by the national Competent Authority. A copy of the written approval must be available before the start of recruitment of subjects into the study. All changes of the study protocol or other study document classified "substantial" as well as adverse events will be announced to the CA (according to the appropriate Directives and national legal requirements). Once a year or whenever it is questioned the CA will get information about all SAR and about the security of the affected subjects, according to the appropriate Directives and national legal requirements. Recommendations and tips of the CA will be taken up into the study protocol. The sponsor/sponsor representative will inform the CA about the course of the investigation in security aspects according to the appropriate Directives and national legal requirements and also about the end and the results of the investigation.

13.4 Independent Ethics Committee

Prior to the start of the trial an application for the favorable opinion for Germany is submitted on behalf of the sponsor to the central independent, interdisciplinary ethics committee responsible under federal law for the coordinating investigator and to the local ethics committees responsible for the other participating institutions including a copy of the protocol, proposed informed consent form and other information and documents required by the ethics committees for their opinion. A copy of the written favorable opinion of the protocol and informed consent form must be available before the start of recruitment of subjects into the study. All changes of the study protocol or other study document classified "substantial" as well as adverse events, will be announced to the Independent Ethics Committee (IEC), according to the local requirements, e.g. for Germany §13, (2) und (3) GCP-V. Once a year or whenever it is questioned the IEC will get information about all SAR and about the security of the affected subjects, (e.g. according to §13. (6) GCP-V). Recommendations of the IEC will be taken up into the study protocol. The sponsor/sponsor representative will inform the IEC about the course of the Investigation in security aspects (e.g. according §13 GCP-V, (1) till (6)) and also about the end and the results of the investigation (e.g. according to §13 GCP-V, (8) and (9)).

The investigator cannot influence the decisions of the IEC. A list of the IEC members will be ordered.

13.5 Amendments

The appendices, attached to this protocol and referred to in the protocol, form an integral part of the protocol. No changes or amendments to this protocol may be made by the Investigator. The sponsor/sponsor representative must submit and obtain favorable opinion/approval from the IEC and Competent Authority for all subsequent protocol amendments. For changes to the informed consent form favorable opinion from the IEC might be necessary.

13.6 Study Reports

Within one year after the end of the trial a clinical trial report will be written and provided to the IEC and Competent Authority independent of the completion or a premature closure of the trial.

13.7 Informed Consent

The informed consent form will be submitted together with the study protocol to the independent ethics committees (IEC) for review and approval. If requested, modifications must be incorporated. A copy of the written approval of the IEC must be available before starting the trial and dispensing any trial medication to trial subjects. The informed consent form must not be altered by the investigator except for contact data of the investigators. Changes to the informed consent form also have to be approved by the IEC. The revised form will be sent to all sites to replace the preceding version.

Before a subject's participation in the clinical study, the investigator must obtain written informed consent from the subject. All subjects will be informed of the aims of the study, the possible adverse events, the anticipated benefits, the procedures and possible hazards to which he/she will be exposed, and the mechanism of treatment allocation the subjects also will be informed about alternative treatments. Subjects will be informed of their insurance protection and the obligations which are linked to insurance. They will be informed as to the strict confidentiality of their subject data, but that their medical records may be reviewed for trial purposes by authorized individuals other than their treating physician. It will be emphasized that the participation is voluntary and that the subject is allowed to refuse further participation in the protocol whenever he/she wants. This will not prejudice the subject's subsequent care. The informed consent procedure must conform to the ICH guidelines on Good Clinical Practice.

The informed consent consists of three parts: consent to the diagnostic and therapeutic procedures of the trial, consent to the collection and storage of biological material, and consent to the processing and storage of data. The latter one includes consent to inspections where records may be reviewed by authorized individuals (other than their treating physician) of the sponsor or surveillance authorities / ethics committees. If the subject does not consent to the collection, processing and storage of his data, inclusion in the study is not possible and the subject's refusal should be documented in the medical notes. The subject must be informed about the aims, methods, anticipated benefits, and potential hazards of the study and before any

protocol-specific screening procedures or any study treatment are administered. The collection and storage of biological material in this clinical trial is optional; consent to this part of the trial is not necessary for the participation in this clinical trial.

The investigator is also responsible for asking the subject if the subject agrees to have his/her primary care physician informed of the subject's participation in the clinical study. If the subject agrees to such notification, the investigator shall inform the subject's primary care physician of the subject's participation in the clinical study.

If a potential subject is illiterate or visually impaired, the investigator must provide an impartial witness to read the informed consent form to the subject and must allow for questions. Thereafter, both the subject and the witness must sign the informed consent form to attest that informed consent was freely given and understood.

Adequate explanations of the aims, methods, anticipated benefits, and potential hazards of the study, the mechanism of treatment allocation must be given. The subject will have enough time to decide to participate in the study or not.

The acquisition of informed consent and the subject's agreement or refusal of his/her notification of the primary care physician must be documented in the subject's medical records, and the informed consent form must be signed and personally dated by the subject and by the investigator. One signed original of the informed consent form must be retained in accordance with institutional policy and another original must be provided to the subject. Treatment cannot start before the subject has signed the informed consent, meets all inclusion and no exclusion criteria and is registered.

With signing the informed consent form the investigator confirms that an individual clarification conversation has taken place and that the subject has signed the informed consent form.

13.8 Subject Confidentiality

The investigator must ensure that the subject's confidentiality is maintained. On the case report forms, subjects should be identified by their subject study number and only on the SAE report form additionally the age.

In compliance with ICH-GCP Guidelines, it is required that the investigator and institution permit authorized representatives of the sponsor, and of regulatory agencies direct access to review the subject's original medical records for verification of study-related procedures and data. Direct access includes examining, analyzing, verifying, and reproducing any records and reports that are important to the evaluation of the study. The investigator is obligated to inform and obtain the consent of the subject to permit named representatives to have access to his/her study-related records without violating the confidentiality of the subject. The investigator must keep a list for the identification of the subjects (including name, birthday, gender, date of informed consent, date of randomization /enrolment / registration).

13.9 Study Documentation and Archive

The investigator must maintain a list of appropriately qualified persons to whom he/she has delegated study duties, including all those authorized to make entries and/or corrections on case report forms.

Source documents are original documents, data, and records from which the subject's case report form data are obtained. These include but are not limited to hospital records, clinical and office charts, laboratory and pharmacy records, diaries, microfiches, radiographs, and correspondence.

The investigator and study staff are responsible for maintaining a comprehensive and centralized filing system of all study-related (essential) documentation, suitable for inspection at any time by representatives from the study sponsor and/or applicable regulatory authorities. Elements include:

- Subject files containing completed case report forms, informed consent forms, and subject identification list.
- Study files containing the protocol with all amendments, the summary of product characteristics, copies of pre-study documentation, and all correspondence to and from the IEC.
- If kept, proof of receipt, Investigational Product Accountability Record, Return of Investigational Product for Destruction, Final Investigational Product Reconciliation Statement, and all drug-related correspondence.

In addition, all original source documents supporting entries in the case report forms must be maintained and be readily available.

All study documents and source documents must be kept for at least 10 years from submission of the final study report. Should the investigator wish to assign the study records to another party or move them to another location, he/she must notify the sponsor/sponsor representative in writing of the new responsible person and/or the new location.

13.10 Compensation

Subjects will not be paid for participating in this clinical trial.

14. Trial Sponsorship and Financing

The Institut für Klinische Krebsforschung IKF GmbH at Krankenhaus Nordwest is the legal sponsor of the trial and finances the trial. Financial and material support for the conduction of the trial is granted by Bristol-Myers Squibb.

15. Trial Insurance

For all subjects participating in the trial the sponsor has taken out a liability insurance policy (mentioned below) according to the respective national law (e.g. § 40 (1) Nr. 8 and (3) German drug law (AMG)) which covers the sponsor, the investigator and his co-workers against liability in the event that a subject's health is injured during the course of the clinical trial. The insurance policy provides benefits, even when no one else is liable for the damage death of or injury to any subject during the trial.

A certificate of insurance and conditions will be provided to the investigators and the subjects. The clinical trials insurance was contracted at HDI Global SE, Ganghoferstraße 37–39, 80339 München with the contract no. 33-242271-03010/390.

16. Trial Registration

The trial is registered at ClinicalTrials.gov before start of the study (FPI).

17. Publication Policy

After receiving the biometrical results, a final report will be published and further publications (abstracts etc.) will be done. Both leading investigators, Sylvie Lorenzen and Thorsten Götze define the authors of the publications and the order within authors's listing. Further details will depend on international guidelines for authorship and the policy of the target journal.

Appendix A: Bibliography

Al-Batran SE, Hartmann JT, Probst S, Schmalenberg H, Hollerbach S, Hofheinz R, Rethwisch V, Seipelt G, Homann N, Wilhelm G, Schuch G, Stoehlmacher J, Derigs HG, Hegewisch-Becker S, Grossmann J, Pauligk C, Atmaca A, Bokemeyer C, Knuth A, Jäger E; Arbeitsgemeinschaft Internistische Onkologie. Phase III trial in metastatic gastroesophageal adenocarcinoma with fluorouracil, leucovorin plus either oxaliplatin or cisplatin: a study of the Arbeitsgemeinschaft Internistische Onkologie. J Clin Oncol. 2008 Mar 20;26(9):1435-42. doi: 10.1200/JCO.2007

Bang YJ, Muro K, Fuchs CS, Golan T, Geva R, Hara H, Jalal SI, Borg C, Doi T, Wainberg ZA, Wang J, Koshiji M, Dalal RP, Chung HC. KEYNOTE-059 cohort 2: Safety and efficacy of pembrolizumab (pembro) plus 5-fluorouracil (5-FU) and cisplatin for first-line (1L) treatment of advanced gastric cancer. J Clin Oncol 35, 2017 (suppl; abstr 4012)

Borghaei H, Paz-Ares L, Horn L, et al. Nivolumab versus Docetaxel in Advanced Nonsquamous Non-Small-Cell Lung Cancer. N Engl J Med. 2015 Oct 22;373(17):1627-39.

Brahmer, J., K. L. Reckamp, P. Baas, L. Crino, W. E. Eberhardt, E. Poddubskaya, S. Antonia, A. Pluzanski, E. E. Vokes, E. Holgado, D. Waterhouse, N. Ready, J. Gainor, O. Aren Frontera, L. Havel, M. Steins, M. C. Garassino, J. G. Aerts, M. Domine, L. Paz-Ares, M. Reck, C. Baudelet, C. T. Harbison, B. Lestini and D. R. Spigel (2015). "Nivolumab versus Docetaxel in Advanced Squamous-Cell Non-Small-Cell Lung Cancer." N Engl J Med **373**(2): 123-135.

Buas, M. F. and T. L. Vaughan (2013). "Epidemiology and risk factors for gastroesophageal junction tumors: understanding the rising incidence of this disease." Semin Radiat Oncol **23**(1): 3-9.

CA209067: A Phase 3, Randomized, Double-Blind Study of Nivolumab Monotherapy or Nivolumab Combined with Ipilimumab Versus Ipilimumab Monotherapy in Subjects with Previously Untreated Unresectable or Metastatic Melanoma. Document Control Number: 930067689.

Cunningham D, Starling N, Rao S, et al. Capecitabine and Oxaliplatin for Advanced Esophagogastric Cancer. N Engl J Med 2008;358:36-46.

Curran, M. A., W. Montalvo, H. Yagita and J. P. Allison (2010). "PD-1 and CTLA-4 combination blockade expands infiltrating T cells and reduces regulatory T and myeloid cells within B16 melanoma tumors." Proc Natl Acad Sci U S A **107**(9): 4275-4280.

Davis et al NCCN Network Annual Conference and Abrams, ESMO GI 2016# PD-034)

Eisenhauer, E. A., P. Therasse, J. Bogaerts, L. H. Schwartz, D. Sargent, R. Ford, J. Dancey, S. Arbuck, S. Gwyther, M. Mooney, L. Rubinstein, L. Shankar, L. Dodd, R. Kaplan, D. Lacombe and J. Verweij (2009). "New response evaluation criteria in solid tumours: revised RECIST guideline (version 1.1)." Eur J Cancer **45**(2): 228-247.

Ferlay, J., I. Soerjomataram, R. Dikshit, S. Eser, C. Mathers, M. Rebelo, D. M. Parkin, D. Forman and F. Bray (2015). "Cancer incidence and mortality worldwide: sources, methods and major patterns in GLOBOCAN 2012." Int J Cancer **136**(5): E359-386.

Ferlay, J., E. Steliarova-Foucher, J. Lortet-Tieulent, S. Rosso, J. W. Coebergh, H. Comber, D. Forman and F. Bray (2013). "Cancer incidence and mortality patterns in Europe: estimates for 40 countries in 2012." Eur J Cancer **49**(6): 1374-1403.

Fleming TR: One-sample multiple testing procedure for phase II clinical trials. Biometrics 1982, 38: 143-151

Fuchs CS, Doi T, Jang RW, et al. Safety and Efficacy of Pembrolizumab Monotherapy in Patients With Previously Treated Advanced Gastric and Gastroesophageal Junction Cancer: Phase 2 Clinical KEYNOTE-059 Trial. JAMA Oncol 2018; 4: e180013

Janjigian, Y. Y., J. C. Bendell and E. Calvo (2016). "CheckMate-032: Phase I/II, open-label study of safety and activity of nivolumab (nivo) alone or with ipilimumab (ipi) in advanced and metastatic (A/M) gastric cancer (GC)." J Clin Oncol **34**(suppl): abstr 4010.

Janjigian YY, Shitara K, Moehler M, Garrido M, Salman P, Shen L, Wyrwicz L, Yamaguchi K, Skoczylas T, Campos Bragagnoli A, Liu T, Schenker M, Yanez P, Tehfe M, Kowalyszyn R, Karamouzis MV, Bruges R, Zander T, Pazo-Cid R, Hitre E, Feeney K, Cleary JM, Poulart V, Cullen D, Lei M, Xiao H, Kondo K, Li M, Ajani JA. First-line nivolumab plus chemotherapy versus chemotherapy alone for advanced gastric, gastro-oesophageal junction, and oesophageal adenocarcinoma (CheckMate 649): a randomised, open-label, phase 3 trial. Lancet. 2021 Jun 4:S0140-6736(21)00797-2. doi: 10.1016/S0140-6736(21)00797-2. Epub ahead of print. PMID: 34102137.

Japanese Gastric Cancer Association. Japanese gastric cancer treatment guidelines 2010 (ver. 3).Gastric Cancer 2011;14:113-123.

Jatoi A, Murphy BR, Foster NR, et al. Oxaliplatin and capecitabine in patients with metastatic adenocarcinoma of the esophagus, gastroesophageal junction and gastric cardia: a phase II study from the North Central Cancer Treatment Group. Ann Oncol 2006;17:29.

Kang H, Kauh J. Chemotherapy in the Treatment of Gastric Cancer: Is there a global standard? Current Treatment Options in Oncology 2011;12:96-106.

Kang YK, Kato K., Chung, CH. Interim safety and clinical activity of nivolumab in combination with S-1/capecitabine plus oxaliplatin in patients (pts) with previously untreated unresectableadvanced or reccurent gastric /gastroesophageal junction (G/GEJ) cancer: part 1 study of ATTRACTION -04 (ONO-4538-37). Ann Oncol 2017; 28 suppl 5: v209–268.

Kato, K, J. Sun, M.A. Shah, P.C. Enzinger, A. Adenis, T. Doi, T. Kojima, J. Metges, Z. Li, S. Kim, B.C. Chul Cho, W. Mansoor, S. Li, P. Sunpaweravong, M.A. Maqueda, E. Goekkurt, Q. Liu, S. Shah, P. Bhagia, L. Shen. Pembrolizumab plus chemotherapy versus chemotherapy as first-line therapy in patients with advanced esophageal cancer: The phase 3 KEYNOTE-590 study. Annals of Oncology (2020) 31 (suppl_4): S1142-S1215. 10.1016/annonc/annonc325

Kershaw MH, Devaud C, John LB, Westwood JA, Darcy PK. Enhancing immunotherapy using chemotherapy and radiation to modify the tumor microenvironment. Oncoimmunology. 2013 Sep 1;2(9):e25962

Langer CJ, Gadgeel SM, Borghaei H, Papadimitrakopoulou VA, Patnaik A, Powell SF, Gentzler RD, Martins RG, Stevenson JP, Jalal SI, Panwalkar A, Yang JC, Gubens M, Sequist LV, Awad MM, Fiore J, Ge Y, Raftopoulos H, Gandhi L; KEYNOTE-021 investigators. Carboplatin and pemetrexed with or without pembrolizumab for advanced, non-squamous non-small-cell lung cancer: a randomised, phase 2 cohort of the open-label KEYNOTE-021 study. Lancet Oncol. 2016 Nov;17(11):1497-1508.

Larkin, J., V. Chiarion-Sileni, R. Gonzalez, J. J. Grob, C. L. Cowey, C. D. Lao, D. Schadendorf, R. Dummer, M. Smylie, P. Rutkowski, P. F. Ferrucci, A. Hill, J. Wagstaff, M. S. Carlino, J. B. Haanen, M. Maio, I. Marquez-Rodas, G. A. McArthur, P. A. Ascierto, G. V. Long, M. K. Callahan, M. A. Postow, K. Grossmann, M. Sznol, B. Dreno, L. Bastholt, A. Yang, L. M. Rollin, C. Horak, F. S. Hodi and J. D. Wolchok (2015). "Combined Nivolumab and Ipilimumab or Monotherapy in Untreated Melanoma." N Engl J Med **373**(1): 23-34.

Moehler M, Janjigian, Y.Y., Adenis, A. CheckMate 649: A randomized, multicenter, open-label, phase III study of nivolumab(nivo) plus ipilimumab (ipi) or nivo + chemotherapy (CTX) vs CTX alone in pts with previously untreated advanced gastric or gastroesophageal junction (GEJ) cancer.J Clin Oncol. 2017; 35(15-suppl15):TPS4132.

Montagnani F, Turrisi G, Marinozzi C Effectiveness and safety of oxaliplatin compared to cisplatin for advanced, unresectable gastric cancer: a systematic review and meta-analysis. Gastric Cancer. 2011;14(1):50.

Motzer, R. J., B. Escudier, D. F. McDermott, S. George, H. J. Hammers, S. Srinivas, S. S. Tykodi, J. A. Sosman, G. Procopio, E. R. Plimack, D. Castellano, T. K. Choueiri, H. Gurney, F. Donskov, P. Bono, J. Wagstaff, T. C. Gauler, T. Ueda, Y. Tomita, F. A. Schutz, C. Kollmannsberger, J. Larkin, A. Ravaud, J. S. Simon, L. A. Xu, I. M. Waxman, P. Sharma and I. CheckMate (2015). "Nivolumab versus Everolimus in Advanced Renal-Cell Carcinoma." N Engl J Med **373**(19): 1803-1813.

Muro, K., H. C. Chung, V. Shankaran, R. Geva, D. Catenacci, S. Gupta, J. P. Eder, T. Golan, D. T. Le, B. Burtness, A. J. McRee, C. C. Lin, K. Pathiraja, J. Lunceford, K. Emancipator, J. Juco, M. Koshiji and Y. J. Bang (2016). "Pembrolizumab for patients with PD-L1-positive advanced gastric cancer (KEYNOTE-012): a multicentre, open-label, phase 1b trial." Lancet Oncol.

NCCN guideline Gastric cancer (Ver 3.0, 2015). http://www.nccn.org/professionals/physician_gls/f_guidelines_nojava.asp.Accessed: 18-Mar-2016.

Ohigashi Y, Sho M, Yamada Y et al. Clinical significance of programmed death-1 ligand-1 and programmed death-1 ligand-2 expression in human esophageal cancer. Clin Cancer Res. 2005;11(8):2947-53.

Postow, M. A., J. Chesney, A. C. Pavlick, C. Robert, K. Grossmann, D. McDermott, G. P. Linette, N. Meyer, J. K. Giguere, S. S. Agarwala, M. Shaheen, M. S. Ernstoff, D. Minor, A. K. Salama, M. Taylor, P. A. Ott, L. M. Rollin, C. Horak, P. Gagnier, J. D. Wolchok and F. S. Hodi (2015). "Nivolumab and Ipilimumab versus Ipilimumab in Untreated Melanoma." N Engl J Med.

Rizvi N, Hellmann M, Brahmer J, et al. Nivolumab in Combination With Platinum-Based Doublet Chemotherapy for First-Line Treatment of Advanced Non–Small-Cell Lung Cancer. Clin Oncol 2016, 34:2969-2979.

Smyth, E. C., M. Verheij, W. Allum, D. Cunningham, A. Cervantes, D. Arnold and E. G. Committee (2016). "Gastric cancer: ESMO Clinical Practice Guidelines for diagnosis, treatment and follow-up." Ann Oncol **27**(suppl 5): v38-v49.

Tabernero J VCE, Bang YJ. Pembrolizumab with or without chemotherapy versus chemotherapy for advanced gastric or gastroesophageal junction (G/GEJ) adenocarcinoma: The phase III KEYNOTE-062 study. J Clin Oncol 2019;37 (suppl; abstr LBA4007).

van Meerten E, Eskens FA, van Gameren EC, et al. First-line treatment with oxaliplatin and capecitabine in patients with advanced or metastatic oesophageal cancer: a phase II study. Br J Cancer 2007;96:1348.

Van Cutsem E, Moiseyenko VM, Tjulandin S, Majlis A, Constenla M, Boni C, Rodrigues A, Fodor M, Chao Y, Voznyi E, Risse ML, Ajani JA; V325 Study Group. J Clin Oncol. 2006 Phase III study of docetaxel and cisplatin plus fluorouracil compared with cisplatin and fluorouracil as first-line therapy for advanced gastric cancer: a report of the V325 Study Group. Nov 1;24(31):4991-7. doi: 10.1200/JCO.2006.06.8429

Waddell T, Verheij M, Allum W, et al. Gastric cancer†: ESMO–ESSO–ESTRO Clinical Practice Guidelines for diagnosis, treatment and follow-up. Ann Oncol (2013) 24 (suppl 6): vi57-vi63

Wagner, A. D., S. Unverzagt, W. Grothe, G. Kleber, A. Grothey, J. Haerting and W. E. Fleig (2010). "Chemotherapy for advanced gastric cancer." Cochrane Database Syst Rev **3**: CD004064.

Wagner A.D., Syn N.L., Moehler M., Grothe W., Yong W.P., Tai B-C, Ho J., Unverzagt S.. Chemotherapy for advanced gastric cancer. Cochrane Database Syst Rev.2017 Aug 29;8(8):CD004064. doi: 10.1002/14651858.CD004064.pub4.

Wolchok, J. D., A. Hoos, S. O'Day, J. S. Weber, O. Hamid, C. Lebbe, M. Maio, M. Binder, O. Bohnsack, G. Nichol, R. Humphrey and F. S. Hodi (2009). "Guidelines for the evaluation of immune therapy activity in solid tumors: immune-related response criteria." Clin Cancer Res **15**(23): 7412-7420.

Wu C, Zhu Y, Jiang J et al. Immunohistochemical localization of programmed death-1 ligand-(PD-L1) in gastric carcinoma and its clinical significance. Acta Histochem. 2006;108(1):19-24.

Yamada Y, Higuchi K, Nishikawa K, et al. Phase III study comparing oxaliplatin plus S-1 with cisplatin plus S-1 in chemotherapy-naïve patients with advanced gastric cancer. Annals of Oncology 2015;26:141-148.

Appendix B: ECOG Performance Status

Grade	Performance scale
0	Able to carry out all normal activity without restriction
1	Restricted in physically strenuous activity but ambulatory and able to carry out light work.
2	Ambulatory and capable of all self-care but unable to carry out any work; up and about more than 50% of waking hours.
3	Capable of only limited self-care; confined to bed or chair more than 50% of waking hours
4	Completely disabled; cannot carry on any self-care; totally confined to bed or chair.

Appendix C: Common Terminology Criteria for Adverse Events (CTCAE)

In the present study, adverse events and/or adverse drug reactions will be recorded according to the Common Terminology Criteria for Adverse Events (CTCAE), version 4.03.

At the time this protocol was issued, the full CTC document was available on the NCI web site, at the following address: http://evs.nci.nih.gov/ftp1/CTCAE/CTCAE_4.03_2010-06-14_QuickReference_5x7.pdf

Another option is via the EORTC Headquarters web site www.eortc.be, which provides a link to the appropriate CTC web site. This link will be updated if the CTC address is changed.

Appendix D: Response Evaluation Criteria in Solid Tumors

Conventional response criteria may not be adequate to characterize the anti-tumor activity of immunotherapeutic agents like nivolumab and ipilimumab, which can produce delayed responses that may be preceded by initial apparent radiological progression, including the appearance of new lesions. Therefore, modified response criteria have been developed that account for the possible appearance of new lesions and allow radiological progression to be confirmed at a subsequent assessment. In this protocol, patients will be permitted to continue study treatment even after modified Response Evaluation Criteria in Solid Tumors (RECIST) criteria for progressive disease are met if the risk/benefit ratio is judged to be favorable.

Table 8: RECIST, Version 1.1: Summary of Changes

	RECIST v1.1
New lesions after baseline	Define progression.
Non-target lesions	May contribute to the designation of overall progression
Radiographic progression	First instance of > 20% increase in the sum of diameters or unequivocal progression in non-target disease

RECIST: Response Evaluation Criteria in Solid Tumors.

DEFINITIONS OF MEASURABLE/NON-MEASURABLE LESIONS

All measurable and non-measurable lesions should be assessed at screening and at the protocol-specified tumor assessment timepoints. Additional assessments may be performed, as clinically indicated for suspicion of progression. The investigator will evaluate response to treatment using RECIST.

MEASURABLE LESIONS

Tumor Lesions. Tumor lesions must be accurately measured in at least one dimension (longest diameter in the plane of measurement is to be recorded) with a minimum size as follows:

≥10 mm by computed tomography (CT) or magnetic resonance imaging (MRI) scan (CT/MRI scan slice thickness/interval no greater than 5 mm)

10-mm caliper measurement by clinical examination (lesions that cannot be accurately measured with calipers should be recorded as non-measurable)

Malignant Lymph Nodes. To be considered pathologically enlarged and measurable, a lymph node must be ≥15 mm in the short axis when assessed by CT scan (CT scan slice thickness recommended to be no greater than 5 mm). At baseline and follow-up, only the short axis will be measured and followed.

NON-MEASURABLE LESIONS

Non-measurable tumor lesions encompass small lesions (longest diameter 10 mm or pathological lymph nodes with short axis ≥ 10 but ≤15 mm), as well as truly non-measurable lesions. Lesions considered truly non-measurable include leptomeningeal disease, ascites, pleural or pericardial effusion, inflammatory breast disease, lymphangitic involvement of skin or lung, peritoneal spread, and abdominal mass/abdominal organomegaly identified by physical examination that is not measurable by reproducible imaging techniques.

SPECIAL CONSIDERATIONS REGARDING LESION MEASURABILITY

Bone lesions, cystic lesions, and lesions previously treated with local therapy require particular comment, as outlined below.

BONE LESIONS

Bone scan, positron emission tomography (PET) scan, or plain films are not considered adequate imaging techniques for measuring bone lesions. However, these techniques can be used to confirm the presence or disappearance of bone lesions. Lytic bone lesions or mixed lytic–blastic lesions, with identifiable soft tissue components, that can be evaluated by cross-sectional imaging techniques such as CT or MRI can be considered as measurable lesions if the soft tissue component meets the definition of measurability described above. Blastic bone lesions are non-measurable.

CYSTIC LESIONS

Lesions that meet the criteria for radiographically defined simple cysts should not be considered as malignant lesions (neither measurable nor non-measurable) since they are, by definition, simple cysts. Cystic lesions thought to represent cystic metastases can be considered as measurable lesions, if they meet the definition of measurability described above. However, if non-cystic lesions are present in the same patient, these are preferred for selection as target lesions.

TUMOR RESPONSE EVALUATION

DEFINITIONS OF TARGET/NON-TARGET LESIONS

Target Lesions

When more than one measurable lesion is present at baseline, all lesions up to a maximum of five lesions total (and a maximum of two lesions per organ) representative of all involved organs should be identified as target lesions and will be recorded and measured at baseline. This means that, for instances in which patients have only one or two organ sites involved, a maximum of two lesions (one site) and four lesions (two sites), respectively, will be recorded. Other lesions (albeit measurable) in those organs will be recorded as non-measurable lesions (even if the size is 10 mm by CT scan).

Target lesions should be selected on the basis of their size (lesions with the longest diameter) and be representative of all involved organs, but in addition, should lend themselves to reproducible repeated measurements. It may be the case that, on occasion, the largest lesion does not lend itself to reproducible measurement, in which circumstance, the next largest lesion that can be measured reproducibly should be selected.

Lymph nodes merit special mention since they are normal anatomical structures that may be visible by imaging even if not involved by tumor. As noted above, pathological nodes that are defined as measurable and may be identified as target lesions must meet the criterion of a short axis of ≥15 mm by CT scan. Only the short axis of these nodes will contribute to the baseline sum. The short axis of the node is the diameter normally used by radiologists to judge if a node is involved by solid tumor. Nodal size is normally reported as two dimensions in the plane in which the image is obtained (for CT, this is almost always the axial plane; for MRI, the plane of acquisition may be axial, sagittal, or coronal). The smaller of these measures is the short axis. For example, an abdominal node that is reported as being 20 mm x 30 mm has a short axis of 20 mm and qualifies as a malignant, measurable node. In this example, 20 mm should be recorded as the node measurement. All other pathological nodes (those with short axis ≥ 10 mm but < 15 mm) should be considered non-target lesions. Nodes that have a short axis of < 10 mm are considered non-pathological and should not be recorded or followed. Lesions irradiated within 3 weeks prior to Cycle 1, Day 1 may not be counted as target lesions.

Non-Target Lesions

All other lesions (or sites of disease), including pathological lymph nodes, should be identified as non-target lesions and should also be recorded at baseline. Measurements are not required.

It is possible to record multiple non-target lesions involving the same organ as a single item on the Case Report Form (CRF) (e.g., “multiple enlarged pelvic lymph nodes” or “multiple liver metastases”).

After baseline, changes in non-target lesions will contribute only in the assessment of complete response (i.e., a complete response is attained only with the complete disappearance of all tumor lesions, including non-target lesions) and will not be used to assess progressive disease.

New Lesions

During the study, all new lesions identified and recorded after baseline must be assessed at all tumor assessment timepoints. New lesions will also be evaluated for measurability with use of the same criteria applied to prospective target lesions at baseline per RECIST, (e.g., non-lymph node lesions must be 10mm; see note for new lymph node lesions below). Up to a maximum of five new lesions total (and a maximum of two lesions per organ), all with measurements at all timepoints, can be included in the tumor response evaluation. New lesion types that would not qualify as target lesions per RECIST cannot be included in the tumor response evaluation.

New lesions that are not measurable at first appearance but meet measurability criteria at a subsequent timepoint will be measured from that point on and contribute to the sum of longest diameters (SLD), if the maximum number of 5 measurable new lesions being followed has not been reached.

CALCULATION OF SUM OF THE DIAMETERS

A sum of the diameters (longest for non-nodal lesions, short axis for nodal lesions) for all target lesions will be calculated as a measure of tumor burden.

The sum of the diameters is calculated at baseline and at each tumor assessment for the purpose of classification of tumor responses.

Sum of the Diameters at Baseline: The sum of the diameters for all target lesions identified at baseline prior to treatment on Day 1.

Sum of the Diameters at Tumor Assessment: For every on-study tumor assessment collected per protocol or as clinically indicated, the sum of the diameters at tumor assessment will be calculated using tumor imaging scans. All target lesions and all new measurable lesions that have emerged after baseline will contribute to the sum of the diameters at tumor assessment. Hence, each net percentage change in tumor burden per assessment with use of RECIST accounts for the size and growth kinetics of both old and new lesions as they appear.

Note: In the case of new lymph nodes, RECIST v1.1 criteria for measurability (equivalent to baseline target lesion selection) will be followed. That is, if at first appearance the short axis of a new lymph node lesion ≥ 15 mm, it will be considered a measureable new lesion and will be tracked and included in the SLD. Thereafter, the lymph node lesion will be measured at subsequent timepoints and measurements will be included in the SLD, even if the short axis diameter decreases to ≤15 mm (or even ≤ 10 mm). However, if it subsequently decreases to ≤ 10 mm, and all other lesions are no longer detectable (or have also decreased to a short axis diameter of ≤ 10 mm if lymph nodes), then a response assessment of CR may be assigned.

If at first appearance the short axis of a new lymph node is ≥ 10 mm and ≤ 15 mm, the lymph node will not be considered measurable but will still be considered a new lesion. It will not be included in the SLD unless it subsequently becomes measurable (short axis diameter ≥ 15 mm).

The appearance of new lymph nodes with diameter ≤ 10 mm should not be considered pathological and not considered a new lesion.

RESPONSE CRITERIA

Evaluation of Target Lesions

Complete Response (CR): Disappearance of all target lesions. Lymph nodes that shrink to ≤ 10 mm short axis are considered normal.

Partial Response (PR): At least a 30% decrease in the sum of the diameters of all target and all new measurable lesions, taking as reference the baseline sum of diameters, in the absence of CR.

Note: The appearance of new measurable lesions is factored into the overall tumor burden but does not automatically qualify as progressive disease until the sum of the diameters increases by ≥ 20% when compared with the sum of the diameters at nadir.

Stable Disease (SD): Neither sufficient shrinkage to qualify for PR nor sufficient increase to qualify for PD, taking as reference the smallest sum of the diameters while on study.

Progressive Disease (PD): At least a 20% increase in the sum of diameters of all target and all new measurable lesions, taking as reference the smallest sum on study (nadir SlD; this includes the baseline sum if that is the smallest on study). In addition to the relative increase of 20%, the sum must also demonstrate an absolute increase of at least 5 mm.

EVALUATION OF BEST OVERALL RESPONSE USING RECIST

TIMEPOINT RESPONSE

It is assumed that at each protocol-specified timepoint, a response assessment occurs.

MISSING ASSESSMENTS AND *NOT EVALUABLE* DESIGNATION

When no imaging/measurement is done at all at a particular timepoint, the patient is not evaluable (NE) at that timepoint. If only a subset of lesion measurements are made at an assessment, usually the case is also considered NE at that timepoint, unless a convincing argument can be made that the contribution of the individual missing lesion(s) would not change the assigned time point response. This would be most likely to happen in the case of PD. For example, if a patient had a baseline sum of 50 mm with three measured lesions and at follow-up only two lesions were assessed but those gave a sum of 80 mm, the patient will have achieved PD status, regardless of the contribution of the missing lesion.

Appendix E: Translational research working instructions

Contact/Questions:

Elke Veltrup
STRATIFYER Molecular Pathology GmbH
Werthmannstr. 1c
50935 Köln
Tel.: 0221 / 4677 – 2916
Fax: 0221/ 4677 – 2917
Email: elke.veltrup@STRATIFYER.de

Blood draw

- **Acquisition of blood in EDTA tubes**
    - Timepoint: prior to treatment
    - Blood will be collected in EDTA tubes a 2 mL and immediately shipped using the prelabbeled envelopes to

Dr. Ralph Wirtz
STRATIFYER
Molecular Pathology GmbH
Werthmannstr. 1c
50935 Köln
Germany

Lab kits, including the EDTA tubes, labels and working instructions will be provided.

Tissue

Obtain paraffin embedded tissue for PD-L1 assessment and translational research. Centers may send either paraffin block or slices. If slices are sent, please send 1x HE staines slice. 4 x 5 µm unstained tissue slices on glass slides The tissue will be immediately shipped using the prelabeled envelopes to

Institut für Klinische Krebsforschung IKF GmbH
at Krankenhaus Nordwest
Steinbacher Hohl 2-26
60488 Frankfurt
Germany
