# Supplementary material for: First-line modified FOLFOX plus/minus nivolumab and Ipilimumab or FLOT plus nivolumab in advanced gastroesophageal adenocarcinoma: a phase II multi-cohort IKF-AIO-MOONLIGHT trial
Source: Nat Commun. 2026 Feb 27;17:2072. doi: 10.1038/s41467-026-69622-7 (PMC12948978; doi:10.1038/s41467-026-69622-7)
Supplement: Supplementary file 2 — Description of Additional Supplementary Files [file 41467_2026_69622_MOESM2_ESM.pdf]

Description of Additional Supplementary Files

Supplementary Dataset 1: Table S2 Adverse events (whether related or not), max. grade by patient and category
